# Theoretical Study of the Iron Complexes with Lipoic and Dihydrolipoic Acids: Exploring Secondary Antioxidant Activity

**DOI:** 10.3390/antiox9080674

**Published:** 2020-07-28

**Authors:** Roger Monreal-Corona, Jesse Biddlecombe, Angela Ippolito, Nelaine Mora-Diez

**Affiliations:** Department of Chemistry, Thompson Rivers University, Kamloops, BC V2C 0C8, Canada; monrealcoronar18@mytru.ca (R.M.-C.); biddlecombej14@mytru.ca (J.B.); ippolitoa14@mytru.ca (A.I.)

**Keywords:** lipoic acid, dihydrolipoic acid, iron complexes, copper complexes, Haber–Weiss cycle, superoxide radical anion, ascorbate, Marcus theory

## Abstract

The thermodynamic stability of twenty-nine Fe(III) complexes with various deprotonated forms of lipoic (LA) and dihydrolipoic (DHLA) acids, with coordination numbers 4, 5 and 6, is studied at the M06(SMD)/6-31++G(d,p) level of theory in water under physiological pH conditions at 298.15 K. Even though the complexes with LA^-^ are more stable than those with DHLA^−^, the most thermodynamically stable Fe(III) complexes involve DHLA^2−^. The twenty-four exergonic complexes are used to evaluate the secondary antioxidant activity of DHLA and LA relative to the Fe(III)/Fe(II) reduction by $O_{2}^{•-}$ and ascorbate. Rate constants for the single-electron transfer (SET) reactions are calculated. The thermodynamic stability of the Fe(III) complexes does not fully correlate with the rate constant of their SET reactions, but more exergonic complexes usually exhibit smaller SET rate constants. Some Cu(II) complexes and their reduction to Cu(I) are also studied at the same level of theory for comparison. The Fe(III) complexes appear to be more stable than their Cu(II) counterparts. Relative to the Fe(III)/Fe(II) reduction with ascorbate, DHLA can fully inhibit the formation of ^•^OH radicals, but not by reaction with $O_{2}^{•-}$. Relative to the Cu(II)/Cu(I) reduction with ascorbate, the effects of DHLA are moderate/high, and with $O_{2}^{•-}$ they are minor. LA has minor to negligible inhibition effects in all the cases considered.

## 1. Introduction

Emotional stress and environmental pollution can lead to the excessive biochemical production of free radicals beyond the human system’s ability to detoxify reactive intermediates and repair the subsequent cellular damage [1]. This imbalance induces what is known as oxidative stress. There is strong evidence that oxidative stress is related to the development of numerous diseases such as arthritis, atherosclerosis, emphysema, cancer [2], Alzheimer’s disease [3] and eye diseases [4]. One of the health-restoring biochemical routes involves the action of antioxidants which can be classified according to the protection mechanisms they offer [5].

Primary antioxidants react with free radicals producing less reactive species unable to damage biological targets. Secondary antioxidants exert their protection without directly reacting with free radicals. This may be accomplished through metal chelation, or repairing primary antioxidants, among other mechanisms [5]. The secondary antioxidant activity of a species in the presence of redox metal ions is related to their complexation of these ions and the effect that the resulting complexes can have in the inactivation of ^•^OH radicals. This can be achieved in two ways, either by slowing down the reduction of these metal ions upon complexation, which reduces, and sometimes fully inhibits, the formation of ^•^OH radicals via Fenton-like reactions, or by the scavenging of the ^•^OH radicals formed this way when they react with the coordination compound formed with the metal ion. In this second approach, the ligand acts as a sacrifice target preventing ^•^OH radicals from reacting with more important biomolecules such as lipids, proteins and DNA [6,7,8]. Theoretical approaches allow the kinetic study of various type of antioxidants [9,10,11,12,13,14,15].

The reduction of Fe(III) or Cu(II) can lead to the formation of very reactive ^•^OH radicals through a sequence of reactions known as the Haber–Weiss cycle [16,17,18]. The superoxide radical anion or ascorbate can take part in this sequence of reactions. The Haber–Weiss reactions for Fe(III) reacting with $O_{2}^{•-}$ are shown in Equation (1). The second step of this mechanism, known as the Fenton reaction, leads to the formation of ^•^OH radicals. Species that coordinate to Fe(III) or Cu(II) and significantly decrease the rate constant of their reduction to Fe(II) or Cu(I), respectively, are said to have secondary antioxidant activity. Sometimes, these species can fully inhibit the formation of ^•^OH radicals and reduce the potential damage these radicals can cause.

(1) $${\mathrm{Fe}}^{3+}+O_{2}^{•-}\to {\;\mathrm{Fe}}^{2+}+O_{2}$$

$$\;{\mathrm{Fe}}^{2+}+H_{2}O_{2}\to {\mathrm{Fe}}^{3+}+{\;\mathrm{OH}}^{-}+{}^{•}\mathrm{OH}$$

**Figure 1:**
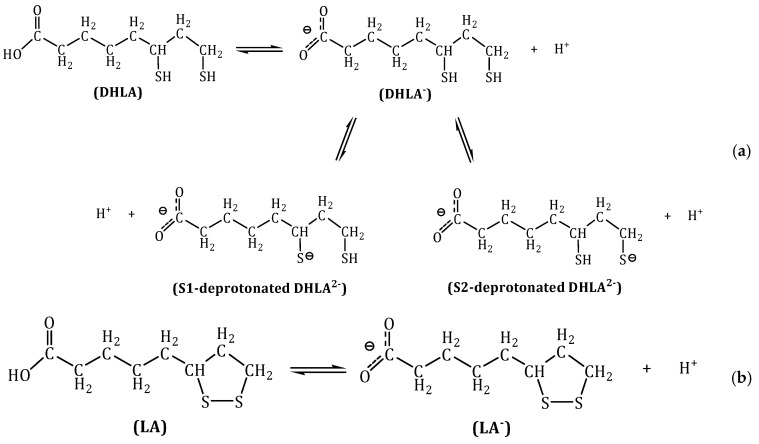
Acid-base equilibria involving dihydrolipoic (DHLA, (**a**)) and lipoic (LA, (**b**)) acids.

Lipoic acid (LA) is an eight-carbon compound, which is rapidly reduced in cells to dihydrolipoic acid (DHLA) [19]. Under physiological conditions (pH = 7.40) these two species are deprotonated. The equilibria between the protonated and deprotonated species are shown in Figure 1. In vitro, LA and DHLA have been shown to have antioxidant activity [20], by scavenging several free radical species [21] and interacting with other antioxidant species.

Ascorbic acid, also known as Vitamin C, exists as two enantiomers, but only the L enantiomer, (5*R*)-[(1*S*)-1,2-Dihydroxyethyl]-3,4-dihydroxyfuran-2(5H)-one, has a significant biological role with two optically active centres. Since the p*K*_a_ of ascorbic acid is 4.70 [22], it is deprotonated under physiological conditions. The structure of ascorbate (${\mathrm{ASC}}^{-}$) is labelled {48} in the Supporting Information.

Previous research carried out by Sigel et al. [23,24] states that the basicity of the carboxyl group in LA complexes is related to their stability. They suggested that when LA coordinates with one of the sulphur atoms, stability is increased. Bonomi et al. showed evidence that the DHLA complex with Fe(III) is more stable than with Fe(II) [25].

Castañeda-Arriaga et al. [10] theoretically investigated the thermodynamic stability of twenty-eight 1:1 Cu(II) complexes with LA and DHLA at the M06-2X(SMD)/6-31++G(d,p) level of theory in water under physiological pH conditions at 298.15 K. The most thermodynamically stable complex was found to slow down the first step of the Haber–Weiss cycle by two orders through reaction with $O_{2}^{•-}$. The primary antioxidant activity of DHLA has also been previously investigated at the M06-2X(SMD)/6-31++G(d,p) level of theory with respect to its ability to repair a damaged protein [13,14,15].

In this study, the thermodynamic stability of twenty-nine 1:1 Fe(III) complexes with various forms of LA and DHLA is examined at the M06(SMD)/6-31++G(d,p) level of theory in water under physiological pH conditions at 298.15 K. The secondary antioxidant activity of LA and DHLA, in terms of their potential to inhibit the formation of ^•^OH radicals, is evaluated with respect to the Fe(III)/Fe(II) reduction by $O_{2}^{•-}$ and ascorbate. Some Cu(II)/Cu(I) calculations are performed with the M06 functional to allow comparison with the Fe(III)/Fe(II) calculations. While a few theoretical studies have evaluated the secondary antioxidant activity (with rate constant calculations) of various organic ligands with respect to the Cu(II)/Cu(I) reduction with $O_{2}^{•-}$ and ascorbate, [10,11,26,27,28] to the best of our knowledge, theoretical kinetic studies of secondary antioxidant activity relative to the Fe(III)/Fe(II) reduction, and their comparison with the Cu(II)/Cu(I) redox system, have not been previously reported for any antioxidant. However, this is a topic of relevance that has been studied experimentally for a variety of substrates [29,30,31,32,33].

## 2. Computational Details

Theoretical calculations were performed using the Gaussian 09 software package [34]. Geometry optimizations, frequency and single-point calculations were performed using the M06 functional [35] and the 6-31++G(d,p) basis set. The M06 functional is the recommended choice over M06-2X when doing calculations with transition metals. The ultrafine integration grid was used in all calculations, which were carried out in water using the SMD continuum solvation model. The SMD model, which is based on the quantum mechanical charge density of the solute interacting with a continuum description of the solvent, is considered a universal solvation model, due to its applicability to any charged or uncharged solute in any solvent or liquid medium for which a few key descriptors are known [36]. The M06 and M06-2X functionals (or their previous versions, the M05 and M05-2X functionals) combined with the SMD continuum solvation model have been successfully used in various kinetic studies in solution [10,11,12,13,14,15,25,26,27,28].

The standard Gibbs free energy change ($∆G_{f}^{{}^{\circ}}$) for the reaction of forming a complex from its infinitely-separated ligands and solvated central ion was calculated using Equation (2) and the corresponding absolute standard Gibbs free energy values (*G*^0^) of reactants and products. The formation constant ($K_{f}$) of each complex was calculated at 298.15 K using Equation (3).

(2) $$∆G_{f}^{{}^{\circ}}=\sum G_{\mathrm{products}}^{{}^{\circ}}-\sum G_{\mathrm{reactants}}^{{}^{\circ}}$$

(3) $$\;K_{f}=e^{-\frac{∆G_{f}^{{}^{\circ}}}{\mathrm{RT}}}\;$$

Rate constants (*k*) for the single electron-transfer reactions were calculated applying conventional transition state theory [37,38]. The standard Gibbs free energies of activation were estimated using Marcus theory [39,40]. For rate constants in the diffuse-limited regime (*k* > 1.0 × 10^8^ M^−^^1^ s^−^^1^), the Collins–Kimball theory [41] was applied to determine apparent rate constants (*k*_app_) in combination with the steady-state Smoluchowski rate constant expression for an irreversible diffusion-controlled bimolecular reaction [42], and the Stokes–Einstein approach for the diffusion coefficients [43,44]. More detailed information on these expressions and their application in various studies can be found elsewhere [10,11,12,13,14,15,45].

## 3. Results and Discussion

At physiological pH (7.40), LA and DHLA are mostly deprotonated (shown as LA^−^, DHLA^−^) because their p*K*_a_ values in aqueous solution at 298.15 K are 4.76 and 4.85, respectively [46]. Consequently, these species can coordinate through one of the carbonyl oxygen atoms (CO) or with both oxygen atoms (COO) of the carboxylate group, as well as with one or both sulfur atoms (S1, S2). In the discussion that follows, only 1:1 complexes have been considered (complexes in which only one organic ligand is present) and numeric labels are used to identify each complex. Iron complexes in each group have been labelled in sequence starting with the most stable one. The coordination sites of the organic ligand are explicitly indicated, together with their geometric distribution (*cis* or *trans*) when applicable. Each complex formation equilibrium considered has as many species in the reactant and product side so that reference state conversions or other corrections are not necessary.

The M06(SMD)/6-31++G(d,p) Cartesian coordinates, as well as the absolute enthalpies and Gibbs free energies of the different species considered in this study in water at 298.15 K are reported in the Supporting Information (Table S1). Fe(III) complexes tend to be octahedral (hexa-coordinated), but tetra- and penta-coordinated complexes are also possible [47], and they are explored in Section 3.2. Test calculations were performed for low- and high-spin complexes. The latter ones were significantly more stable in all cases, which shows that the ligands studied (LA^−^, DHLA^−^ and DHLA^2−^) are weak-field ligands. Some of these test results are shown in Table S2. The iron complexes discussed in the sections that follow are high spin.

### 3.1. Octahedral Complexes with Fe(III)

**Figure 2:**
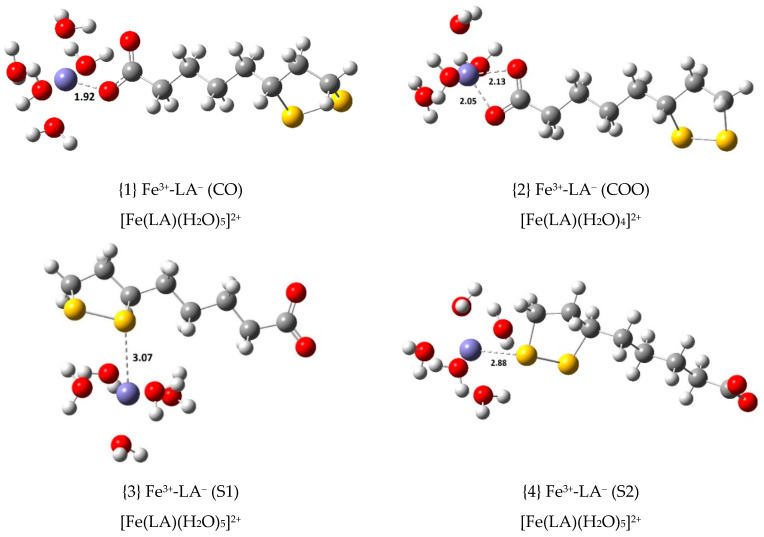
Optimized geometries of the 1:1 complexes of Fe(III) with lipoic acid, LA^−^ (bond distances in Å).

Four Fe(III) complexes with LA^−^ were calculated. Their $∆G_{f}^{{}^{\circ}}$, $K_{f}$ and $\log K_{f}$ values are displayed in Table 1, following the equilibrium indicated in Equation (4), and their structures are shown in Figure 2. In this equilibrium, and in others that will be presented in the following sections, (6 − n)H_2_O identifies an optimized cluster of no more than five water molecules. In the present study, clusters with up to five water molecules were calculated.

Two of the four Fe(III) complexes with LA^−^, {1} and {2}, were found to be exergonic, with $∆G_{f}^{{}^{\circ}}$ values of −18.1 and −16.4 kcal/mol, respectively. The most stable complex has one of the carboxylate oxygen atoms coordinating, while complex {2} has both oxygen atoms coordinating the central ion. Further, the formation constant of complex {2} is eighteen times less than that of complex {1}. When coordination occurs via the sulfur atoms of LA^−^ (or DHLA^−^), thermodynamic stability is greatly reduced. This was also observed when studying LA^−^ and DHLA^−^ coordination to Cu(II) [10]. Coordination bond distances between Fe(III) and LA^−^ are always slightly larger than their Cu(II) counterparts with similar coordination patterns, in agreement with a larger atomic radius for Fe(III) (78.5 pm when 6-coordinated, octahedral, high spin) relative to that of Cu(II) (71 pm, when square planar). Optimizations of Fe(III) complexes with simultaneous oxygen and sulfur coordination from LA^−^, previously reported for Cu(II), were unsuccessful; the sulfur coordination is lost in every attempt.

LA^−^ + [Fe(H_2_O)_6_]^3+^ ⇆ [Fe(LA)(H_2_O)_n_]^2+^ + (6 − n)H_2_O (4)

$$∆G_{f}^{{}^{\circ}}{}_{{\mathrm{Fe}}^{3+}-{\mathrm{LA}}^{-}},\;K_{f_{{\mathrm{Fe}}^{3+}-{\mathrm{LA}}^{-}}}$$

**Figure 3:**
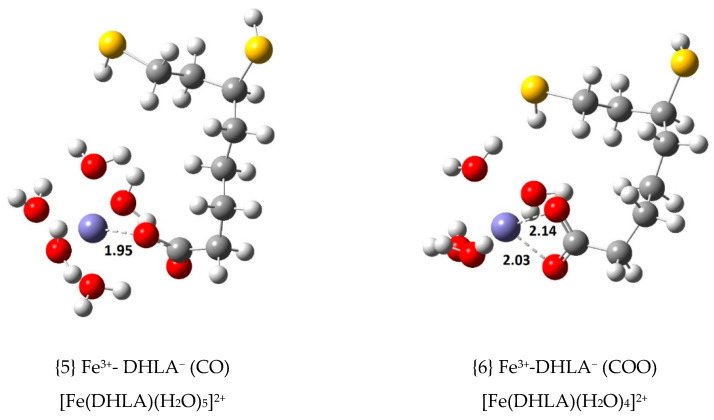

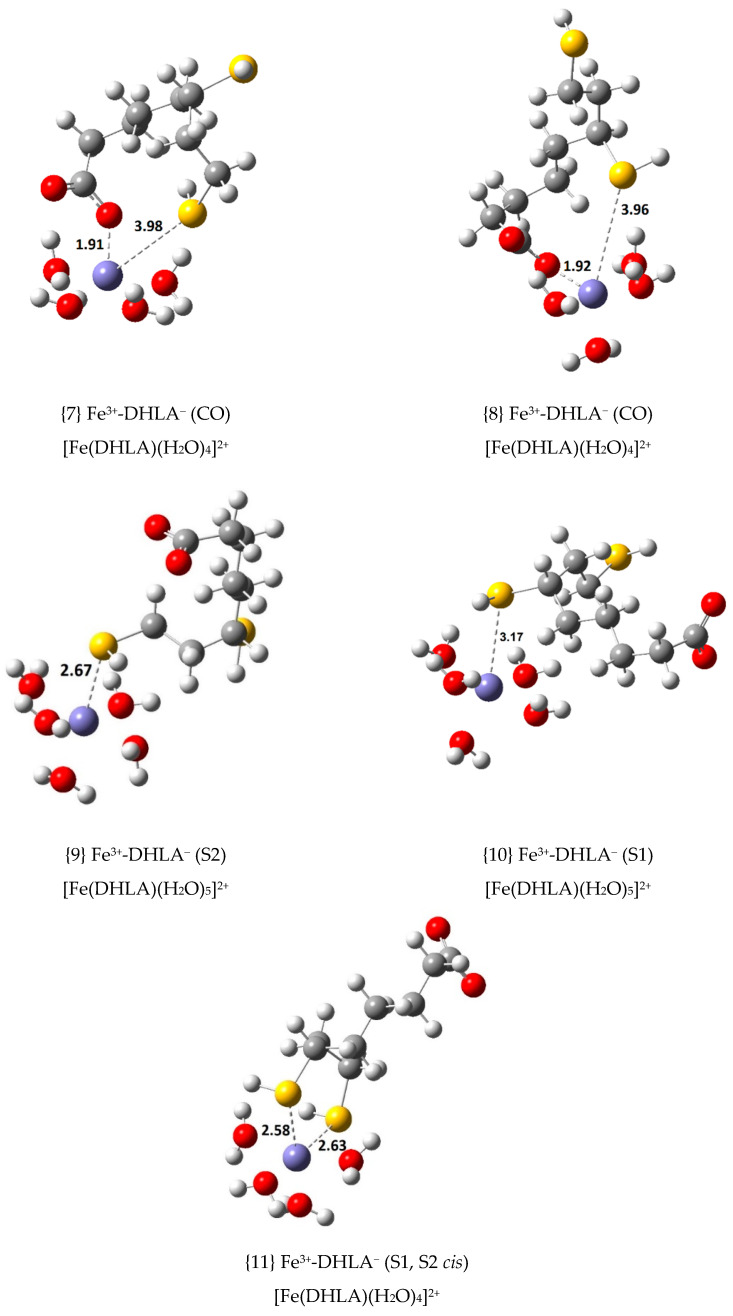
Optimized geometries of the 1:1 complexes of Fe(III) with dihydrolipoic acid, DHLA^−^ (bond distances in Å).

Seven Fe(III) complexes with DHLA^−^ were optimized. Their structures, with relevant bond distances indicated, are displayed in Figure 3. Table 2 contains their $∆G_{f}^{{}^{\circ}}$, $K_{f}$ and $\log\;K_{f}$ values as per the complex formation equilibrium shown in Equation (5). Four of these complexes are exergonic. The two most stable complexes also have DHLA^−^ coordinating via CO (monodentate), {5}, and COO (bidentate), {6}. These are 2–3 kcal/mol less stable than their LA^-^ counterparts, complexes {1} and {2}, respectively. However, their coordinating bond distances to Fe(III) are very similar to the ones found with LA^−^ (see Figure 2). Similar observations can be made on these last two points when inspecting the Cu(II) complexes with LA^−^ and DHLA^−^ [10]. The other two exergonic Fe(III) complexes with DHLA^−^, {7} and {8}, seem to be penta-coordinated. Both contain CO coordination from DHLA^−^ and each of the sulfur atoms are nearby, but too far away (3.96–3.98 Å) from the central ion to coordinate to it. These complexes are 1.3–2.0 kcal/mol less stable than the equivalent octahedral complex {5}. Coordination via S1 and S2 led to complex {11}, which is less stable than when only S2 or S1 coordinates to the central ion, {9} and {10}. These three complexes, {9}, {10} and {11} are endergonic. Complex {11}, the least stable of all, seems to be too strained and entropically unfavourable; it exhibits the shortest Fe(III)-S coordinating distances (2.58 and 2.63 Å) when compared to other LA^−^ and DHLA^−^ complexes.

DHLA^−^ + [Fe(H_2_O)_6_]^3+^ ⇆ [Fe(DHLA)(H_2_O)_n_]^2+^ + (6 − n)H_2_O (5)

$$∆G_{f}^{{}^{\circ}}{}_{{\mathrm{Fe}}^{3+}-{\mathrm{DHLA}}^{-}},\;K_{f_{{\mathrm{Fe}}^{3+}-{\mathrm{DHLA}}^{-}}}$$

**Figure 4:**
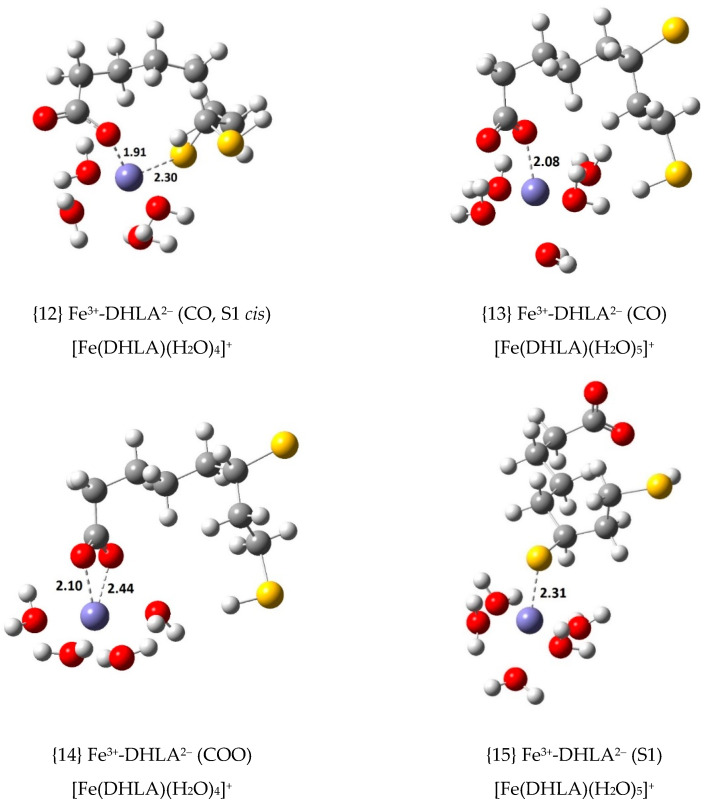
Optimized geometries of the 1:1 complexes of Fe(III) with S1-deprotonated DHLA^2−^ (bond distances in Å).

**Figure 5:**
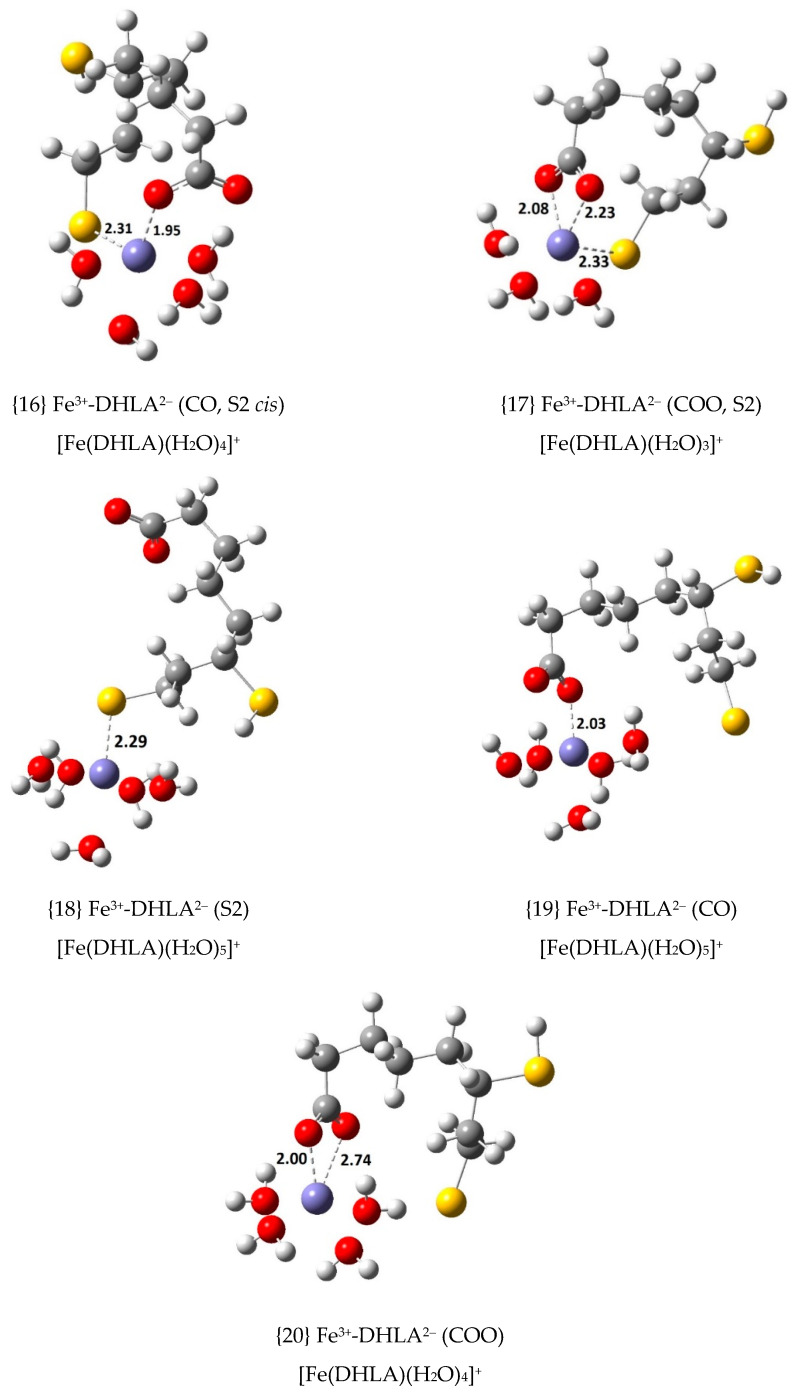
Optimized geometries of the 1:1 complexes of Fe(III) with S2-deprotonated DHLA^2−^ (bond distances in Å).

To further explore other possible Fe(III) complexes, the doubly deprotonated DHLA anion, DHLA^2^^−^, was also considered as a ligand with a second deprotonation in the thiol group closer to the carboxylate group (S1) and in the thiol group which is farther apart (S2) (see Figure 1). Four Fe(III) complexes were calculated with S1-deprotonated DHLA^2−^ (see Figure 4) and five with the S2-deprotonated anion (see Figure 5). Thermodynamic calculations were performed to determine their $∆G_{f}^{{}^{\circ}}$, $K_{f}$ and $\log\;K_{f}$ values as shown in Table S3, according to the formation equilibrium shown in Equation (6).

DHLA^2−^ + [Fe(H_2_O)_6_]^3+^ ⇆ [Fe(DHLA)(H_2_O)_n_]^+^ + (6 − n)H_2_O (6)

$$∆G_{f}^{{}^{\circ}}{}_{{\mathrm{Fe}}^{3+}-{\mathrm{DHLA}}^{2-}},\;K_{f_{{\mathrm{Fe}}^{3+}-{\mathrm{DHLA}}^{2-}}}$$

As previously illustrated in our study of the Cu(II) complexes with DHLA^2−^ [10], transforming Equation (6) into Equation (7) implies adding 4.50 kcal/mol to the $∆G_{f}^{{}^{\circ}}$ values reported in Table S3. This value, 4.50 kcal/mol, is the thermodynamic cost of forming DHLA^2−^ in water under physiological pH conditions. The new thermodynamic results, displayed in Table 3, can be properly compared to those related to the formation equilibria shown in Equations (4) and (5).

DHLA^−^ + [Fe(H_2_O)_6_]^3+^ ⇆ [Fe(DHLA)(H_2_O)_n_]^+^ + (6 − n)H_2_O + H^+^ (7)

$$\;∆G_{f}^{{}^{\circ}}{{}^{\prime }}_{{\mathrm{Fe}}^{3+}-{\mathrm{DHLA}}^{2-}},\;K_{f}{{}^{\prime }}_{{\mathrm{Fe}}^{3+}-{\mathrm{DHLA}}^{2-}}\;$$

It can be observed that relative to the new set of reactant species (DHLA^−^ and [Fe(H_2_O)_6_]^3+^), the nine complexes with DHLA^2^**^−^** are significantly exergonic, with $∆G_{f}^{{}^{\circ}}$ values from −21.9 to −37.3 kcal/mol. They are much more thermodynamically stable than the previously studied complexes with LA^−^ and DHLA^−^ as ligands. A similar situation was observed in the study of the Cu(II) complexes with the M06-2X functional; however, the complexes with DHLA^2−^ had smaller $∆G_{f}^{{}^{\circ}}$ values, ranging from –3.6 to –21.8 kcal/mol [10].

The most stable complex with DHLA^2−^ in each group involves coordination through an oxygen atom of the carboxylate group and the deprotonated sulphur atom in *cis* configuration. That is the case of complex {12}, with $∆G_{f}^{{}^{\circ}}$ = –30.6 kcal/mol, and {16}, with $∆G_{f}^{{}^{\circ}}$ = –37.3 kcal/mol. Considering all the complexes with DHLA^2−^ in which the deprotonated sulphur atom is a coordination centre, the sulphur-central ion distance (which goes from 2.29 to 2.33 Å) is much shorter than for any complex with LA^−^ and DHLA^−^, which is reasonable given its negative charge and much greater basicity. The two most stable Fe(III) complexes involve S2-deprotonated DHLA^2−^, {16} and {17}, which are followed by two complexes with S1-deprotonated DHLA^2−^, {12} and {13}. Complex {17} is the only one in which the organic ligand studied coordinates to Fe(III) through three atomic centres, the carboxylate group and the deprotonated sulphur atom (S2).

### 3.2. Tetra- and Penta-Coordinated Fe(III) Complexes

**Figure 6:**
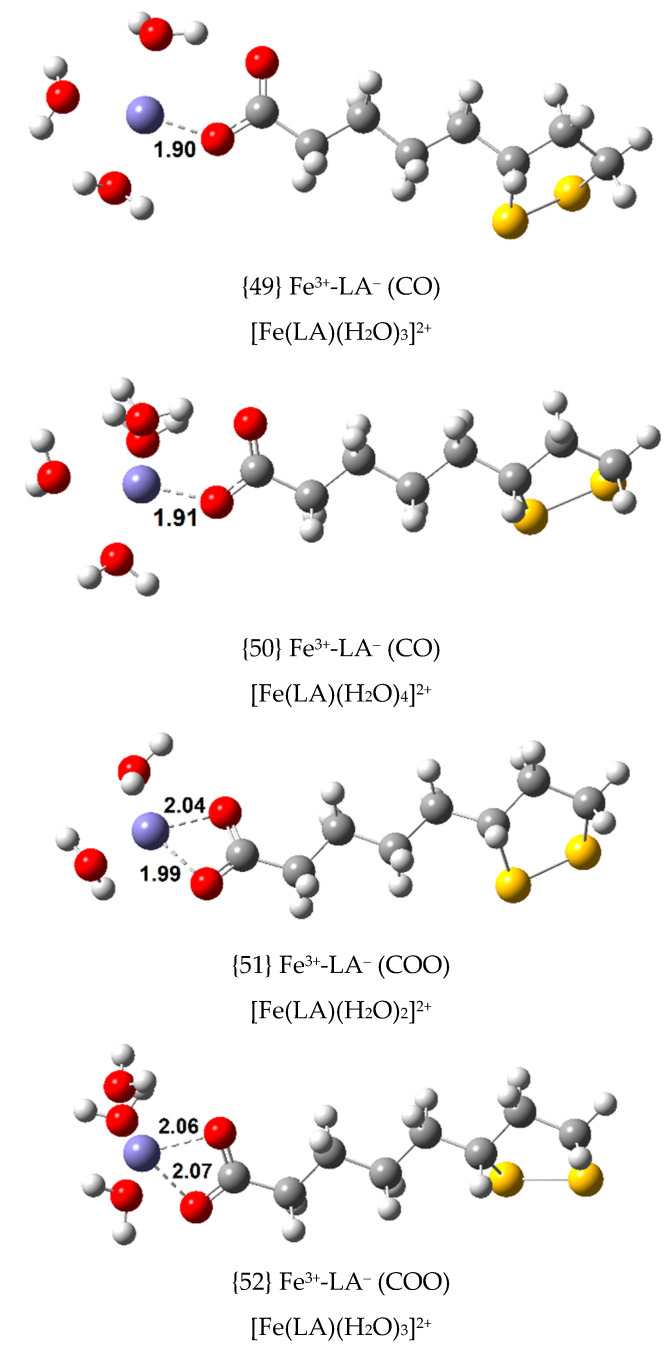
Optimized geometries of non-octahedral 1:1 complexes of Fe(III) with LA^−^ (bond distances in Å).

**Figure 7:**
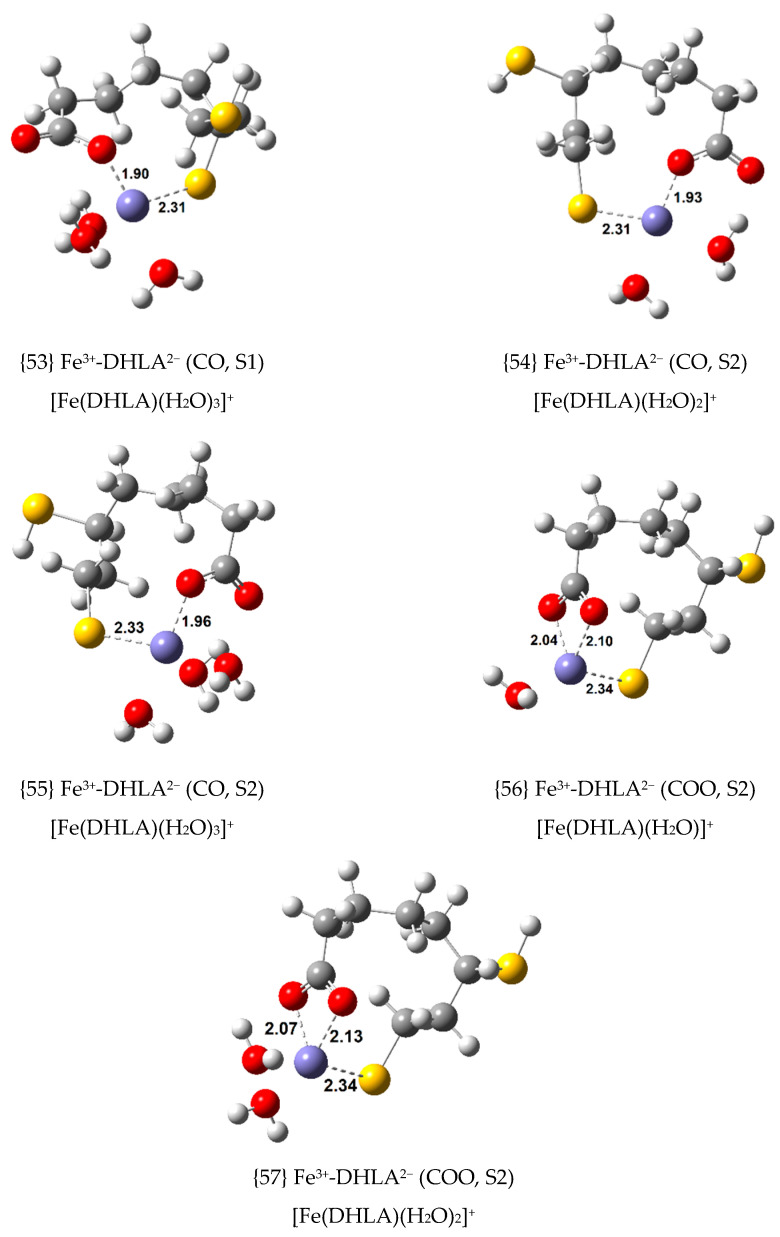
Optimized geometries of non-octahedral 1:1 complexes of Fe(III) with S1- and S2-deprotonated DHLA^2−^ (bond distances in Å).

Since Fe(III) is also known to form non-octahedral complexes, tetra- and penta-coordinated complexes were optimized from the most stable octahedral complexes with LA^−^ and DHLA^2^**^−^**, complexes {1}, {2}, {12}, {16} and {17}. Nine new exergonic Fe(III) complexes were optimized. Their $∆G_{f}^{{}^{\circ}}$, $K_{f}^{\;}$ and $\log\;K_{f}^{\;}$ values are displayed in Table 4, and their structures are shown in Figure 6 and Figure 7.

The greater the coordination number of Fe(III), the greater the stability of its complex. Hence, the thermodynamic stability order is: hexa-coordinated (octahedral) > penta-coordinated > tetra-coordinated. Complexes {55} and {54} with S2-deprotonated DHLA^2−^ as a ligand, closely related to the most stable complex {16}, with $∆G_{f}^{{}^{\circ}}$ of –37.3 kcal/mol, are the two most stable complexes in this group with $∆G_{f}^{{}^{\circ}}$ values of –36.3 and –30.1 kcal/mol, respectively.

### 3.3. Reduction of Fe(III) to Fe(II): The First Step of the Haber–Weiss Cycle

When Fe(III) is reduced by a strong reducing agent (the superoxide radical anion, $O_{2}^{•-}$, or ascorbate, ${\mathrm{ASC}}^{-}$) to Fe(II), very reactive (and harmful) hydroxyl radicals are formed as a product of the Haber–Weiss cycle. In the first step of the cycle, Fe(III) is reduced to Fe(II). In the next step, known as the Fenton reaction, Fe(II) is oxidized back to Fe(III) and ^•^OH radicals are formed, as shown in Equations (8) and (9).

(8) $${\mathrm{Fe}}^{3+}+{\mathrm{ASC}}^{-}\to {\;\mathrm{Fe}}^{2+}+{\mathrm{ASC}}^{•}$$

(9) $$\;{\mathrm{Fe}}^{2+}+H_{2}O_{2}\to {\mathrm{Fe}}^{3+}+{\;\mathrm{OH}}^{-}+{}^{•}\mathrm{OH}$$

**Figure 8:**
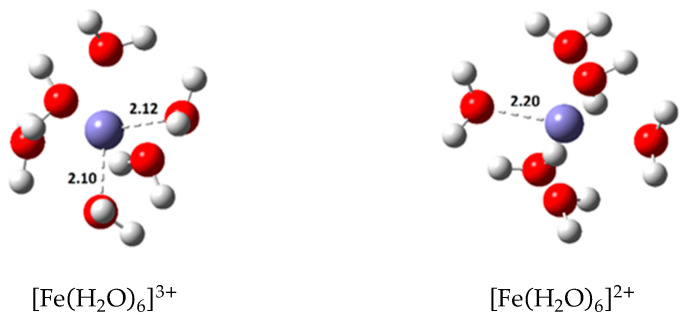
Optimized geometries of the most stable hydrated Fe(III) and Fe(II) complexes (bond distances in Å).

Since the ions Fe(III) and Fe(II)  are solvated in water, a hydrated octahedral complex is a more realistic representation of these species. The most stable hydrated complexes calculated are shown in Figure 8. Therefore, the actual reference reactions considered for the kinetic study of the first step of the Haber–Weiss cycle are shown in Equations (10) and (11).

(10) $${\left[\mathrm{Fe}{\left(H_{2}O\right)}_{6}\right]}^{3+}+{\;O}_{2}^{•-}\to {\left[\mathrm{Fe}{\left(H_{2}O\right)}_{6}\right]}^{2+}+O_{2}$$

(11) $$\;{\left[\mathrm{Fe}{\left(H_{2}O\right)}_{6}\right]}^{3+}+{\;\mathrm{ASC}}^{-}\to {\left[\mathrm{Fe}{\left(H_{2}O\right)}_{6}\right]}^{2+}+{\mathrm{ASC}}^{•}\;$$

Investigating the secondary antioxidant capacity of LA and DHLA relative to the Fe(III)/Fe(II) reduction involves finding out if any of the possible complexes with Fe(III) could significantly slow down the reduction of this ion when in the presence of $O_{2}^{•-}$ or ${\mathrm{ASC}}^{-}$, as represented by Equations (12) and (13) that focus on DHLA^2−^ as organic ligand. If this occurs, the potential damage caused by ^•^OH radical formation could be greatly reduced.

(12) $${\left[\mathrm{FeDHLA}{\left(H_{2}O\right)}_{n}\right]}^{+}+O_{2}^{•-}\to {\left[\mathrm{FeDHLA}{\left(H_{2}O\right)}_{n}\right]}^{\;}+O_{2}$$

(13) $$\;{\left[\mathrm{FeDHLA}{\left(H_{2}O\right)}_{n}\right]}^{+}+{\mathrm{ASC}}^{-}\to {\left[\mathrm{FeDHLA}{\left(H_{2}O\right)}_{n}\right]}^{\;}+{\mathrm{ASC}}^{•}\;$$

**Figure 9:**
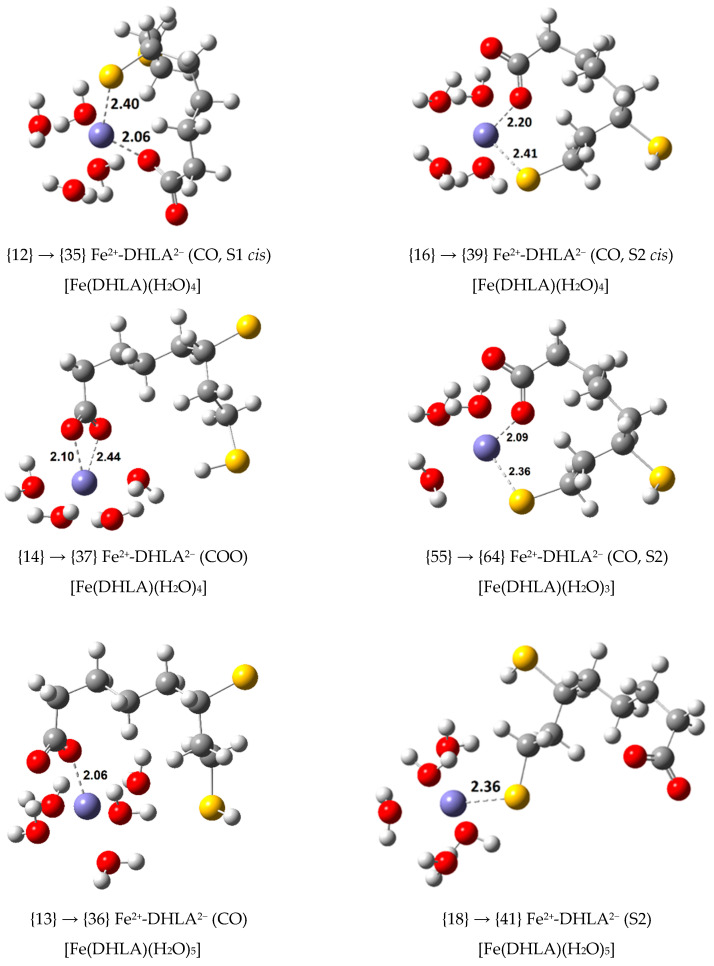
Optimized geometry (bond distances in Å) of the Fe(II) complexes that participate in the redox reactions reported in Table 5.

Hence, with initial focus on the exergonic Fe(III) complexes previously calculated (twenty-four of them), the rate constants of their reduction to Fe(II) by reaction with $O_{2}^{•-}$ and with ${\mathrm{ASC}}^{-}$ were calculated. This required the optimization of the corresponding Fe(II) complex from each Fe(III) complex. The structures of six of these Fe(II) complexes are shown in Figure 9 with a clear indication of the Fe(III) complex related to them. The Fe(II) complexes calculated are high spin (see Table S2) and preserve the coordination number of the corresponding Fe(III) complex, with the exception of {40} that is penta-coordinated but it is the reduction product of the octahedral complex {17}, and {66} that is tetra-coordinated and comes from the penta-coordinated Fe(III) complex {57}. In all cases, the coordinating bond distances between the organic ligand and the central ion, Fe(II), became slightly larger relative to the same distances in the initial Fe(III) complexes, with the exception of complex {66}, for which the CO-Fe and S-Fe distances were slightly decreased.

Standard Gibbs free energies of reaction (Δ*G*°, kcal/mol) and activation (Δ*G*^≠^, kcal/mol), as well as various rate constants (*k*, *k*_D_ and *k*_app_, M^–1^ s^–1^) for the single-electron transfer (SET) reactions with $O_{2}^{•-}$ and with ascorbate are shown in Tables S4 and S5, respectively. Reactions in each table have been listed in ascending order of their calculated apparent rate constant (*k*_app_). In general, the more exergonic the SET reaction, the smaller the calculated Δ*G*^≠^ value and the larger the rate constant. Plots of *k*_app_ *versus* Δ*G*°, which are displayed in Figure S1 for both sets of reactions, show this tendency.

The calculated k values for the reactions with $O_{2}^{•-}$, including that of the reference reaction involving the hydrated ions, are larger than 1.0 × 10^8^ M^−1^ s^−1^ and required diffusion corrections. The calculated *k*_app_ values of these reaction, which are exergonic, are in in the order of 10^9^ M^−1^ s^−1^. Only the *k*_app_ for the {12} → {35} reaction (7.08 × 10^9^ M^−1^ s^−1^) is marginally smaller than that of the reference reaction (7.71 × 10^9^ M^−1^ s^−1^). This shows that relative to the Fe(III)/Fe(II) reduction with $O_{2}^{•-}$, LA and DHLA do not possess secondary antioxidant activity.

Focusing on the reactions with ascorbate, very different results are observed. Reduced kinetic information for the six SET reactions with ascorbate having the smallest rate constants (those from complexes {12}, {16}, {14}, {55}, {13} and {18}, with DHLA^2−^ as ligand) is displayed in Table 5, together with the kinetic information for the reaction of these Fe(III) complexes with $O_{2}^{•-}$. The structures of the Fe(II) complexes involved in these reactions are those shown in Figure 9. The Δ*G*° and Δ*G*^≠^ values of these reaction (see Table S5) are very similar.

The first thirteen of the twenty-four reactions with ascorbate listed in Table S5 are endergonic and show *k* values between 70.8 and 1.16 × 10^8^ M^-1^ s^-1^, which are significantly smaller relative to the reference reaction with *k*_app_ of 7.36 × 10^9^ M^–1^ s^–1^. The eleven most thermodynamically stable Fe(III) complexes (in order: {16} > {55} > {17} > {12} > {54} > {13} > {53} > {57} > {14} > {18} > {19}) are included in this group of reactions, but the $∆G_{f}^{{}^{\circ}}$ values of the Fe(III) complexes and the rate constant of their SET reactions are not 100% correlated. When the *k*_app_ values of the reactions with $O_{2}^{•-}$ and with ascorbate are plotted versus the $∆G_{f}^{{}^{\circ}}$ of the Fe(III) complexes involved, a slight correlation can be observed (see Figure S2). More stable Fe(III) complexes tend to have smaller SET rate constants, but complex {12}, the fourth most stable complex, breaks this general tendency with both reactions. The remaining eleven reactions are slightly exergonic and their *k* values, being larger than 1.0 × 10^9^ M^−^^1^ s^−^^1^, required diffusion corrections and lead to *k*_app_ values from 2.41 × 10^9^ to 7.47 × 10^9^ M^−^^1^ s^−^^1^. All these reactions are slower than the reference reaction except for the reaction from complex {49}.

Hence, relative to the Fe(III)/Fe(II) reduction with ascorbate, DHLA shows significant secondary antioxidant activity to the point of being able to fully inhibit the formation of ^•^OH radicals in the Fenton reaction. The most stable Fe(III) complex, {16}, displays a SET rate constant that is 3.6 × 10^6^ times smaller than the reference reaction. However, LA (see the kinetic results for the reactions from complexes {1} and {2} in Table S5) displays some (minor) degree of secondary antioxidant activity being able to slow the first step of the Haber–Weiss cycle up to three times.

### 3.4. Copper (II) Complexes and Their Comparison with Fe(III) Complexes

**Figure 10:**
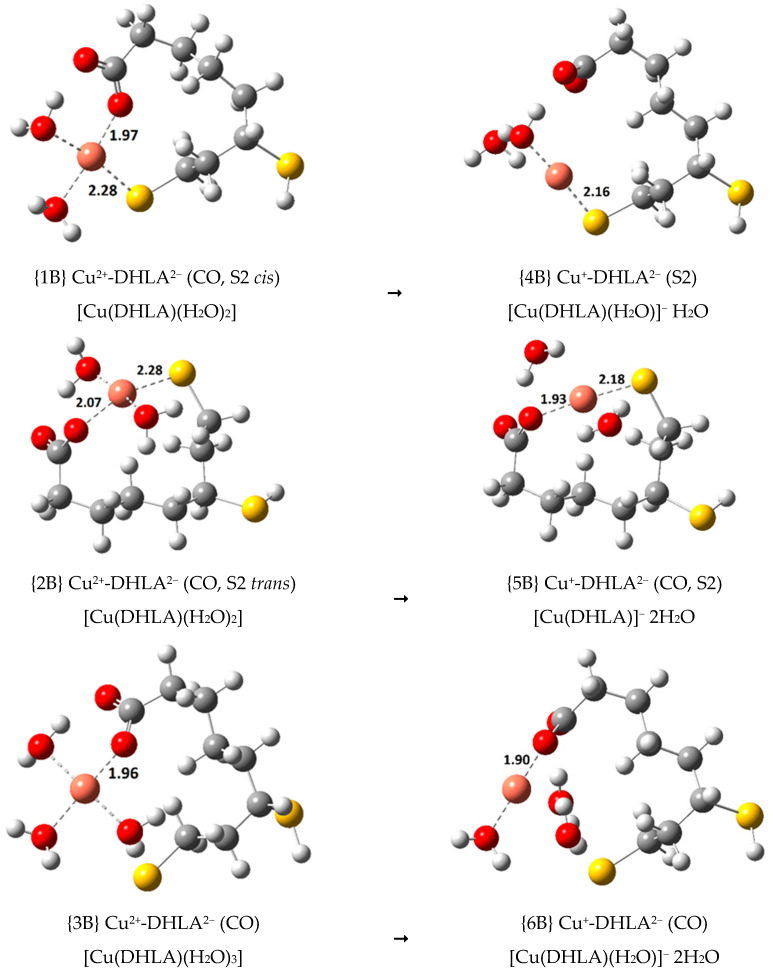
M06 optimized structures (bond distances in Å) of the three most stable Cu(II) complexes ({1B}, {2B}, {3B}), reported in Ref. 10 at the M06-2X level of theory, together with the optimized geometries of the corresponding Cu(I) complexes ({4B}, {5B}, {6B}).

To compare the secondary antioxidant activity of LA and DHLA with respect to the Cu(II)/Cu(I) and Fe(III)/Fe(II) reductions, some of the calculations previously reported in Reference 10 for the Cu(II) complexes at the M06-2X(SMD)/6-31++G(d,p) level of theory have been re-done using the M06 functional. The three most stable Cu(II) complexes (previously reported as complexes {23}, {24} and {25} in Reference 10; now labelled {1B}, {2B} and {3B}), all with the S2-deprotonated DHLA^2^^−^ ligand, were re-optimized and their corresponding linear Cu(I) complexes (labelled {4B}, {5B} and {6B}) were calculated. Reference 10 only reports the Cu(I) complex related to complex {1B}. The new M06 structures are shown in Figure 10.

The most stable Cu(II)-LA^-^ complex (reported as {1} in Reference 10; now labelled {9B}) was also recalculated, and its Cu(I) reduction product was optimized, {10B}. These structures are reported in the Supporting Information. The M06 coordination distances from the Cu(II) central ion to the organic ligand are slightly shorter than in the M06-2X study in most cases, and they become shorter in the corresponding Cu(I) complex. Thermodynamic calculations related to the formation of the Cu(II)-DHLA^2^^−^ complexes at physiological pH, as shown in Equation (14), are reported in Table 6.

DHLA^–^ + [Cu(H_2_O)_4_]^2+^ ⇆ [Cu(DHLA)(H_2_O)_n_] + (4 − n)H_2_O + H^+^ (14)

The relative thermodynamic stability order of complexes {1B}, {2B} and {3B}, previously reported with (M06-2X) $∆G_{f}^{{}^{\circ}}$ values of −21.8, −18.26 and −18.0 kcal/mol (see Table 6 of Reference 10) respectively, is preserved with new (M06) values of −31.4, −30.0 and −18.6 kcal/mol, respectively, that are significantly more negative for {1B} and {2B}. These complexes are predicted to be more stable with the M06 functional than with the M06-2X functional, with $∆G_{f}^{{}^{\circ}}$ values in line with those obtained for the Fe(III) complexes with similar coordination patterns. For example, {16} Fe^3+^-DHLA^2−^ (CO, S2 *cis*), the most stable Fe(III) complex calculated in the present study with $∆G_{f}^{{}^{\circ}}$ of −37.3 kcal/mol, compares to the most stable Cu(II) complex, {1B} Cu^2+^-DHLA^2−^ (CO, S2 *cis*), with $∆G_{f}^{{}^{\circ}}$ of −31.4 kcal/mol. Complex {2B} has no equivalent Fe(III) complex, but {3B}, with $∆G_{f}^{{}^{\circ}}$ of −18.6 kcal/mol, is once again about 6 kcal/mol less stable that its Fe(III) counterpart, {19} Fe^3+^-DHLA^2−^ (CO), with $∆G_{f}^{{}^{\circ}}$ of −24.5 kcal/mol. These results seem to indicate that the Fe(III) complexes with DHLA^2−^ are more thermodynamically stable than the Cu(II) complexes.

The same is observed when comparing the M06-2X ($∆G_{f}^{{}^{\circ}}$ = −13.1 kcal/mol) and M06 ($∆G_{f}^{{}^{\circ}}$ = −13.4 kcal/mol) stability of the most stable Cu(II)-LA^−^ complex, which is also more stable than the Cu(II)-DHLA^−^ complexes. Similarly, the most stable Fe(III)-LA^-^ complex ($∆G_{f}^{{}^{\circ}}$ = −18.1 kcal/mol) is about 4.7 kcal/mol more stable than its Cu(II) counterpart. Hence, the Fe(III) complexes appear to be more thermodynamically stable than their equivalent Cu(II) complexes.

The kinetic study of the SET reactions of {1B}, {2B}, {3B} and {9B} with the superoxide radical anion and ascorbate was also performed. Standard Gibbs free energies of reaction (ΔG°, kcal/mol) and activation (Δ*G*^≠^, kcal/mol), as well as various rate constants (k, k_D_ and k_app_, M^–1^ s^–1^) are shown in Table S6. Reduced kinetic information is displayed in Table 7.

The reactions of the Cu(II) complexes with $O_{2}^{•-}$ lead to *k*_app_ values that are 1.2 to 3.4 times smaller than the *k*_app_ value of the reference reaction (7.43 × 10^9^ M^–1^ s^–1^). The experimentally measured rate constant for the reference reaction, 8.1 × 10^9^ M^–1^ s^–1^, is in excellent agreement with our calculation [48]. The most stable complex, {1B}, leads to the greatest rate constant reduction (2.19 × 10^9^ M^–1^ s^–1^), which is almost non-existent when considering the Fe(III) reactions (see Table 5). This shows that relative to the Cu(II)/Cu(I) reduction with $O_{2}^{•-}$, DHLA and LA possess some (minor) degree of secondary antioxidant activity, which is non-existent relative to the Fe(III)/Fe(II) reduction.

In the previous study [10], the M06-2X rate constant of the reference reaction was calculated to be 1.29 × 10^8^ M^−^^1^ s^−^^1^, and the rate constant for the reduction of the most stable complex, {1B}, with $O_{2}^{•-}$ was almost 100 times less (*k* = 1.33 × 10^6^ M^−^^1^ s^−^^1^). The k values with the M06 functional are larger (6.50 × 10^11^ M^−^^1^ s^−^^1^ for the reference reaction and 3.00 x 10^9^ M^-1^ s^-1^ for the reduction of {1B}, which is slightly over 200 times slower), but the diffusion corrections applied reduce the difference between the final *k*_app_ values obtained, which are both in the order of 10^9^ M^−1^ s^−1^.

Similarly to what was found with the Fe(III) reactions (see Table S5), the reactions of the Cu(II) complexes with ascorbate have smaller rate constants that those of the reactions with $O_{2}^{•-}$ and are significantly slower, by as much as 7.6 × 10^3^ times, than the reference reaction. The rate constant of the reference reaction is 2.51 × 10^7^ M^−^^1^ s^−^^1^, and the rate constant for the reduction of the most stable complex, {1B}, is 3.32 × 10^3^ M^−^^1^ s^−^^1^. This shows that relative to the Cu(II)/Cu(I) reduction with ascorbate, DHLA and LA possess significant secondary antioxidant activity. However, relative to the Fe(III)/Fe(II) reduction (see Table S5), DHLA exhibits a very high secondary antioxidant character (with rate constant reduction of up to 10^8^ times), and LA plays a minor role this way.

## 4. Conclusions

The thermodynamic stability of twenty-nine 1:1 Fe(III) complexes with LA^-^, DHLA^-^ and DHLA^2−^ (in two forms) was studied at the M06(SMD)/6-31++G(d,p) level of theory in water under physiological pH conditions at 298.15 K. Twenty-four exergonic Fe(III) complexes were used to study the kinetics of the SET reaction with $O_{2}^{•-}$ and ascorbate to investigate the secondary antioxidant activity of LA and DHLA relative to their ability to slow down the reduction of these metal ions upon complexation, which reduces and sometimes fully inhibits the formation of ^•^OH radicals via Fenton-like reactions. To the best of our knowledge, this is the first theoretical kinetic study reported on secondary antioxidant activity relative to the Fe(III)/Fe(II) reduction and its comparison to the Cu(II)/Cu(I) reduction for the same set of antioxidants.

Penta- and tetra-coordinated Fe(III) complexes were found to be less stable that the octahedral ones for equivalent coordination of the organic ligand. However, the penta-coordinated complex {55} is only 0.7 kcal/mol less stable than the most stable complex, {16} with a $∆G_{f}^{{}^{\circ}}$ of −37.3 kcal/mol, both with S2-deprotonated DHLA^2−^. The thermodynamic stability of the Fe(III) complexes does not fully correlate with the rate constant of their SET reactions. Only one Fe(III) complex was found to marginally reduce the rate constant of the first step of the Haber–Weiss cycle when reacting with $O_{2}^{•-}$, relative to the reference reaction of the hydrated Fe(III) complex. However, eight of the exergonic Fe(III) complexes (with DHLA^2^^−^) were able to significantly reduce this rate constant from 1.9 × 10^3^ to 1.0 × 10^8^ times when reacting with ascorbate. Minor rate constant reductions with ascorbate were found for the Fe(III) complexes with LA^−^ and DHLA^−^.

Some Cu(II) complexes were calculated for comparison and the kinetics of their reduction with $O_{2}^{•-}$ and ascorbate was studied. Both Cu(II) and Fe(III) can form more stable complexes with LA^−^ than with DHLA^−^ (with similar coordination patterns leading to the most stable complexes with each ligand). However, for both ions the most stable complexes are with S2-deprotonated DHLA^2^^−^, when one or two oxygen atoms of the carboxyl group and/or the deprotonated sulphur atom are coordinated to the central ion. It seems that the Fe(III) complexes are more thermodynamically stable than the Cu(II) complexes with the same ligand and coordination, but their reduction with $O_{2}^{•-}$, relative to the reference reaction, is not slower than with the Cu(II) complexes. However, this is not the case with ascorbate.

Relative to the Fe(III)/Fe(II) reduction with ascorbate, DHLA can fully inhibit the formation of ^•^OH radicals but not by reaction with $O_{2}^{•-}$. Relative to the Cu(II)/Cu(I) reduction with ascorbate, the effects of DHLA are moderate, and with $O_{2}^{•-}$ they are negligible. LA has negligible inhibition effects in all the cases considered. One would expect that some of these complexes could easily scavenge the subsequently formed ^•^OH radical via SET and hydrogen-atom transfer reactions, something that has been shown the hydrated Cu(II) complex cannot do. The coordinated water molecules of Cu(II) complexes with phenolic compounds have been shown to play a role in this mechanism of antioxidant activity [28]. From our calculations, it could be interpreted that if DHLA is present, it will form a complex with Fe(III) with much greater probability than it will with Cu(II), if their concentrations are similar. Hence, the kinetic results we have obtained relative to the Fe(III)/Fe(II) reduction would provide a more realistic picture of its secondary antioxidant activity when its complexes react with $O_{2}^{•-}$ or with ascorbate.

## Figures and Tables

**Table 1:** Standard Gibbs free energy change ($∆G_{f}^{{}^{\circ}}$, in kcal/mol) and formation constant ($K_{f}$ $\log K_{f}$ ) for the chelation of Fe(III) with lipoic acid, LA^−^, (as per Equation (4)) in aqueous solution at 298.15 K. ^a^

Complex ${\left[\mathrm{Fe}\left(\mathrm{LA}\right){\left(H_{2}O\right)}_{n}\right]}^{2+}$	$\;∆G_{f}^{{}^{\circ}}{}_{Fe^{3+}-LA^{-}}$	$\;K_{f_{Fe^{3+}-LA^{-}}}\;$	$\;\log K_{f_{Fe^{3+}-LA^{-}}}\;$
{1} Fe^3+^-LA^−^ (CO)	−18.1	1.85 × 10^13^	13.27
{2} Fe^3+^-LA^−^ (COO)	−16.4	1.01 × 10^12^	12.00
{3} Fe^3+^-LA^−^ (S1)	9.5	9.99 × 10^-8^	−7.00
{4} Fe^3+^-LA^−^ (S2)	11.8	2.39 × 10^-9^	−8.62

^a^ Coordinating atoms in the organic ligand are shown in parentheses for each complex.

**Table 2:** Standard Gibbs free energy change ($∆G_{f}^{{}^{\circ}}$, in kcal/mol) and formation constant ($K_{f}$, $\log K_{f}$ ) for the chelation of Fe(III) with dihydrolipoic acid, DHLA^−^, (as per Equation (5)) in aqueous solution at 298.15 K. ^a^

Complex ${\left[\mathrm{Fe}\left(\mathrm{DHLA}\right){\left(H_{2}O\right)}_{n}\right]}^{2+}$	$\;∆G_{f}^{{}^{\circ}}{}_{{\mathrm{Fe}}^{3+}-{\mathrm{DHLA}}^{-}}$	$\;K_{f_{{\mathrm{Fe}}^{3+}-{\mathrm{DHLA}}^{-}}}\;$	$\;\log K_{f_{{\mathrm{Fe}}^{3+}-{\mathrm{DHLA}}^{-}}}\;$
{5} Fe^3+^-DHLA^−^ (CO)	−15.1	1.11 × 10^11^	11.04
{6} Fe^3+^-DHLA^−^ (COO)	−14.2	2.73 × 10^10^	10.44
{7} Fe^3+^-DHLA^−^ (CO) ^b^	−13.8	1.28 × 10^10^	10.11
{8} Fe^3+^-DHLA^−^ (CO) ^b^	−13.1	3.74 × 10^9^	9.57
{9} Fe^3+^-DHLA^−^ (S2)	16.8	4.64 × 10^-13^	−12.33
{10} Fe^3+^-DHLA^−^ (S1)	17.4	1.69 × 10^-13^	−12.77
{11} Fe^3+^-DHLA^−^ (S1, S2 *cis*)	22.3	4.07 × 10^-17^	−16.39

^a^ Coordinating atoms in the organic ligand are shown in parentheses for each complex. ^b^ Pentacoordinated complexes.

**Table 3:** Standard Gibbs free energy change ($∆G_{f}^{{}^{\circ}}{}^{\prime }$, in kcal/mol) and equilibrium constant ($K_{f}{}^{\prime }$, $\log K_{f}{}^{\prime }$ ) for the chelation of Fe(III) with S1- and S2-deprotonated DHLA^2−^ from DHLA^–^ and the hydrated central ion (as per Equation (7)) in aqueous solution at 298.15 K. ^a^

Complex ${\left[\mathrm{Fe}\left(\mathrm{DHLA}\right){\left(H_{2}O\right)}_{n}\right]}^{+}$	$\;∆G_{f}^{{}^{\circ}}{{}^{\prime }}_{{\mathrm{Fe}}^{3+}-{\mathrm{DHLA}}^{2-}}$	$\;K_{f}{{}^{\prime }}_{{\mathrm{Fe}}^{3+}-{\mathrm{DHLA}}^{2-}}$	$\;\log K_{f}{{}^{\prime }}_{{\mathrm{Fe}}^{3+}-{\mathrm{DHLA}}^{2-}}\;$
S1-deprotonated DHLA^2−^			
{12} Fe^3+^-DHLA^2−^ (CO, S1 *cis*)	−30.6	2.69 × 10^22^	22.43
{13} Fe^3+^-DHLA^2−^ (CO)	−28.1	4.41 × 10^20^	20.64
{14} Fe^3+^-DHLA^2−^ (COO)	−25.4	4.60 × 10^18^	18.66
{15} Fe^3+^-DHLA^2−^ (S1)	−21.9	1.13 × 10^16^	16.05
S2-deprotonated DHLA^2−^			
{16} Fe^3+^-DHLA^2−^ (CO, S2 *cis*)	−37.3	2.38 × 10^27^	27.38
{17} Fe^3+^-DHLA^2−^ (COO, S2)	−31.1	6.13 × 10^22^	22.79
{18} Fe^3+^-DHLA^2−^ (S2)	−24.5	1.01 × 10^18^	18.00
{19} Fe^3+^-DHLA^2−^ (CO)	−24.5	9.56 × 10^17^	17.98
{20} Fe^3+^-DHLA^2−^ (COO)	−23.5	1.66 × 10^17^	17.22

^a^ Coordinating atoms in the organic ligand are shown in parentheses for each complex.

**Table 4:** Standard Gibbs free energy change ($∆G_{f}^{{}^{\circ}}$, in kcal/mol) and formation constant ($K_{f}$,$\;\log K_{f}$ ) for the non-octahedral chelation of Fe(III) with LA^−^ (as per Equation (4)), and with S1- and S2-deprotonated DHLA^2−^ (as per Equation (7)) in aqueous solution at 298.15 K. ^a^

**Complex** ${\left[Fe\left(\mathrm{LA}\right){\left(H_{2}O\right)}_{n}\right]}^{2+}$	$\;∆G_{f}^{{}^{\circ}}{}_{Fe^{3+}-LA^{-}}$	$\;K_{f}{}_{Fe^{3+}-LA^{-}}\;$	$\;\log K_{f}{}_{Fe^{3+}-LA^{-}}\;$
{49} Fe^3+^ –LA^−^ (CO) ^b^	−8.3	1.30 × 10^6^	6.12
{50} Fe^3+^-LA^−^ (CO) ^c^	−16.9	2.29 × 10^12^	12.36
{51} Fe^3+^-LA^−^ (COO) ^b^	−11.0	1.15 × 10^8^	8.06
{52} Fe^3+^-LA^−^ (COO) ^c^	−14.2	2.63 × 10^10^	10.42
**Complex** ${\left[Fe\left(DHLA\right){\left(H_{2}O\right)}_{n}\right]}^{+}$	$\;∆G_{f}^{{}^{\circ}}{{}^{\prime }}_{{\mathrm{Fe}}^{3+}-{\mathrm{DHLA}}^{2-}}$	$\;K_{f}{{}^{\prime }}_{{\mathrm{Fe}}^{3+}-{\mathrm{DHLA}}^{2-}}\;$	$\;logK_{f}{{}^{\prime }}_{{\mathrm{Fe}}^{3+}-{\mathrm{DHLA}}^{2-}}\;$
{53} Fe^3+^-DHLA^2−^ (CO, S1) ^c^	−28.0	3.51 × 10^20^	20.55
{54} Fe^3+^-DHLA^2−^ (CO, S2) ^b^	−30.1	1.17 × 10^22^	22.07
{55} Fe^3+^-DHLA^2−^ (CO, S2) ^c^	−36.3	4.22 × 10^26^	26.63
{56} Fe^3+^-DHLA^2−^ (COO, S2) ^b^	−20.5	1.07 × 10^15^	15.03
{57} Fe^3+^-DHLA^2−^ (COO, S2) ^c^	−27.3	1.12 × 10^20^	20.05

^a^ Coordinating atoms in the organic ligand are shown in parentheses for each complex. ^b^ Tetra-coordinated complexes; ^c^ Penta-coordinated complexes.

**Table 5:** Rate constants (in M^−1^ s^−1^) for the reduction of Fe(III) complexes (with and without DHLA^2−^) with $O_{2}^{•-}\;$ and with ascorbate (${\mathrm{ASC}}^{-}$ ) in aqueous solution at 298.15 K and the rate constant ratios (using the reduction of ${\left[\mathrm{Fe}{\left(H_{2}O\right)}_{6}\right]}^{3+}\;$ as reference). ^a^

	$\;Ox^{-}=O_{2}^{•-}\;$		$\;Ox^{-}={\mathrm{ASC}}^{-}\;$	
Reaction	*k*_app_	Ratio	*k*_app_	Ratio
$\;{\left[\mathrm{Fe}{\left(H_{2}O\right)}_{6}\right]}^{3+}+\;Ox^{-}\to {\left[\mathrm{Fe}{\left(H_{2}O\right)}_{6}\right]}^{2+}+Ox\;$	7.71 × 10^9^		7.36 × 10^9^	
$\;\left[12\right]+\;Ox^{-}\to \left[35\right]+Ox\;$	7.08 × 10^9^	1.1	70.8	1.0 × 10^8^
$\;\left[16\right]+\;Ox^{-}\to \left[39\right]+Ox\;$	7.73 × 10^9^	0.997	2.03 × 10^3^	3.6 × 10^6^
$\;\left[14\right]+\;Ox^{-}\to \left[37\right]+Ox\;$	8.31 × 10^9^	0.928	3.18 × 10^3^	2.3 × 10^6^
$\;\left[55\right]+\;Ox^{-}\to \left[64\right]+Ox\;$	7.92 × 10^9^	0.973	9.12 × 10^3^	8.1 × 10^5^
$\;\left[13\right]+\;Ox^{-}\to \left[36\right]+Ox\;$	8.26 × 10^9^	0.933	1.20 × 10^5^	6.2 × 10^4^
$\;\left[18\right]+\;Ox^{-}\to \left[41\right]+Ox\;$	8.29 × 10^9^	0.930	4.28 × 10^5^	1.7 × 10^4^

^a^ For additional kinetic and thermodynamic information on these and other reactions studied, refer to Tables S4 and S5; k is reported instead of *k*_app_ when diffusion corrections were not necessary.

**Table 6:** Standard Gibbs free energy change ($∆G_{f}^{{}^{\circ}}{}^{\prime }$, in kcal/mol) and equilibrium constant ($K_{f}{}^{\prime }$, $\log K_{f}{}^{\prime }$ ) for the most stable Cu(II) complexes with DHLA^2−^ re-calculated at the M06(SMD)/6-31++G(d,p) level of theory in aqueous solution under physiological pH conditions (as per Equation (14)) at 298.15 K. ^a^

Complex ${\left[Cu\left(DHLA\right){\left(H_{2}O\right)}_{n}\right]}^{+}$	$\;∆G_{f}^{{}^{\circ}}{{}^{\prime }}_{Cu^{2+}-DHLA^{2-}}\;(\mathrm{kcal}/\mathrm{mol})$	$\;K_{f}{{}^{\prime }}_{Cu^{2+}-DHLA^{2-}}\;$	$\;\log K_{f}{{}^{\prime }}_{Cu^{2+}-DHLA^{2-}}\;$
{1B} Cu^2+^-DHLA^2^^−^ (CO, S2 *cis*)	−31.4	1.09 × 10^23^	23.04
{2B} Cu^2+^-DHLA^2^^−^ (CO, S2 *trans*)	−30.0	9.46 × 10^21^	21.98
{3B} Cu^2+^-DHLA^2^^−^ (CO)	−18.6	4.22 × 10^13^	13.62

^a^ Coordinating atoms in the S2-deprotonated DHLA^2−^ ligand are shown in parentheses for each complex; M06-2X(SMD)/6-31++G(d,p) values are reported in Table 6 of Reference 10; the M06 calculations were performed using as starting point the M06-2X structures reported in Reference 10.

**Table 7:** Rate constants (in M^−1^ s^−1^) for the reduction of Cu(II) complexes (with and without S2-deprotonated DHLA^2−^) with $O_{2}^{•-}$ and with ascorbate (${\mathrm{ASC}}^{-}$ ) in aqueous solution at 298.15 K and the rate constant ratios (using the reduction of ${\left[\mathrm{Cu}{\left(H_{2}O\right)}_{4}\right]}^{3+}\;$ as reference). ^a^

	$\;Ox^{-}=O_{2}^{•-}\;$		$\;Ox^{-}={\;\mathrm{ASC}}^{-}\;$	
Reaction	*k*_app_	Ratio	*k*_app_	Ratio
$\;{\left[\mathrm{Cu}{\left(H_{2}O\right)}_{4}\right]}^{2+}+\;Ox^{-}\to {\left[\mathrm{Cu}{\left(H_{2}O\right)}_{2}\right]}^{+}\cdot 2H_{2}O+Ox\;$	7.43 × 10^9^		2.51 × 10^7^	
$\;\left\{1B\right\}+\;Ox^{-}\to \left\{4B\right\}+Ox\;$	2.19 × 10^9^	3.4	3.32 × 10^3^	7560.2
$\;\left\{2B\right\}+\;Ox^{-}\to \left\{5B\right\}+Ox\;$	6.02 × 10^9^	1.2	1.00 × 10^5^	251.0
$\;\left\{3B\right\}+\;Ox^{-}\to \left\{6B\right\}+Ox\;$	4.02 × 10^9^	1.8	4.38 × 10^3^	5730.6
$\;\left\{9B\right\}+\;Ox^{-}\to \left\{10B\right\}+Ox$ ^b^	4.38 × 10^9^	1.7	3.26 × 10^3^	7699.4

^a^ For additional kinetic and thermodynamic information on these reactions, refer to Table S6; *k* is reported instead of *k*_app_ when diffusion corrections were not necessary; ^b^ Complexes {9B} and {10B} involve LA^−^.

## Supplementary Materials

The following are available online at https://www.mdpi.com/2076-3921/9/8/674/s1, Figure S1: Plots of the apparent rate constant (*k*_app_ or log *k*_app_) versus the standard Gibbs free energy of reaction (Δ*G*°) for the SET reactions of Fe(III) complexes with (a) $O_{2}^{•-}$ and (b) ${\mathrm{ASC}}^{-}$; Figure S2: Plots of the apparent rate constant (*k*_app_) for the SET reactions of Fe(III) complexes with $O_{2}^{•-}$ (left axis: *k*_app_; marker: circles) and ${\mathrm{ASC}}^{-}$ (right axis: log *k*_app_; marker: triangles) versus the $∆G_{f}^{{}^{\circ}}$ of the exergonic Fe(III) complexes studied. Table S1: Absolute enthalpies and Gibbs free energies of the different species considered in this study at the M06(SMD)/6-31++G(d,p) level of theory in water at 298.15 K, Table S2: Results of some test calculations with high- and low-spin octahedral Fe(III) and Fe(II) complexes, and non-octahedral Fe(III) aquo-complexes, Table S3: Standard Gibbs free energy change ($∆G_{f}^{{}^{\circ}}$) and formation constant ($K_{f}$, $\log\;K_{f}$) for the chelation of Fe(III) with S1- and S2-deprotonated DHLA^2−^ (as per Equation (6)) in aqueous solution at 298.15 K, Table S4: Standard Gibbs free energy of reaction (Δ*G*°, kcal/mol) and activation (Δ*G*^≠^, kcal/mol), and various rate constants (*k*, *k*_D_ and *k*_app_, M^–1^ s^–1^) for the initial reaction of the Haber-Weiss cycle (with and without iron complexation with LA^−^, DHLA^−^ or DHLA^2−^) with $O_{2}^{•-}$ in aqueous solution at 298.15 K, Table S5: Standard Gibbs free energy of reaction (Δ*G*°, kcal/mol) and activation (Δ*G*^≠^, kcal/mol), various rate constants (*k*, *k*_D_ and *k*_app_, M^–1^ s^–1^) and the rate constant ratio (using *k*_app_ for the reduction of [Fe(H_2_O)_6_]^3+^ as reference) for the initial reaction of the Haber-Weiss cycle (with and without iron complexation with LA^−^, DHLA^−^ or DHLA^2−^) with ascorbate (${\mathrm{ASC}}^{-}$) in aqueous solution at 298.15 K, Table S6: Standard Gibbs free energy of reaction (Δ*G*°, kcal/mol) and activation (Δ*G*^≠^, kcal/mol), and various rate constants (*k*, *k*_D_ and *k*_app_, M^–1^ s^–1^) for the initial reaction of the Haber-Weiss cycle (with and without copper complexation with S2-deprotonated DHLA^2−^) with $O_{2}^{•-}$ or with ascorbate (${\mathrm{ASC}}^{-}$) in aqueous solution at 298.15 K. M06(SMD)/6-31++G(d,p) Cartesian coordinates of the optimized geometries in water of the species calculated in this study.
