# Supplementary material for: Theoretical Study of the Iron Complexes with Lipoic and Dihydrolipoic Acids: Exploring Secondary Antioxidant Activity
Source: Antioxidants (Basel). 2020 Jul 28;9(8):674. doi: 10.3390/antiox9080674 (PMC7465238; doi:10.3390/antiox9080674)
Supplement: Supplementary file 1 [file antioxidants-09-00674-s001.pdf]

Theoretical Study of the Iron Complexes with Lipoic and Dihydrolipoic Acids: Exploring Secondary Antioxidant Activity

Roger Monreal-Corona, Jesse Biddlecombe, Angela Ippolito, Nelaine Mora-Diez

Thompson Rivers University, Department of Chemistry, Kamloops, B.C., V2C 0C8 Canada

Electronic Supplementary Information

(55 pages)

Contents:

Table S1. Absolute enthalpies and Gibbs free energies of the different species considered in this study at the M06(SMD)/6-31++G(d,p) level of theory in water at 298.15 K.

Table S2. Results of some test calculations with high- and low-spin octahedral Fe(III) and Fe(II) complexes, and non-octahedral Fe(III) aquo-complexes.

Table S3. Standard Gibbs free energy change (ΔG_f°) and formation constant (K_f, $log\ K_f$) for the chelation of Fe(III) with S1- and S2-deprotonated $DHLA^{2-}$ (as per eq (6)) in aqueous solution at 298.15 K.

Table S4. Standard Gibbs free energy of reaction (ΔG°, kcal/mol) and activation (ΔG≠, kcal/mol), and various rate constants (k, k_D and k_{app}, $M^{-1}\ s^{-1}$) for the initial reaction of the Haber-Weiss cycle (with and without iron complexation with LA^-, $DHLA^-$ or $DHLA^{2-}$) with $O_2^{\bullet-}$ in aqueous solution at 298.15 K.

Table S5. Standard Gibbs free energy of reaction (ΔG°, kcal/mol) and activation (ΔG≠, kcal/mol), various rate constants (k, k_D and k_{app}, $M^{-1}\ s^{-1}$) and the rate constant ratio (using k_{app} for the reduction of $[Fe(H_2O)_6]^{3+}$as reference) for the initial reaction of the Haber-Weiss cycle (with and without iron complexation with LA^-, $DHLA^-$ or $DHLA^{2-}$) with ascorbate (ASC^-) in aqueous solution at 298.15 K.

Table S6. Standard Gibbs free energy of reaction (ΔG°, kcal/mol) and activation (ΔG≠, kcal/mol), and various rate constants (k, k_D and k_{app}, $M^{-1}\ s^{-1}$) for the initial reaction of the Haber-Weiss cycle (with and without copper complexation with S2-deprotonated $DHLA^{2-}$) with $O_2^{\bullet-}$or with ascorbate (ASC^-) in aqueous solution at 298.15 K.

Figure S1. Plots of the apparent rate constant (k_{app} or log k_{app}) versus the standard Gibbs free energy of reaction (ΔG°) for the SET reactions of Fe(III) complexes with (a) $O_2^{\bullet-}$ and (b) ASC^-.

Figure S2. Plots of the apparent rate constant (k_{app}) for the SET reactions of Fe(III) complexes with $O_2^{\bullet-}$ (left axis: k_{app}; marker: circles) and ASC^- (right axis: log k_{app}; marker: triangles) *versus* the ΔG_f° of the exergonic Fe(III) complexes studied.

M06(SMD)/6-31++G(d,p) Cartesian coordinates of the optimized geometries in water of the species calculated in this study.

Table S1. Absolute enthalpies and Gibbs free energies of the different species considered in this study at the M06(SMD)/6-31++G(d,p) level of theory in water at 298.15 K.[c]

	Species	H° (au)	G° (au)
{1}	Fe^{3+} – LA^- (CO)	-2904.310630	-2904.393351
{2}	Fe^{3+} – LA^- (COO)	-2827.910831	-2827.990222
{3}	Fe^{3+} – LA^- (S1)	-2904.265906	-2904.349319
{4}	Fe^{3+} – LA^- (S2)	-2904.262193	-2904.345795
{5}	Fe^{3+} – $DHLA^-$ (CO)	-2905.486338	-2905.569775
{6}	Fe^{3+} – $DHLA^-$ (COO)	-2829.090390	-2829.168072
{7}	Fe^{3+} – $DHLA^-$ (CO)[a]	-2829.087779	-2829.167353
{8}	Fe^{3+} – $DHLA^-$ (CO)[a]	-2829.089177	-2829.166194
{9}	Fe^{3+} – $DHLA^-$ (S2)	-2905.434600	-2905.518985
{10}	Fe^{3+} – $DHLA^-$ (S1)	-2905.432621	-2905.518033
{11}	Fe^{3+} – $DHLA^-$ (S1, S2 *cis*)	-2829.030063	-2829.109787
{12}	Fe^{3+} – $DHLA^{2-}$ (CO, S1 *cis*)	-2828.649572	-2828.730514
{13}	Fe^{3+} – $DHLA^{2-}$ (CO)	-2905.033839	-2905.127016
{14}	Fe^{3+} – $DHLA^{2-}$ (COO)	-2828.636813	-2828.722329
{15}	Fe^{3+} – $DHLA^{2-}$ (S1)	-2905.030842	-2905.117039
{16}	Fe^{3+} – $DHLA^{2-}$ (CO, S2 *cis*)	-2828.663502	-2828.742664
{17}	Fe^{3+} – $DHLA^{2-}$ (COO, S2)	-2752.257484	-2752.333444
{18}	Fe^{3+} – $DHLA^{2-}$ (S2)	-2905.034964	-2905.122684
{19}	Fe^{3+} – $DHLA^{2-}$ (CO)	-2905.030357	-2905.122632
{20}	Fe^{3+} – $DHLA^{2-}$ (COO)	-2828.639861	-2828.720597
{21}	$[Fe(H_2O)_6]^{3+}$	-1721.514801	-1721.569896
{22}	H_2O	-76.388360	-76.408751
{23}	$(H_2O)_2$	-152.776673	-152.809134
{24}	$(H_2O)_3$	-229.165817	-229.208383
{25}	LA^-	-1259.146591	-1259.203383
{26}	$DHLA^-$	-1260.324327	-1260.384635
{27}	S1-deprotonated $DHLA^{2-}$	-1259.854788	-1259.913848
{28}	S2-deprotonated $DHLA^{2-}$	-1259.853829	-1259.915251
{29}	Fe^{2+} – LA^- (CO)	-2904.485164	-2904.577230
{30}	Fe^{2+} – LA^- (COO)	-2828.088865	-2828.174349
{31}	Fe^{2+} – $DHLA^-$ (CO)	-2905.666154	-2905.754376
{32}	Fe^{2+} – $DHLA^-$ (COO)	-2829.270335	-2829.358285
{33}	Fe^{2+} – $DHLA^-$ (CO)[a]	-2829.267179	-2829.351877
{34}	Fe^{2+} – $DHLA^-$ (CO)[a]	-2829.267823	-2829.352458
{35}	Fe^{2+} – $DHLA^{2-}$ (CO, S1 *cis*)	-2828.805518	-2828.887945
{36}	Fe^{2+} – $DHLA^{2-}$ (CO)	-2905.202688	-2905.291499
{37}	Fe^{2+} – $DHLA^{2-}$ (COO)	-2828.798942	-2828.884314

{38}	Fe^{2+} – $DHLA^{2-}$ (S1)	-2905.197641	-2905.292134
{39}	Fe^{2+} – $DHLA^{2-}$ (CO, S2 *cis*)	-2828.822357	-2828.904069
{40}	Fe^{2+} – $DHLA^{2-}$ (CO, S2)[a]	-2752.422940	-2752.504160
{41}	Fe^{2+} – $DHLA^{2-}$ (S2)	-2905.197979	-2905.289285
{42}	Fe^{2+} – $DHLA^{2-}$ (CO)	-2905.208548	-2905.298339
{43}	Fe^{2+} – $DHLA^{2-}$ (COO)	-2828.822408	-2828.907116
{44}	$[Fe(H_2O)_6]^{3+}$ (low spin)	-1721.422174	-1721.470651
{45}	Fe^{3+} – $DHLA^{2-}$ (CO, S2 *cis*) (low spin)	-2828.596852	-2828.671207
{46}	$[Fe(H_2O)_6]^{2+}$ (low spin)	-1721.625328	-1721.677381
{47}	Fe^{2+} – $DHLA^{2-}$ (CO, S2 *cis*) (low spin)	-2828.751192	-2828.827236
{48}	ASC^{-}	-683.903498	-683.954625
{49}	Fe^{3+} – LA^{-} (CO)[b]	-2751.503936	-2751.578182
{50}	Fe^{3+} – LA^{-} (CO)[a]	-2827.910541	-2827.990999
{51}	Fe^{3+} – LA^{-} (COO)[b]	-2675.110116	-2675.182608
{52}	Fe^{3+} – LA^{-} (COO)[a]	-2751.510889	-2751.587533
{53}	Fe^{3+} – $DHLA^{2-}$ (CO, S1)[a]	-2752.250947	-2752.327169
{54}	Fe^{3+} – $DHLA^{2-}$ (CO, S2)[b]	-2675.862431	-2675.932076
{55}	Fe^{3+} – $DHLA^{2-}$ (CO, S2)[a]	-2752.266939	-2752.341782
{56}	Fe^{3+} – $DHLA^{2-}$ (COO, S2)[b]	-2599.458334	-2599.522629
{57}	Fe^{3+} – $DHLA^{2-}$ (COO, S2)[a]	-2675.856452	-2675.927694
{58}	Fe^{2+} – LA^{-} (CO)[b]	-2751.690579	-2751.769700
{59}	Fe^{2+} – LA^{-} (CO)[a]	-2828.087369	-2828.173685
{60}	Fe^{2+} – LA^{-} (COO)[b]	-2675.293087	-2675.366707
{61}	Fe^{2+} – LA^{-} (COO)[a]	-2751.689578	-2751.771226
{62}	Fe^{2+} – $DHLA^{2-}$ (CO, S1)[a]	-2752.423115	-2752.502122
{63}	Fe^{2+} – $DHLA^{2-}$ (CO, S2)[b]	-2676.030897	-2676.103401
{64}	Fe^{2+} – $DHLA^{2-}$ (CO, S2)[a]	-2752.427628	-2752.504563
{65}	Fe^{2+} – $DHLA^{2-}$ (COO, S2)[b]	-2599.627129	-2599.693951
{66}	Fe^{2+} – $DHLA^{2-}$ (COO, S2)[b]	-2676.029814	-2676.105222
{67}	$[Fe(H_2O)_5]^{3+}$	-1645.114009	-1645.165232
{68}	$[Fe(H_2O)_5]^{3+}$ (low spin)	-1645.008949	-1645.054679
{69}	$[Fe(H_2O)_4]^{3+}$	-1568.713305	-1568.760647
{70}	$[Fe(H_2O)_4]^{3+}$ (low spin)	-1568.592218	-1568.633909
{1B}	Cu^{2+} – $DHLA^{2-}$ (CO, S2 *cis*)	-3052.698628	-3052.768559
{2B}	Cu^{2+} – $DHLA^{2-}$ (CO, S2 *trans*)	-3052.697006	-3052.766255
{3B}	Cu^{2+} – $DHLA^{2-}$ (CO)	-3129.072236	-3129.148495

{4B}	Cu^+ – $DHLA^{2-}$ (S2)	-3052.866508	-3052.940130
{5B}	Cu^+ – $DHLA^{2-}$ (CO, S2)	-3052.869487	-3052.942656
{6B}	Cu^+ – $DHLA^{2-}$ (CO)	-3129.239033	-3129.317092
{7B}	$[Cu(H_2O)_4]^{2+}$	-1945.557169	-1945.605222
{8B}	$[Cu(H_2O)_2]^+ \cdot 2H_2O$	-1945.732729	-1945.786644
{9B}	Cu^{2+} – LA^- (CO)	-3128.345256	-3128.421147
{10B}	Cu^+ – LA^- (CO)	-3128.507228	-3128.588128

[a] Pentacoordinated complexes; [b]Tetracoordinated complexes; [c] All iron complexes are high spin, unless otherwise indicated.

Table S2. Absolute Gibbs free energies of some test calculations with high- and low-spin octahedral Fe(III) and Fe(II) complexes, and non-octahedral Fe(III) aquo-complexes.

G° (au)	$[Fe(H_2O)_6]^{3+}$	$[Fe(H_2O)_6]^{2+}$	Fe^{3+} $DHLA^{2-}$ (CO, S2)	Fe^{2+} $DHLA^{2-}$ (CO, S2)
**high spin**	-1721.569896	-1721.763408	-2828.742664	-2828.904069
**low spin**	-1721.470651	-1721.677381	-2828.671207	-2828.827236

G° (au)	$[Fe(H_2O)_5]^{3+}$	$[Fe(H_2O)_4]^{3+}$
**high spin**	-1645.165232	-1568.760647
**low spin**	-1645.054679	-1568.633909

Table S3. Standard Gibbs free energy change (ΔG_f°) and formation constant (K_f, $logK_f$) for the chelation of Fe(III) with S1- and S2-deprotonated $DHLA^{2-}$ (as per eq (6)) in aqueous solution at 298.15 K.[a]

**COMPLEX** $[\boldsymbol{Fe}(\boldsymbol{DHLA})(\boldsymbol{H_2O})_{\boldsymbol{n}}]^+$	$\Delta \boldsymbol{G}^{\circ}_{\boldsymbol{f}_{Fe^{3+}-DHLA^{2-}}}$ **(kcal/mol)**	$\boldsymbol{K}_{\boldsymbol{f}_{Fe^{3+}-DHLA^{2-}}}$	$\boldsymbol{logK}_{\boldsymbol{f}_{Fe^{3+}-DHLA^{2-}}}$
S1-deprotonated $DHLA^{2-}$			
**{12} Fe^{3+} – $DHLA^{2-}$ (CO, S1 *cis*)**	-35.1	5.38 x 10^{25}	25.73
**{13} Fe^{3+} – $DHLA^{2-}$ (CO)**	-32.6	8.81 x 10^{23}	23.94
**{14} Fe^{3+} – $DHLA^{2-}$ (COO)**	-29.9	9.20 x 10^{21}	21.96
**{15} Fe^{3+} – $DHLA^{2-}$ (S1)**	-26.4	2.25 x 10^{19}	19.35
S2-deprotonated $DHLA^{2-}$			
**{16} Fe^{3+} – $DHLA^{2-}$ (CO, S2 *cis*)**	-41.8	4.76 x 10^{30}	30.68
**{17} Fe^{3+} – $DHLA^{2-}$ (COO, S2)**	-35.6	1.23 x 10^{26}	26.09
**{18} Fe^{3+} – $DHLA^{2-}$ (S2)**	-29.0	2.02 x 10^{21}	21.31
**{19} Fe^{3+} – $DHLA^{2-}$ (CO)**	-29.0	1.91 x 10^{21}	21.28
**{20} Fe^{3+} – $DHLA^{2-}$ (COO)**	-28.0	3.32 x 10^{20}	20.52

[a] Coordinating atoms in the organic ligand are shown in parentheses for each complex.

Table S4. Standard Gibbs free energy of reaction (ΔG°, kcal/mol) and activation (ΔG≠, kcal/mol), various rate constants (k, k_D and k_{app}, $M^{-1} s^{-1}$) and the rate constant ratio (using k_{app} for the reduction of $[Fe(H_2O)_6]^{3+}$ as reference) for the initial reaction of the Haber-Weiss cycle (with and without iron complexation with LA^-, $DHLA^-$ or $DHLA^{2-}$) with $O_2^{\bullet-}$ in aqueous solution at 298.15 K.

Reaction	ΔG⁰	ΔG≠	k	k_D	k_{app}	Ratio
$[Fe(H_2O)_6]^{3+} + O_2^{\bullet-} \rightarrow [Fe(H_2O)_6]^{2+} + O_2$	-28.0	0.8	1.67 x 10^{12}	7.75 x 10^9	7.71 x 10^9	
$\{\mathbf{12}\} + O_2^{\bullet-} \rightarrow \{\mathbf{35}\} + O_2$	-5.4	2.8	5.47 x 10^{10}	8.13 x 10^9	7.08 x 10^9	1.1
$\{\mathbf{16}\} + O_2^{\bullet-} \rightarrow \{\mathbf{39}\} + O_2$	-7.9	2.5	9.63 x 10^{10}	8.41 x 10^9	7.73 x 10^9	0.997
$\{\mathbf{55}\} + O_2^{\bullet-} \rightarrow \{\mathbf{64}\} + O_2$	-8.8	1.9	2.37 x 10^{11}	8.19 x 10^9	7.92 x 10^9	0.973
$\{\mathbf{17}\} + O_2^{\bullet-} \rightarrow \{\mathbf{40}\} + O_2$	-13.7	1.1	9.96 x 10^{11}	8.12 x 10^9	8.05 x 10^9	0.958
$\{\mathbf{53}\} + O_2^{\bullet-} \rightarrow \{\mathbf{62}\} + O_2$	-16.4	1.2	8.63 x 10^{11}	8.16 x 10^9	8.09 x 10^9	0.953
$\{\mathbf{7}\} + O_2^{\bullet-} \rightarrow \{\mathbf{33}\} + O_2$	-22.4	0.0	6.01 x 10^{12}	8.10 x 10^9	8.09 x 10^9	0.953
$\{\mathbf{56}\} + O_2^{\bullet-} \rightarrow \{\mathbf{65}\} + O_2$	-14.1	0.1	5.32 x 10^{12}	8.16 x 10^9	8.15 x 10^9	0.946
$\{\mathbf{51}\} + O_2^{\bullet-} \rightarrow \{\mathbf{60}\} + O_2$	-22.1	0.4	3.15 x 10^{12}	8.19 x 10^9	8.17 x 10^9	0.944
$\{\mathbf{54}\} + O_2^{\bullet-} \rightarrow \{\mathbf{63}\} + O_2$	-14.1	1.0	1.20 x 10^{12}	8.24 x 10^9	8.18 x 10^9	0.943
$\{\mathbf{19}\} + O_2^{\bullet-} \rightarrow \{\mathbf{42}\} + O_2$	-16.9	0.7	2.04 x 10^{12}	8.23 x 10^9	8.19 x 10^9	0.941
$\{\mathbf{8}\} + O_2^{\bullet-} \rightarrow \{\mathbf{34}\} + O_2$	-23.5	0.1	5.34 x 10^{12}	8.20 x 10^9	8.19 x 10^9	0.941
$\{\mathbf{6}\} + O_2^{\bullet-} \rightarrow \{\mathbf{32}\} + O_2$	-26.0	0.0	5.92 x 10^{12}	8.24 x 10^9	8.23 x 10^9	0.937
$\{\mathbf{49}\} + O_2^{\bullet-} \rightarrow \{\mathbf{58}\} + O_2$	-26.8	1.0	1.11 x 10^{12}	8.33 x 10^9	8.26 x 10^9	0.933
$\{\mathbf{13}\} + O_2^{\bullet-} \rightarrow \{\mathbf{36}\} + O_2$	-9.8	0.2	4.33 x 10^{12}	8.28 x 10^9	8.26 x 10^9	0.933
$\{\mathbf{2}\} + O_2^{\bullet-} \rightarrow \{\mathbf{30}\} + O_2$	-22.2	0.0	6.07 x 10^{12}	8.27 x 10^9	8.26 x 10^9	0.933
$\{\mathbf{1}\} + O_2^{\bullet-} \rightarrow \{\mathbf{29}\} + O_2$	-22.0	0.1	5.26 x 10^{12}	8.29 x 10^9	8.27 x 10^9	0.932
$\{\mathbf{18}\} + O_2^{\bullet-} \rightarrow \{\mathbf{41}\} + O_2$	-11.2	0.9	1.36 x 10^{12}	8.34 x 10^9	8.29 x 10^9	0.930
$\{\mathbf{57}\} + O_2^{\bullet-} \rightarrow \{\mathbf{66}\} + O_2$	-18.0	0.5	2.64 x 10^{12}	8.31 x 10^9	8.29 x 10^9	0.930
$\{\mathbf{52}\} + O_2^{\bullet-} \rightarrow \{\mathbf{61}\} + O_2$	-21.9	0.0	6.21 x 10^{12}	8.30 x 10^9	8.29 x 10^9	0.930
$\{\mathbf{14}\} + O_2^{\bullet-} \rightarrow \{\mathbf{37}\} + O_2$	-8.3	0.3	3.99 x 10^{12}	8.32 x 10^9	8.31 x 10^9	0.928
$\{\mathbf{15}\} + O_2^{\bullet-} \rightarrow \{\mathbf{38}\} + O_2$	-16.5	0.4	3.37 x 10^{12}	8.40 x 10^9	8.38 x 10^9	0.920

$\{\mathbf{50}\} + O_2^{\bullet -} \rightarrow \{\mathbf{59}\} + O_2$	-21.8	0.0	5.79 x 10^{12}	8.40 x 10^9	8.39 x 10^9	0.919
$\{\mathbf{20}\} + O_2^{\bullet -} \rightarrow \{\mathbf{43}\} + O_2$	-23.7	0.0	6.06 x 10^{12}	8.42 x 10^9	8.40 x 10^9	0.918
$\{\mathbf{5}\} + O_2^{\bullet -} \rightarrow \{\mathbf{31}\} + O_2$	-22.5	0.0	6.13 x 10^{12}	8.42 x 10^9	8.41 x 10^9	0.917

Table S5. Standard Gibbs free energy of reaction (ΔG°, kcal/mol) and activation (ΔG≠, kcal/mol), various rate constants (k, k_D and k_{app}, M^{-1} s^{-1}) and the rate constant ratio (using k_{app} for the reduction of $[Fe(H_2O)_6]^{3+}$as reference) for the initial reaction of the Haber-Weiss cycle (with and without iron complexation with LA^-, $DHLA^-$ or $DHLA^{2-}$) with ascorbate (ASC^-) in aqueous solution at 298.15 K.

Reaction	ΔG⁰	ΔG≠	k	k_D	k_{app}	Ratio
$[Fe(H_2O)_6]^{3+} + ASC^- \rightarrow [Fe(H_2O)_6]^{2+} + ASC^\bullet$	-7.8	1.1	1.05 x 10^{12}	7.41 x 10^9	7.36 x 10^9	
$\{\mathbf{12}\} + ASC^- \rightarrow \{\mathbf{35}\} + ASC^\bullet$	14.9	14.9	70.8	-	-	1.0 x 10^8
$\{\mathbf{16}\} + ASC^- \rightarrow \{\mathbf{39}\} + ASC^\bullet$	12.4	12.9	2.03 x 10^3	-	-	3.6 x 10^6
$\{\mathbf{14}\} + ASC^- \rightarrow \{\mathbf{37}\} + ASC^\bullet$	12.0	12.7	3.18 x 10^3	-	-	2.3 x 10^6
$\{\mathbf{55}\} + ASC^- \rightarrow \{\mathbf{64}\} + ASC^\bullet$	11.5	12.0	9.12 x 10^3	-	-	8.1 x 10^5
$\{\mathbf{13}\} + ASC^- \rightarrow \{\mathbf{36}\} + ASC^\bullet$	10.5	10.5	1.20 x 10^5	-	-	6.2 x 10^4
$\{\mathbf{18}\} + ASC^- \rightarrow \{\mathbf{41}\} + ASC^\bullet$	9.1	9.8	4.28 x 10^5	-	-	1.7 x 10^4
$\{\mathbf{17}\} + ASC^- \rightarrow \{\mathbf{40}\} + ASC^\bullet$	6.5	8.8	2.36 x 10^6	-	-	3118.6
$\{\mathbf{54}\} + ASC^- \rightarrow \{\mathbf{63}\} + ASC^\bullet$	6.2	8.5	3.86 x 10^6	-	-	1906.7
$\{\mathbf{53}\} + ASC^- \rightarrow \{\mathbf{62}\} + ASC^\bullet$	3.9	8.0	8.27 x 10^6	-	-	890.0
$\{\mathbf{19}\} + ASC^- \rightarrow \{\mathbf{42}\} + ASC^\bullet$	3.4	7.1	4.19 x 10^7	-	-	175.7
$\{\mathbf{56}\} + ASC^- \rightarrow \{\mathbf{65}\} + ASC^\bullet$	6.2	7.0	4.94 x 10^7	-	-	149.0
$\{\mathbf{15}\} + ASC^- \rightarrow \{\mathbf{38}\} + ASC^\bullet$	3.8	6.6	9.05 x 10^7	-	-	81.7
$\{\mathbf{57}\} + ASC^- \rightarrow \{\mathbf{66}\} + ASC^\bullet$	2.3	6.4	1.16 x 10^8	7.51 x 10^9	1.14 x 10^8	64.6
$\{\mathbf{1}\} + ASC^- \rightarrow \{\mathbf{29}\} + ASC^\bullet$	-1.7	4.4	3.54 x 10^9	7.50 x 10^9	2.41 x 10^9	3.1
$\{\mathbf{50}\} + ASC^- \rightarrow \{\mathbf{59}\} + ASC^\bullet$	-1.5	4.2	5.39 x 10^9	7.54 x 10^9	3.14 x 10^9	2.3
$\{\mathbf{8}\} + ASC^- \rightarrow \{\mathbf{34}\} + ASC^\bullet$	-3.2	4.1	6.00 x 10^9	7.48 x 10^9	3.33 x 10^9	2.2

$\{\mathbf{7}\} + ASC^{-} \rightarrow \{\mathbf{33}\} + ASC^{\bullet}$	-2.1	3.9	8.65 x 10^{9}	7.45 x 10^{9}	4.00 x 10^{9}	1.8
$\{\mathbf{2}\} + ASC^{-} \rightarrow \{\mathbf{30}\} + ASC^{\bullet}$	-1.9	3.9	8.69 x 10^{9}	7.50 x 10^{9}	4.02 x 10^{9}	1.8
$\{\mathbf{5}\} + ASC^{-} \rightarrow \{\mathbf{31}\} + ASC^{\bullet}$	-2.2	3.8	1.07 x 10^{10}	7.55 x 10^{9}	4.43 x 10^{9}	1.7
$\{\mathbf{20}\} + ASC^{-} \rightarrow \{\mathbf{43}\} + ASC^{\bullet}$	-3.4	3.6	1.36 x 10^{10}	7.54 x 10^{9}	4.85 x 10^{9}	1.5
$\{\mathbf{52}\} + ASC^{-} \rightarrow \{\mathbf{61}\} + ASC^{\bullet}$	-1.6	3.6	1.38 x 10^{10}	7.51 x 10^{9}	4.86 x 10^{9}	1.5
$\{\mathbf{6}\} + ASC^{-} \rightarrow \{\mathbf{32}\} + ASC^{\bullet}$	-5.7	2.6	7.83 x 10^{10}	7.49 x 10^{9}	6.84 x 10^{9}	1.1
$\{\mathbf{51}\} + ASC^{-} \rightarrow \{\mathbf{60}\} + ASC^{\bullet}$	-1.9	2.3	1.22 x 10^{11}	7.47 x 10^{9}	7.04 x 10^{9}	1.0
$\{\mathbf{49}\} + ASC^{-} \rightarrow \{\mathbf{58}\} + ASC^{\bullet}$	-6.5	1.0	1.14 x 10^{12}	7.52 x 10^{9}	7.47 x 10^{9}	0.985

Table S6. Standard Gibbs free energy of reaction (ΔG°, kcal/mol) and activation (ΔG≠, kcal/mol), various rate constants (k, k_D and k_{app}, M^{-1} s^{-1}) and the rate constant ratio (using k or k_{app} and the reduction of $[Cu(H_2O)_4]^{2+}$ as reference) for the initial reaction of the Haber-Weiss cycle (with and without copper complexation with S2-deprotonated $DHLA^{2-}$) with $O_2^{\bullet-}$ or with ascorbate (ASC^{-}) in aqueous solution at 298.15 K.

Reaction	ΔG^0	ΔG≠	k	k_D	k_{app}	Ratio
$[Cu(H_2O)_4]^{2+} + O_2^{\bullet-} \rightarrow [Cu(H_2O)_2]^{+} \cdot 2H_2O + O_2$	-20.5	1.3	6.50 x 10^{11}	7.52 x 10^{9}	7.43 x 10^{9}	
$\{\mathbf{1B}\} + O_2^{\bullet-} \rightarrow \{\mathbf{4B}\} + O_2$	-14.3	4.5	3.00 x 10^{9}	8.14 x 10^{9}	2.19 x 10^{9}	3.4
$\{\mathbf{2B}\} + O_2^{\bullet-} \rightarrow \{\mathbf{5B}\} + O_2$	-17.3	3.3	2.31 x 10^{10}	8.15 x 10^{9}	6.02 x 10^{9}	1.2
$\{\mathbf{3B}\} + O_2^{\bullet-} \rightarrow \{\mathbf{6B}\} + O_2$	-12.4	4.0	7.82 x 10^{9}	8.28 x 10^{9}	4.02 x 10^{9}	1.8
$\{\mathbf{9B}\} + O_2^{\bullet-} \rightarrow \{\mathbf{10B}\} + O_2{}^{a}$	-11.4	3.8	9.44 x 10^{9}	8.18 x 10^{9}	4.38 x 10^{9}	1.7
$[Cu(H_2O)_4]^{2+} + ASC^{-} \rightarrow [Cu(H_2O)_2]^{+} \cdot 2H_2O + ASC^{\bullet}$	-0.2	7.4	2.51 x 10^{7}	-	-	
$\{\mathbf{1B}\} + ASC^{-} \rightarrow \{\mathbf{4B}\} + ASC^{\bullet}$	6.0	12.6	3.32 x 10^{3}	-	-	7560.2
$\{\mathbf{2B}\} + ASC^{-} \rightarrow \{\mathbf{5B}\} + ASC^{\bullet}$	3.0	10.6	1.00 x 10^{5}	-	-	251.0
$\{\mathbf{3B}\} + ASC^{-} \rightarrow \{\mathbf{6B}\} + ASC^{\bullet}$	7.9	12.5	4.38 x 10^{3}	-	-	5730.6
$\{\mathbf{9B}\} + ASC^{-} \rightarrow \{\mathbf{10B}\} + ASC^{\bullet a}$	8.9	12.7	3.26 x 10^{3}	-	-	7699.4

[a] Complexes {9B} and {10B} involve LA^{-}

(a)

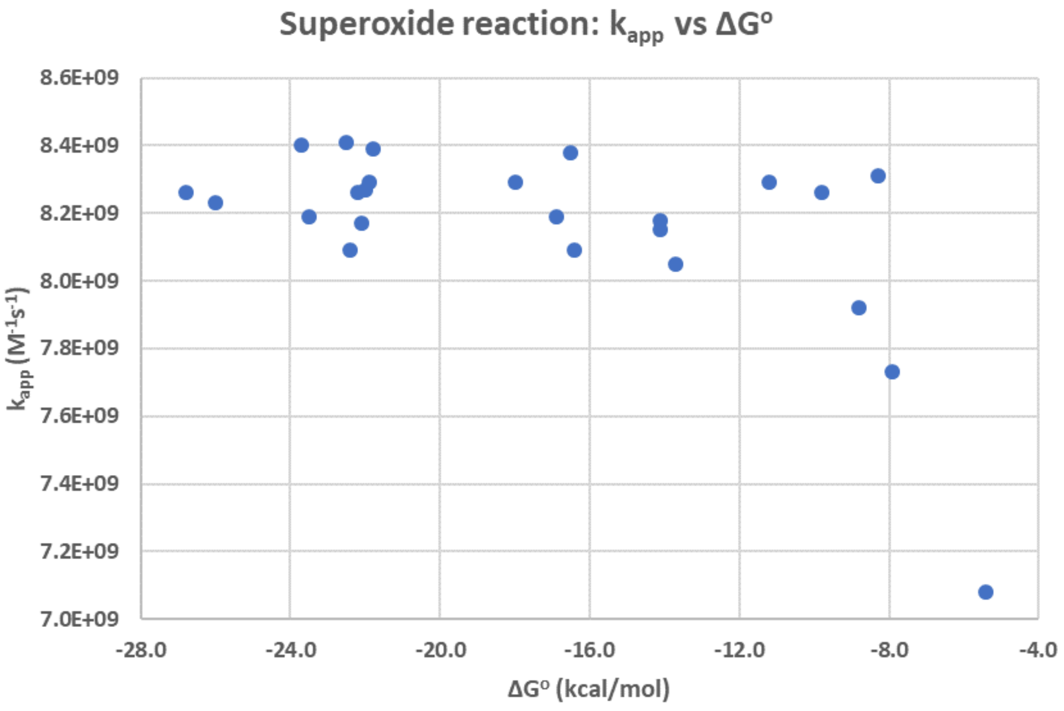

(b)

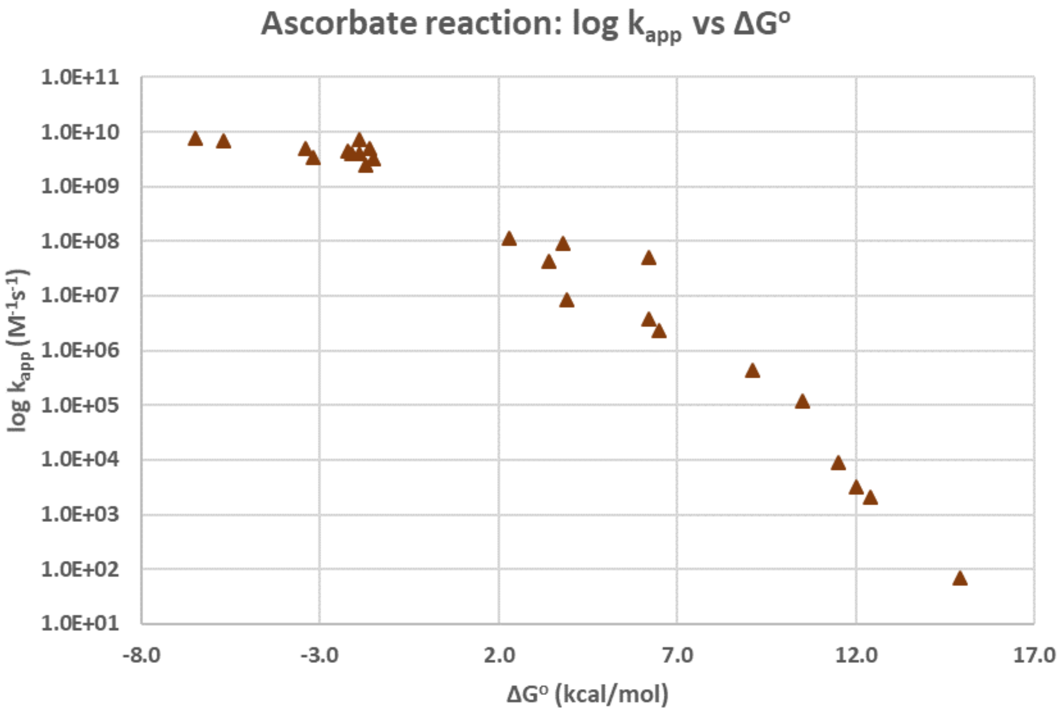

Figure S1. Plots of the apparent rate constant (k_{app} or log k_{app}) *versus* the standard Gibbs free energy of reaction (ΔG°) for the SET reactions of Fe(III) complexes with (a) $O_2^{\bullet-}$ and (b) ASC^-.

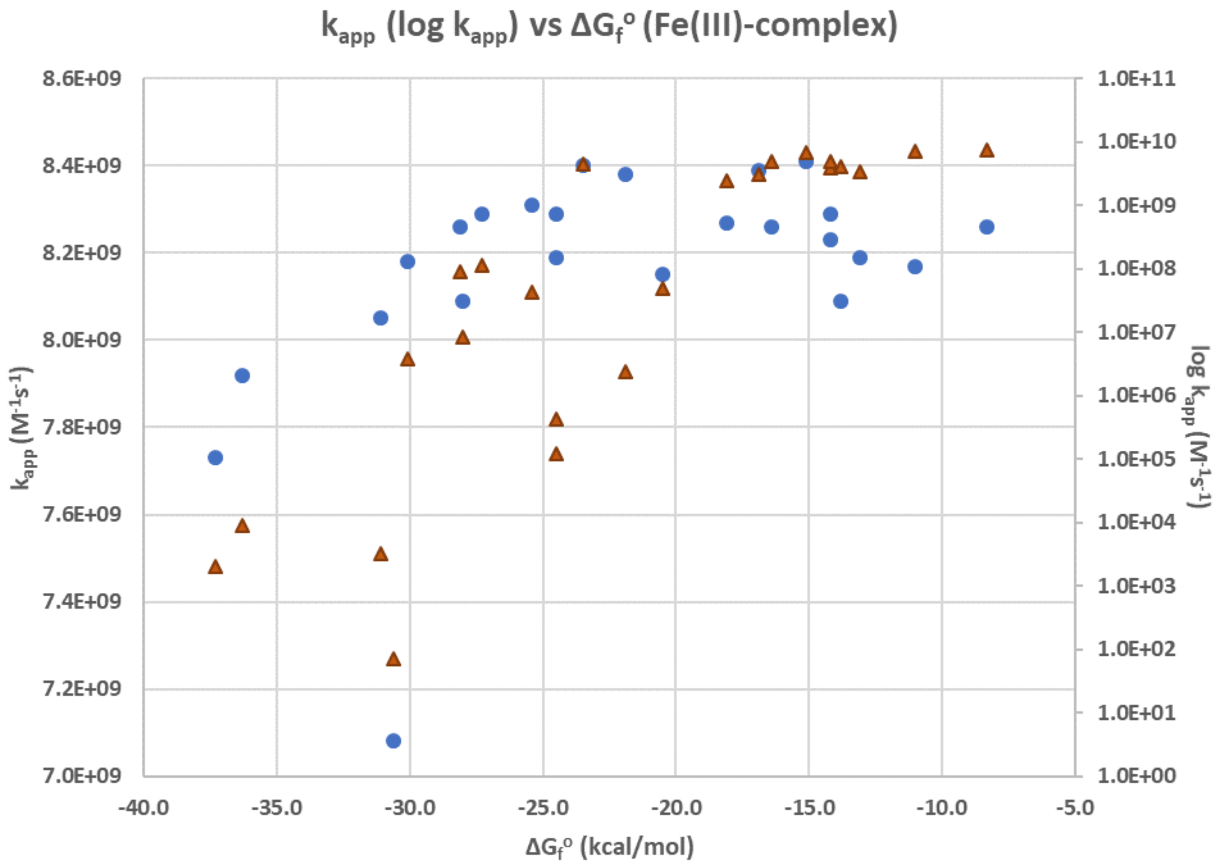

Figure S2. Plots of the apparent rate constant (k_{app}) for the SET reactions of Fe(III) complexes with $O_2^{\bullet-}$ (left axis: k_{app}; marker: circles) and ASC^- (right axis: log k_{app}; marker: triangles) *versus* the ΔG_f° of the exergonic Fe(III) complexes studied.

M06(SMD)/6-31++G(d,p) Cartesian coordinates of the optimized geometries in water of the species calculated in this study.

{1} Fe^{3+} – LA^{-} (CO)

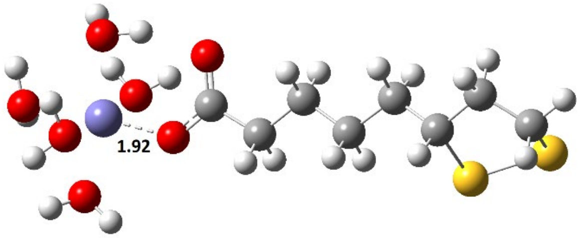


```
Charge = 2 Multiplicity = 6
 C,0,-7.1107144706,0.4983576525,-0.2930481981
 H,0,-7.2950770067,0.1390166544,-1.3126060154
 H,0,-7.9584075812,1.1124733955,0.0282126901
 C,0,-5.7882307308,1.2245692595,-0.1799514864
 H,0,-5.6850037409,1.6424420249,0.8314546762
 H,0,-5.747579742,2.0598787733,-0.891912739
 C,0,-4.6207598605,0.287100716,-0.4600012217
 H,0,-4.5067649466,0.1496857945,-1.5440934586
 S,0,-7.0105296325,-0.9232901787,0.8503864421
 S,0,-5.0644969087,-1.4227662574,0.1743195494
 C,0,-3.312928188,0.7738657733,0.1335391997
 H,0,-3.1511957931,1.802381277,-0.2299904448
 H,0,-3.4148814113,0.8387145979,1.2285464509
 C,0,-2.112452078,-0.0805753446,-0.2349015343
 H,0,-2.2288317851,-1.0902658093,0.1927520629
 H,0,-2.0811207327,-0.2107905523,-1.3288633426
 C,0,-0.7964559107,0.5160331399,0.2378635718
 H,0,-0.8303950558,0.6790411332,1.3251400556
 H,0,-0.6579492772,1.5060138665,-0.2206819965
 C,0,0.3742694555,-0.3802577681,-0.1135799234
 H,0,0.2839965646,-1.3599194894,0.3780259531
 H,0,0.3853032722,-0.6008435843,-1.1913394031
 C,0,1.7303442379,0.1639048924,0.2265799591
 O,0,2.715651728,-0.6138162112,-0.1371873673
 O,0,1.8946517424,1.2556298285,0.7881317729
 Fe,0,4.6009944938,-0.2823926726,0.0077898917
 O,0,6.8089034311,-0.2002014032,0.2170449719
 O,0,4.560040922,1.782856701,0.5527684642
 H,0,5.0121232249,1.8538854953,1.4086403077
 H,0,3.5905844029,1.847063612,0.7513770532
 H,0,7.0596975177,-0.8504237758,0.8920116861
 H,0,7.0400577334,0.661032736,0.5985696775
 O,0,4.5647014298,-0.787582931,2.1167508239
 O,0,4.8196112919,0.3135036618,-2.0291318064
 H,0,5.288086994,-0.3064439133,2.5489637217
 H,0,3.7464769057,-0.413650192,2.4821511808
 H,0,5.0607082455,1.2530134532,-2.0636548391
 H,0,5.5728509188,-0.1634232097,-2.4125965495
 O,0,5.1738990885,-2.2632279461,-0.5193588577
 H,0,4.4067428803,-2.742234588,-0.8714610674
 H,0,5.8251798615,-2.2436418417,-1.2395676514
```

{2} Fe^{3+} – LA^{-} (COO)

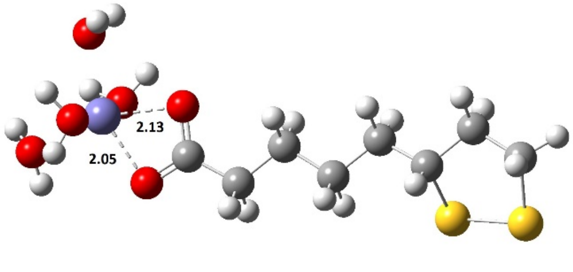


```
Charge = 2 Multiplicity = 6
C,0,4.2354454851,1.1810187496,0.1388950983
H,0,4.3716922471,1.4667721062,1.1863866442
H,0,5.0357542711,1.6267535142,-0.4584681822
C,0,2.8617081211,1.5769570298,-0.3646312288
H,0,2.8468674099,1.6103758609,-1.462636357
H,0,2.6162524734,2.5823644682,0.0046014278
C,0,1.8098079691,0.5912453771,0.1120642832
H,0,1.7838432511,0.5643270101,1.2121365679
S,0,4.4129656894,-0.6642988855,0.0591821581
S,0,2.3951658651,-1.0624352312,-0.467248212
C,0,0.425538257,0.8762991476,-0.4378085264
H,0,0.1478792138,1.8858264142,-0.0960311387
H,0,0.4726794051,0.9209031488,-1.5376640468
C,0,-0.644449188,-0.1114781217,-0.0007418076
H,0,-0.4509432311,-1.1018229927,-0.4440178875
```

H,0,-0.59587436,-0.2469564443,1.0916157572
C,0,-2.037530101,0.3501529876,-0.3945389943
H,0,-2.0795610222,0.51426581,-1.4811808102
H,0,-2.2443588387,1.3220115348,0.0757722035
C,0,-3.1023054335,-0.6514775207,0.0134158073
H,0,-2.9487723932,-1.6257568398,-0.4722026345
H,0,-3.060133185,-0.8566890399,1.0938082425
C,0,-4.4970680886,-0.2324396574,-0.2770003974
O,0,-5.4856054303,-1.0109935754,-0.0185503063
O,0,-4.7962481293,0.9004170036,-0.7675026862
Fe,0,-6.8557889559,0.3728397048,-0.6587037664
O,0,-8.6092270369,-0.8126946186,-0.4949924194
O,0,-7.4241189538,2.3168447852,-1.3024305383
H,0,-6.9984727213,2.5051210083,-2.154516102
H,0,-7.0745445162,2.9775693735,-0.6823459799
H,0,-8.3634103313,-1.7431010773,-0.6255969346
H,0,-9.2113793395,-0.6001683049,-1.2265026411
O,0,-6.8398072651,-0.1824477364,-2.7275883562
O,0,-7.1707203273,1.1168403126,1.3133457706
H,0,-7.7416890982,-0.124303435,-3.0819383335
H,0,-6.3191711318,0.4682232778,-3.2257002295
H,0,-7.870895891,1.7887668404,1.3319430642
H,0,-7.4903333289,0.3899683854,1.8714440918

{3} Fe^{3+} – LA^{-} (S1)

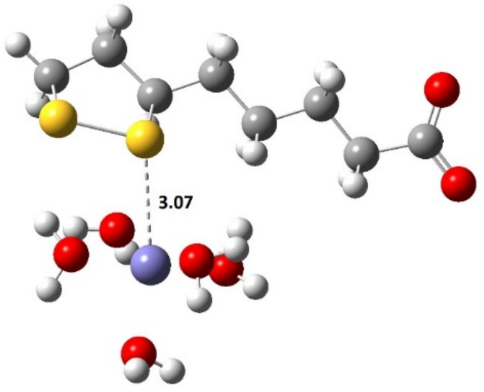

Charge = 2 Multiplicity = 6
C,0,-0.5312711281,-1.6482487009,2.0319142071
H,0,-1.2163493059,-1.2661113879,2.7964867103
H,0,0.1453515387,-2.3801398572,2.4831744944
C,0,0.1895413877,-0.5361253528,1.3085531369
H,0,0.957697647,-0.9392372592,0.6347568825
H,0,0.6857725923,0.1172048581,2.0379886213
C,0,-0.8110954459,0.2916887886,0.5302270511
H,0,-1.52228645,0.7708244106,1.2179722592
S,0,-1.5094283898,-2.573023074,0.7752879239
S,0,-1.8419973037,-0.9178637104,-0.4656284287
C,0,-0.2171860626,1.3127838183,-0.4178362028
H,0,0.4626736399,1.9431456588,0.1750664036
H,0,0.3997052238,0.8055064056,-1.1749394263
C,0,-1.2756196127,2.1827952298,-1.0724262355
H,0,-1.9856759843,1.5495604524,-1.6375945106
H,0,-1.8521652765,2.6778613869,-0.2707193008
C,0,-0.719813734,3.2378455984,-2.0124815567
H,0,-0.1504672584,2.7476506172,-2.8156676414
H,0,-0.00524847,3.8729608915,-1.4687359284
C,0,-1.8301356147,4.0859468811,-2.603678144
H,0,-2.5651683998,3.4427088658,-3.1121768091
H,0,-2.3924209498,4.5851071077,-1.7992853267
C,0,-1.4186365689,5.1642852829,-3.5957032196
O,0,-2.3605876063,5.8429408855,-4.1056613628
O,0,-0.1942699122,5.3295960385,-3.8564668642
Fe,0,-4.755289882,-0.0500385077,-0.0694652606
O,0,-6.8459825501,-0.0856110436,0.0767777014
O,0,-4.7146760049,1.996815978,-0.4977210036
H,0,-5.0875602948,2.1362202706,-1.3839034923
H,0,-3.7967681528,2.3153954865,-0.5476168395
H,0,-7.2335087027,-0.7260528349,-0.5417450978
H,0,-7.1873969264,0.7829761217,-0.1928518839
O,0,-4.5952803225,-0.5390191135,-2.0854143952
O,0,-3.9925862845,0.5519263403,1.7330277921
H,0,-5.495488945,-0.6959316336,-2.417110103
H,0,-4.2511169171,0.2051840525,-2.6077746345
H,0,-4.4883285112,1.3013802935,2.1028978987
H,0,-4.0370198447,-0.1617312601,2.3915076001
O,0,-4.6456860646,-2.0952816863,0.5151460741

```
H,0,-3.9749937133,-2.2965709106,1.1938481798
H,0,-5.497791959,-2.3655723679,0.8947051215
```

{4} Fe^{3+} – LA^- (S2)

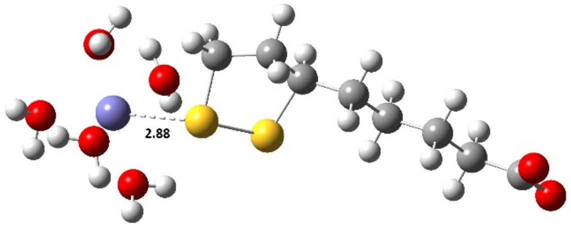


```
Charge = 2 Multiplicity = 6
C,0,-1.4479498436,-0.1308550387,0.0503722703
H,0,-1.8563765061,-0.121309925,1.0674918299
H,0,-1.2885731484,-1.1629637405,-0.277613284
C,0,-0.2212862171,0.7359151294,-0.0863948101
H,0,0.2067292679,0.627017213,-1.0922351611
H,0,0.5373617193,0.4110388663,0.6370715747
C,0,-0.5450105492,2.1983315228,0.1723018257
H,0,-0.6740482302,2.3847180757,1.2463883319
S,0,-2.7009328299,0.5940273794,-1.0780092348
S,0,-2.2276434655,2.567516818,-0.5859785266
C,0,0.490531597,3.1315664932,-0.4249163042
H,0,1.4663913061,2.8266124892,-0.0137754219
H,0,0.5399670876,2.9522691644,-1.5104784019
C,0,0.2704378953,4.6092643007,-0.1529307469
H,0,-0.7162613919,4.9213668087,-0.5362466502
H,0,0.2497560779,4.7875287008,0.9338645572
C,0,1.3458025456,5.4666963778,-0.8025575789
H,0,1.3626298723,5.2617691414,-1.8836070704
H,0,2.3320473496,5.1639519524,-0.4196555482
C,0,1.1322863365,6.9491712145,-0.5665418336
H,0,0.1376145024,7.2512207471,-0.9279200752
H,0,1.1260967415,7.1671687678,0.5120916749
C,0,2.1438377256,7.8893932446,-1.2066558665
O,0,1.9537911319,9.1295430349,-1.021621572
O,0,3.1005378853,7.4055392925,-1.8751266836
Fe,0,-5.1387793397,-0.4007271394,0.0868818666
O,0,-6.9580450424,-1.3663585128,0.4843923246
O,0,-6.0599555101,1.2948257746,-0.8128058999
H,0,-6.0553927501,1.3063979107,-1.7828026858
H,0,-5.7324241427,2.1607435167,-0.520269792
H,0,-6.9503304698,-2.2581798535,0.1005932642
H,0,-7.6846878262,-0.8946022548,0.0463029317
O,0,-4.8214036659,-1.340985655,-1.7548064336
O,0,-4.4753288438,0.7911315427,1.6468528197
H,0,-5.3742351654,-2.1409692617,-1.7791222083
H,0,-5.1068890915,-0.8034377863,-2.5121383084
H,0,-5.0399657228,1.5781639954,1.7272425642
H,0,-4.5634253296,0.3100022666,2.4867962109
O,0,-4.0107066127,-2.0664642361,0.7609437628
H,0,-3.4560230857,-2.5399305694,0.120423902
H,0,-3.4415012413,-1.8632490285,1.5207573566
```

{5} Fe^{3+} – $DHLA^-$ (CO)

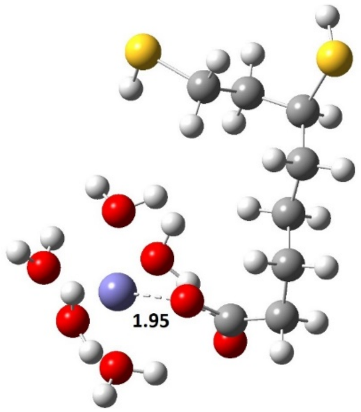


```
Charge = 2 Multiplicity = 6
C,0,0.1962011263,0.2549342917,-0.2166377383
H,0,0.9216026746,-0.0845722846,-0.9699736758
H,0,0.3948543903,-0.3203035362,0.6954408737
C,0,-1.2229128232,0.0192893957,-0.7136851541
H,0,-1.3411019047,-1.0563039919,-0.9054762868
H,0,-1.373469958,0.5238993179,-1.6783614077
C,0,-2.291728731,0.4714278833,0.2717330356
H,0,-2.2428610483,1.566658366,0.3977451238
H,0,-2.0855351473,0.039462967,1.2643400853
C,0,-3.682123023,0.0474562368,-0.1794079691
H,0,-3.6963758124,-1.0503346198,-0.2562742305
C,0,-4.790215453,0.4904774695,0.7683509093
C,0,-5.0222872645,1.9990065673,0.8381202106
H,0,-4.1389329727,2.46643909,1.3031363626
H,0,-5.8607150697,2.1945653021,1.5214164181
```

```
H,0,-4.5448316851,0.1393236422,1.7796315132
C,0,0.5323742675,1.6908725296,0.0697752515
O,0,0.037152082,2.5668697225,-0.7592802913
O,0,1.2596657671,2.0126639766,1.0262841161
C,0,-5.2954930612,2.6650327485,-0.4978762073
H,0,-4.4095497061,2.6669034907,-1.1443801188
H,0,-6.0930012743,2.1436392074,-1.0410532857
H,0,-3.8792696426,0.4260973956,-1.1948195348
S,0,-5.9026921085,4.3865554831,-0.3291487082
H,0,-4.8112392989,4.8634698842,0.3121833754
S,0,-6.3254070771,-0.4147415781,0.2568310304
H,0,-7.1535025912,0.1818850962,1.1425926244
Fe,0,0.1243434943,4.495314233,-0.504167565
O,0,2.140153481,4.5861052884,0.0731824375
H,0,2.225417257,3.7166583221,0.5238543509
O,0,0.591717945,4.8752070037,-2.5104397021
H,0,-0.2044554091,4.7426659554,-3.0504762574
O,0,-1.9739331178,4.4871015944,-0.903758803
H,0,-2.3945930714,5.2498086426,-0.4755165362
O,0,-0.1214434862,6.6156765284,-0.1140181523
H,0,-0.7236514953,6.73004084,0.6374591079
O,0,-0.394701222,4.3146520382,1.6538575828
H,0,0.1620673082,3.5477439079,1.8945661977
H,0,-0.5640526058,7.0346495042,-0.8687722454
H,0,2.7089931568,4.5413413863,-0.712529287
H,0,-1.3062735713,3.9815711845,1.676886729
H,0,1.2352175577,4.2188481225,-2.8235380877
H,0,-2.3786934961,3.6989033051,-0.5044673918
```

{6} Fe^{3+} – $DHLA^{-}$ (COO)

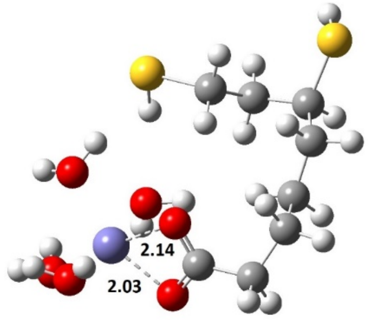


```
Charge = 2 Multiplicity = 6
C,0,3.3725427617,-1.3256103809,0.1119761071
H,0,4.1873889959,-1.7716689345,-0.4760091127
H,0,3.6167901381,-1.5005104732,1.1668836696
C,0,2.0269144564,-1.9230163241,-0.2732237846
H,0,2.0784332535,-3.0148614477,-0.1803989377
H,0,1.8337690288,-1.7098983073,-1.3341884406
C,0,0.8848579768,-1.3686740102,0.5714363462
H,0,1.031540786,-0.285663968,0.6999939819
H,0,0.9192003833,-1.7959755038,1.5844347456
C,0,-0.4758901099,-1.6237530844,-0.0615765793
H,0,-0.7120745471,-2.6962249589,-0.0030721294
C,0,-1.607582919,-0.823795312,0.5757103787
C,0,-1.4204329217,0.6949055769,0.5314529187
H,0,-0.6642331566,0.9739147223,1.282693722
H,0,-2.3521170361,1.1808340969,0.852443364
H,0,-1.7151476311,-1.1234842469,1.6263694102
C,0,3.4328393041,0.1414593352,-0.1323631231
O,0,2.7692338895,0.7180947343,-1.0499318523
C,0,-0.9970060134,1.2363632089,-0.8217880122
H,0,0.0036755503,0.8879260511,-1.1036891583
H,0,-1.6911023017,0.9248899264,-1.6115686262
H,0,-0.4237404839,-1.3815110584,-1.1349188048
S,0,-1.0061645776,3.0732535819,-0.8806585149
H,0,-0.397529601,3.2565317941,0.3136663612
S,0,-3.1625577331,-1.3450588566,-0.2864114051
H,0,-4.0018656198,-0.5680063262,0.4329738352
Fe,0,3.5185987389,2.5912730441,-0.3456070056
O,0,4.16442732,0.9134259204,0.589956635
O,0,4.3137486646,4.3608154771,0.5325753759
H,0,4.6711887306,4.9243919375,-0.1729097343
O,0,1.8122508275,2.8110508171,1.0589622851
H,0,2.1743309253,3.1379203789,1.8983534115
O,0,5.0708746641,2.6302478249,-1.7613004753
H,0,5.101146412,3.4983242257,-2.1955888724
```

O,0,2.2477553914,3.6747865577,-1.6855572075
H,0,2.2868185113,4.6067825354,-1.416001637
H,0,4.8688088874,1.9896092524,-2.4625546127
H,0,3.595591944,4.8717803522,0.9403132985
H,0,1.4863029125,1.9177836369,1.2587345474
H,0,1.3106939781,3.4094922948,-1.5579351777

{7} Fe^{3+} – $DHLA^{-}$ (CO) (5-coord)

Charge = 2 Multiplicity = 6
C,0,0.5044014022,1.2712371769,1.8518028374
H,0,0.9309877455,2.1127691258,1.2921442464
H,0,0.735709898,1.410245977,2.9138461403
C,0,1.0870413124,-0.0441435797,1.3423853375
H,0,2.1800726335,-0.0048293246,1.4400219963
H,0,0.8752201049,-0.1438148589,0.2666875348
C,0,0.542772494,-1.2551343492,2.0850809804
H,0,-0.5576744151,-1.2408442935,2.0526219272
H,0,0.8130544273,-1.1936533999,3.1504231429
C,0,1.0621659656,-2.5577163281,1.493648134
H,0,2.1469271518,-2.6169810436,1.6675683147
C,0,0.3939040379,-3.7987530108,2.0703667477
C,0,-1.105250905,-3.9251410174,1.7994065128
H,0,-1.6360991146,-3.1704276457,2.3967551735
H,0,-1.4362004436,-4.8954047875,2.1954934099
H,0,0.5420801136,-3.8060836008,3.1584804948
C,0,-0.9887352366,1.3230504836,1.6944155729
O,0,-1.4209127392,1.3014854493,0.4600246223
O,0,-1.7443246425,1.3729364316,2.6760308755
C,0,-1.5379918607,-3.8290726423,0.3400683258
H,0,-0.7516301593,-4.1774133144,-0.3399160412
H,0,-2.4078136373,-4.468956499,0.1613548242
H,0,0.9357720915,-2.5469938025,0.3979267997
S,0,-2.1299484553,-2.184214894,-0.2373741943
H,0,-0.9549287899,-1.5169309116,-0.2234590441
S,0,1.3294836966,-5.2535594387,1.4027171147
H,0,0.5810956704,-6.2024871947,2.007043807
Fe,0,-3.1172377233,1.6666522942,-0.3342761543
O,0,-4.6095468186,0.2806475827,-0.7555499762
H,0,-4.4844573063,-0.1484805488,-1.6170841009
O,0,-4.0786421459,1.7238369494,1.5100790147
H,0,-3.3444015569,1.5379132608,2.1569705674
O,0,-3.4557914622,3.6608457837,-0.5783323728
H,0,-2.7367709556,4.0833326922,-1.0767743969
O,0,-2.2798642613,1.3907380272,-2.2694744239
H,0,-2.8975961228,1.0175608213,-2.9176036332
H,0,-3.4973006645,4.1134158697,0.2805753422
H,0,-4.6640323304,-0.436523183,-0.1031716431
H,0,-1.53084646,0.7745495569,-2.2284310346
H,0,-4.7903962687,1.0887622865,1.6769631382

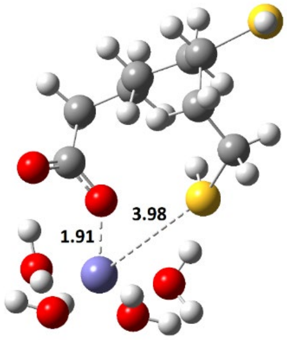

{8} Fe^{3+} – $DHLA^{-}$ (CO) (5-coord)

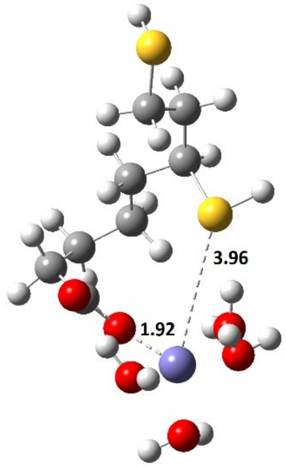


```
Charge =  2 Multiplicity = 6
 C,0,-0.3392671636,2.834562989,0.9814814836
 H,0,-0.9867660477,3.6623193425,1.3048933038
 H,0,0.5298632896,2.8537279978,1.648524173
 C,0,0.0664237924,3.0460609288,-0.4768936164
 H,0,0.7656091326,3.8930510904,-0.4964768859
 H,0,-0.8069233325,3.3563099305,-1.0646920344
 C,0,0.7004105066,1.8327968293,-1.1499869155
 H,0,1.0788159951,2.1261448623,-2.1391511676
 H,0,-0.0855343437,1.0902450334,-1.3422381047
 C,0,1.8302894501,1.1822227776,-0.3621476929
 H,0,1.553491783,1.0605641249,0.696782786
 C,0,2.2529432062,-0.1700140054,-0.9220105976
 C,0,3.604313379,-0.6566813456,-0.410236788
 H,0,4.3679483303,0.0253309179,-0.8165222208
 H,0,3.8193235269,-1.6505406812,-0.8272864092
 H,0,2.2963010662,-0.1119912142,-2.0174786067
 C,0,-1.0993169459,1.575008147,1.2926743573
 O,0,-0.9468070187,0.9902844699,2.3784354901
 O,0,-1.9369434975,1.1663334872,0.3822695278
 C,0,3.7322724208,-0.6987911917,1.1012192112
 H,0,3.6128212516,0.2948203313,1.545495559
 H,0,2.971771506,-1.3518349254,1.5458676624
 H,0,2.7130973405,1.8407641051,-0.3640190863
 S,0,5.3330019564,-1.3960784185,1.6568563461
 H,0,6.1120867029,-0.4820309616,1.0370018134
 S,0,0.90767002,-1.3981392642,-0.5236271274
 H,0,1.4389451496,-2.4327990641,-1.2115864852
 Fe,0,-2.8863131137,-0.4867349564,0.1622942697
 O,0,-4.7534273608,-0.0689451653,-0.479335228
 H,0,-5.1122672108,0.7260621805,-0.0508838751
 O,0,-2.1025634695,-0.6716430067,-1.7963957644
 H,0,-1.1530658072,-0.8968069645,-1.6758886133
 O,0,-2.6350368038,-2.5224993231,0.2019822928
 H,0,-2.0827752259,-2.8471720088,0.9313649316
 O,0,-3.0592432653,-0.5975128242,2.2057683866
 H,0,-2.249036589,-0.1216280407,2.5341270483
 H,0,-2.281396255,-2.9152982873,-0.6119526845
 H,0,-4.7705455763,0.1031272298,-1.4358402292
 H,0,-3.0771723581,-1.4777807516,2.6107914646
 H,0,-2.1100978006,0.1996388253,-2.2250536844
```

{9} Fe^{3+} – $DHLA^{-}$ (S2)

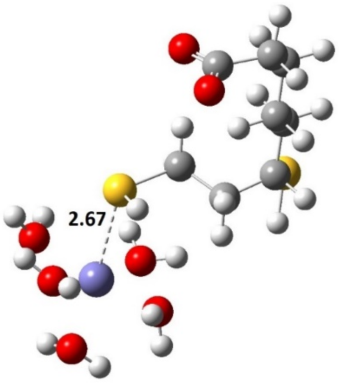


```
Charge = 2 Multiplicity = 6
C,0,3.2167452499,-1.2072021923,-0.6228081115
H,0,3.7909219394,-1.8383861414,-1.3165139659
H,0,3.7291711607,-1.2565749905,0.347089746
C,0,1.7940106933,-1.7326427738,-0.4983491468
H,0,1.8178632872,-2.7894956757,-0.1961042411
H,0,1.3037396494,-1.7015080156,-1.482721116
C,0,0.9646017095,-0.9355800458,0.4980843259
H,0,1.0351048701,0.1335656211,0.2427928556
H,0,1.3926311211,-1.0376677751,1.5083894736
C,0,-0.493287533,-1.3730216075,0.5093744139
H,0,-0.5487800693,-2.4389129697,0.7756017731
C,0,-1.3626593611,-0.5818204402,1.4808301636
C,0,-1.317234043,0.9319801044,1.287638199
H,0,-0.3063598991,1.2756832041,1.5548633843
H,0,-1.997905405,1.4107832544,2.0033888955
H,0,-1.0310781422,-0.7940656136,2.5054634616
C,0,3.3046596221,0.2202438401,-1.1527775506
O,0,2.5670782942,0.5370606904,-2.1313210494
O,0,4.1325761971,1.0031842771,-0.5999583867
```

C,0,-1.6420790001,1.3746270097,-0.1253035333
H,0,-1.0047282803,0.8852673844,-0.8708544256
H,0,-2.6850577378,1.1809958969,-0.4011323337
H,0,-0.9024096493,-1.2939983717,-0.5108919866
S,0,-1.3900326531,3.1690207354,-0.4023796818
H,0,-0.3297607968,3.3334850415,0.4296973989
S,0,-3.0853354741,-1.2432938774,1.3284711322
H,0,-3.6277933892,-0.4638047544,2.2910539246
Fe,0,-3.0905790333,4.6466174916,1.0392990881
O,0,-2.1389686421,3.997564057,2.785226722
H,0,-2.6728051505,4.2984122779,3.5409286725
O,0,-4.3224257511,5.9670934945,2.0884002486
H,0,-5.0608595365,6.2519352549,1.524696859
O,0,-4.3982591188,3.0269349255,1.0735654681
H,0,-4.7424866958,2.7386354309,0.2119771606
O,0,-1.5724060044,6.0410974235,0.6694155329
H,0,-1.3526246285,6.3882378181,-0.2096357198
O,0,-3.7635285694,5.2448277916,-0.8832892693
H,0,-3.126054133,5.5219589823,-1.5610399329
H,0,-4.725962722,5.5093721449,2.8447547816
H,0,-4.3324367048,4.5743270415,-1.2966658106
H,0,-1.2594637319,4.3953174929,2.9091491643
H,0,-0.7492639939,5.9825071279,1.1800703391
H,0,-4.1037467948,2.2304450402,1.5460408571

{10} Fe^{3+} – $DHLA^{-}$ (S1)

Charge = 2 Multiplicity = 6
C,0,3.739095851,-0.8033441355,1.3970116169
H,0,4.0279281557,-1.863240281,1.4077200287
H,0,3.9932541171,-0.3818493435,2.3781298709
C,0,2.2392009932,-0.6835081229,1.1536295932
H,0,1.7009115334,-1.2354335461,1.9383783713
H,0,1.9758440214,-1.1662826537,0.1994779577
C,0,1.7558910069,0.7600305784,1.1343462896
H,0,2.2803759517,1.3144185555,0.3398798287
H,0,2.016918584,1.2537656781,2.0843882111
C,0,0.2540384929,0.8290958111,0.9094824637
H,0,-0.2493443453,0.2129539839,1.6720249263
C,0,-0.3256938953,2.2320029432,0.9665975803
C,0,0.2248750631,3.2137192681,-0.062152429
H,0,1.2865721651,3.375231629,0.1748168478
H,0,-0.2685635148,4.1865907187,0.0633313607
H,0,-0.1679770079,2.6609746341,1.9649892244
C,0,4.5638542218,-0.107733925,0.3222518865
O,0,4.3875071538,-0.4747736864,-0.8771603061
O,0,5.3756306285,0.7944807831,0.6812428577
C,0,0.0980594864,2.7603356108,-1.5060235615
H,0,0.6206022504,1.8141702039,-1.6856246395
H,0,-0.9519727811,2.6154112042,-1.7948953215
H,0,0.0118225011,0.3827600248,-0.066823511
S,0,0.7313874106,4.0088086498,-2.6880155885
H,0,1.9937797443,4.0235011627,-2.2056375598
S,0,-2.1747803387,2.1599930962,0.7818016415
H,0,-2.418861087,1.6299872897,2.0033612482
Fe,0,-3.1835443169,-0.5733920054,-0.4644558906
O,0,-4.431061092,-2.1744664057,-1.0231458101
H,0,-4.4026886131,-2.8239751691,-0.3004936643
O,0,-1.4773001582,-1.7715612953,-0.3348329097
H,0,-1.0328167269,-1.6833186924,0.525724436
O,0,-2.5548704659,0.2190363693,-2.2479912775
H,0,-2.9597682735,1.0614732275,-2.5158908759
O,0,-3.5730737198,-0.921399717,1.5960640235
H,0,-4.1154800168,-0.2584787362,2.0520891977
O,0,-4.7982283481,0.7626442599,-0.5477742734
H,0,-5.2948937257,0.8814921359,0.278253767

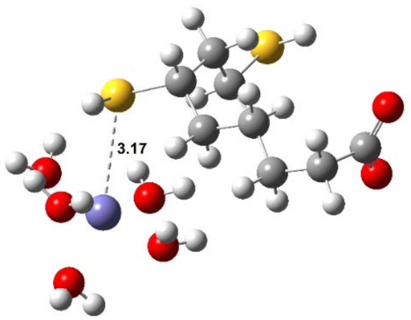

```
H,0,-4.0500911254,-2.6223374624,-1.7968811413
H,0,-0.8151363657,-1.5327673693,-1.0053043096
H,0,-4.6706083574,1.6397318013,-0.9416180315
H,0,-1.5914427644,0.3532772181,-2.2828103564
H,0,-2.7678821824,-1.0457812905,2.1253294679
```

{11} Fe^{3+} – $DHLA^{-}$ (S1, S2 *cis*)

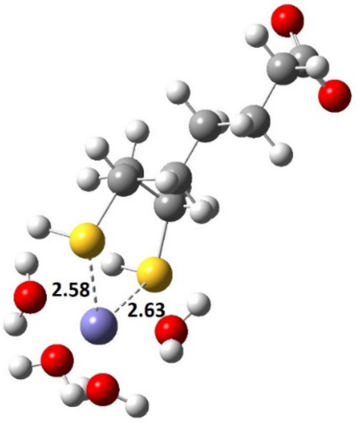


```
Charge = 2 Multiplicity = 6
C,0,0.0439015708,-6.3243445162,4.0804866082
H,0,-0.1918160275,-6.4064059022,5.1501539135
H,0,0.5404623988,-7.2552397367,3.7776575177
C,0,0.9770709572,-5.1398474575,3.8537617771
H,0,1.8875835734,-5.2771223759,4.4543261022
H,0,0.5001507966,-4.2167020066,4.2152628486
C,0,1.3598007923,-4.9722032843,2.3909569928
H,0,0.4505243668,-4.8203339676,1.7885844766
H,0,1.8241391723,-5.9011429124,2.021728136
C,0,2.3246329196,-3.8116796256,2.1840643089
H,0,3.1829699892,-3.9382589444,2.8587267321
C,0,2.8396384141,-3.7414568297,0.7540051431
C,0,1.786075485,-3.5931506974,-0.3329603178
H,0,1.2800563713,-4.5674266112,-0.3942948266
H,0,2.2589148506,-3.4519496499,-1.3148477047
H,0,3.4115084892,-4.6540097595,0.5390536492
C,0,-1.2694683036,-6.1822913415,3.3222395477
O,0,-1.9742505035,-5.1595995873,3.5694362813
O,0,-1.5817453543,-7.0817685736,2.4880700749
C,0,0.6929142898,-2.5701985516,-0.1037527675
H,0,-0.1801049946,-2.8038224342,-0.7200178258
H,0,0.3509982193,-2.5315078598,0.9356661936
H,0,1.8392162994,-2.8660303767,2.4744424822
S,0,1.0731244306,-0.8220628885,-0.5294010752
H,0,1.3114667684,-1.0191920809,-1.8498468772
S,0,4.186030313,-2.4493596849,0.7109316924
H,0,4.7157016207,-2.8060095608,-0.4845068127
Fe,0,3.5243320599,-0.0537827868,0.0293084672
O,0,2.7180631698,0.0906553279,1.8795407446
H,0,3.3391056123,0.4099769992,2.5569700707
O,0,5.4992471913,0.3740460474,0.6383299071
H,0,6.0599213402,0.5049750562,-0.1446738981
O,0,2.9675780296,1.7675094221,-0.7360503388
H,0,2.809944206,1.8639804355,-1.6893583402
O,0,4.1055015937,-0.7526663616,-1.8485725838
H,0,3.7076467033,-1.4937094765,-2.335612439
H,0,5.5086886731,1.2189480268,1.1182798475
H,0,4.1600044224,-0.0105598532,-2.4754212623
H,0,2.1006245129,-0.5156227965,2.32225354
H,0,2.2939742906,2.2879696666,-0.2691577355
```

{12} Fe^{3+} – $DHLA^{2-}$ (CO, S1 *cis*)

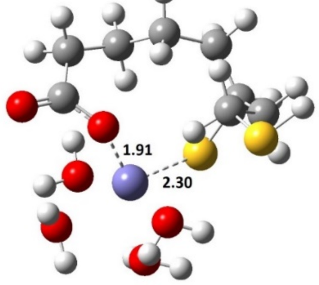


```
Charge =  1 Multiplicity = 6
 C,0,-0.3170764198,-3.4236037887,0.7287090875
 H,0,-0.2357842229,-3.4768151555,1.8226317611
 H,0,0.0225579302,-4.4053454099,0.3638403758
 C,0,0.5521640479,-2.3135662299,0.1644914323
 H,0,1.5478492756,-2.3787212483,0.6247485566
 H,0,0.1509808892,-1.3373061476,0.4642906296
 C,0,0.6979638455,-2.35851017,-1.3544063164
 H,0,-0.2440150801,-2.6877239898,-1.8033715815
 H,0,1.4368461219,-3.1309981386,-1.6116107611
 C,0,1.1480560514,-1.0310051963,-1.9644422453
 H,0,1.6902690224,-1.2268605882,-2.9029512612
 C,0,0.1102066316,0.0321817602,-2.3403525777
 C,0,-0.6965960263,-0.2306877957,-3.615765388
```

```
H,0,0.0217354473,-0.2113022501,-4.4515960511
H,0,-1.3757955638,0.6189949472,-3.7746496865
H,0,0.6922403448,0.9367593154,-2.5616612191
C,0,-1.7961731215,-3.3562412051,0.4223156431
O,0,-2.2101537409,-2.443850751,-0.4111456391
O,0,-2.569574428,-4.175276708,0.9344414466
C,0,-1.4875086598,-1.522470863,-3.7027024168
H,0,-0.8311054858,-2.3964020836,-3.75776675
H,0,-2.1549760269,-1.6594190891,-2.8439351796
H,0,1.8831711685,-0.5685664789,-1.2884228308
S,0,-2.6018832149,-1.5576674836,-5.1605868894
H,0,-1.6302923881,-1.3988837918,-6.0869137745
S,0,-0.9668378213,0.6956690374,-0.963092286
Fe,0,-2.6834419599,-0.611222727,-0.1690386361
O,0,-4.4815816935,-1.4230140341,1.0621900262
O,0,-3.6728692785,1.4094377788,0.4325424033
O,0,-2.0941337118,-0.4448366305,1.902260151
O,0,-4.0737623689,-0.4861247279,-1.8158762974
H,0,-1.2164099396,-0.8023005595,2.1047783681
H,0,-2.7266257824,-1.0100777694,2.3755350411
H,0,-4.7040741499,-2.2736812111,0.6559508416
H,0,-5.2459385582,-0.8519722877,0.8972297439
H,0,-3.9286002229,0.2919356122,-2.3768542127
H,0,-4.9598297959,-0.3690265787,-1.43874403
H,0,-3.5793756594,2.0803111232,-0.2593482617
H,0,-3.2411724052,1.7847198243,1.2140576641
```

{13} Fe^{3+} – $DHLA^{2-}$ (CO)

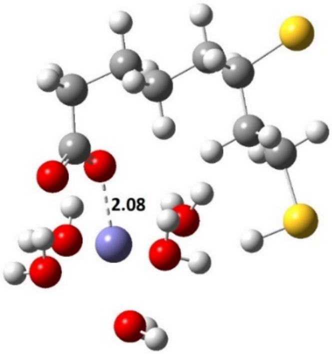


```
Charge = 1 Multiplicity = 6
C,0,-0.1269681674,-3.1921332826,-0.422557174
H,0,-0.7237233081,-3.9474670017,0.1058107845
H,0,0.2592125514,-3.650988806,-1.340489368
C,0,1.0274638064,-2.7314314963,0.4614915071
H,0,1.6626355141,-3.598885253,0.6886941519
H,0,0.6396309516,-2.3683341025,1.4245291371
C,0,1.8690066976,-1.644443521,-0.1908452572
H,0,1.2457747947,-0.7503401113,-0.3614004754
H,0,2.200990028,-1.9824868453,-1.1868102871
C,0,3.0806426879,-1.2813699302,0.6544295329
H,0,3.6344613392,-2.1981325867,0.9012178836
C,0,4.0422267827,-0.3168014675,-0.0382233634
C,0,3.4178178871,0.9917988236,-0.5251725794
H,0,2.6607339083,0.7630574511,-1.2910997317
H,0,4.1931705713,1.5921175433,-1.0178312839
H,0,4.4615210549,-0.8291111925,-0.924792636
C,0,-1.0507494423,-2.0576646762,-0.7995078728
O,0,-1.6077526892,-1.4315761903,0.1661147862
O,0,-1.2239145577,-1.7828747834,-2.0149075228
C,0,2.778331865,1.8030600129,0.5858610766
H,0,1.9253323242,1.2826101758,1.037433974
H,0,3.4997327359,2.0058612505,1.3897836583
H,0,2.7499501846,-0.8548095091,1.6144356679
S,0,2.2276648952,3.4603793302,0.0288815823
H,0,1.2988084934,3.0116417709,-0.8465582573
S,0,5.5298576891,-0.0276323615,0.9687852057
Fe,0,-2.8722822517,0.215159825,0.0010333024
O,0,-4.1662711717,1.9819196921,-0.1514572449
O,0,-2.3445961458,0.7899104066,2.0977100179
O,0,-3.4412852679,-0.2592908017,-2.1057630805
O,0,-4.6096351471,-0.9203292895,0.8067736669
O,0,-1.1943278518,1.5339615098,-0.7834590028
H,0,-4.3109675569,-1.5114989335,1.5123194936
H,0,-4.9798677082,-1.5044147494,0.1301302626
H,0,-2.6637237196,-0.8610038509,-2.2315365039
H,0,-4.2211706261,-0.8298027692,-2.1101984088
```

H,0,-3.6216521838,2.7511797971,-0.3738388891
H,0,-4.5309774684,2.1796190615,0.7234225231
H,0,-0.3783118327,1.0107666567,-0.7605027392
H,0,-1.0618602503,2.2286240665,-0.1224349244
H,0,-1.3935955823,0.6623850371,2.2246719394
H,0,-2.7626598335,0.1733561003,2.7151404487

{14} Fe^{3+} – $DHLA^{2-}$ (COO)

Charge = 1 Multiplicity = 6
C,0,0.7607488326,-2.9467770173,-0.0779310387
H,0,1.2645006098,-3.7187898629,-0.6738144429
H,0,0.8042846108,-3.2506652575,0.9746704765
C,0,-0.6899924036,-2.8028079665,-0.5292138687
H,0,-1.1940199377,-3.7722862631,-0.4148783325
H,0,-0.7201942949,-2.5585340254,-1.6013452679
C,0,-1.4431271166,-1.7408225433,0.2582204545
H,0,-0.93182313,-0.7697357691,0.1483565229
H,0,-1.4100128784,-1.9867794538,1.3326870308
C,0,-2.8899905998,-1.6180173983,-0.1960705528
H,0,-3.3546862156,-2.6141752475,-0.200404806
C,0,-3.7336446037,-0.7029958709,0.6911828685
C,0,-3.1694948316,0.7030960525,0.9017384867
H,0,-2.2065725169,0.6250521402,1.429813323
H,0,-3.8461081572,1.2619097971,1.5607333383
H,0,-3.8181629147,-1.1752285242,1.6883114548
C,0,1.522803189,-1.6605209533,-0.2543010668
O,0,1.6438168083,-1.1477435214,-1.3984199508
O,0,2.0304544307,-1.0934564816,0.776179587
C,0,-2.9715644988,1.4705426096,-0.3916283797
H,0,-2.2248964111,0.9972689002,-1.039843128
H,0,-3.9096309444,1.5303014816,-0.961089456
H,0,-2.9202203587,-1.2605949578,-1.2371618696
S,0,-2.4791754703,3.2144555163,-0.1156024141
H,0,-1.2939909758,2.9310827713,0.4726942121
S,0,-5.4677146518,-0.6659281125,0.1416186434
Fe,0,2.8916385694,0.5641443133,-0.1846113137
O,0,3.1488745993,1.78200396,-1.9378272062
O,0,3.8960113043,1.6328650478,1.3898923441
O,0,4.7627746374,-0.5386604158,-0.6322486866
O,0,0.9854142336,1.7424433064,0.1698488634
H,0,4.5384123701,-1.4679199287,-0.7832385782
H,0,5.341342734,-0.540950856,0.1432645528
H,0,4.6166851816,1.1256066401,1.789139474
H,0,3.3173474459,1.8852167756,2.1231816647
H,0,2.2996503859,1.861205128,-2.3968987237
H,0,3.739322287,1.3432993503,-2.5677080452
H,0,0.9197748565,2.4334190883,-0.5053322228
H,0,1.0515184754,2.2246426182,1.0065335332

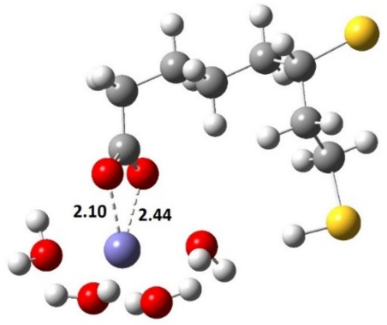

{15} Fe^{3+} – $DHLA^{2-}$ (S1)

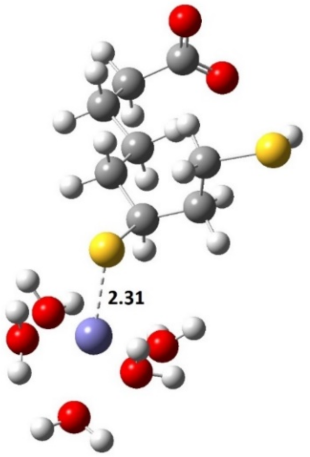


```
Charge = 1 Multiplicity = 6
C,0,4.2600794061,-1.7251838611,1.8121014415
H,0,5.2080194098,-2.0365449659,1.352445946
H,0,3.9854990119,-2.4850553982,2.5552295159
C,0,3.1762324941,-1.6329144087,0.742160956
H,0,3.0619397448,-2.6157236452,0.2623480063
H,0,3.4899578519,-0.9347101902,-0.0479633608
C,0,1.8373306454,-1.1885281683,1.3114441539
H,0,1.9428523195,-0.1807082225,1.7435813536
H,0,1.5580463228,-1.8525624221,2.1470148677
C,0,0.7228927946,-1.1917034432,0.2722365197
H,0,0.6853675552,-2.1791837488,-0.2128330132
C,0,-0.6396125251,-0.9107946925,0.8943677326
C,0,-0.7560217925,0.4173568048,1.6423531075
H,0,-0.1029384661,0.3640959826,2.5268880151
H,0,-1.7815191328,0.5289114552,2.0228015127
H,0,-0.8511354739,-1.7154354271,1.6099678162
C,0,4.4971464626,-0.3961280091,2.5166119218
O,0,4.8419691498,0.5891055157,1.7999307681
O,0,4.3343951531,-0.3458893881,3.7713796571
C,0,-0.3833985162,1.6348452316,0.817292448
H,0,0.6491087441,1.5805220654,0.4557223409
H,0,-1.0328716682,1.7354803381,-0.0609457785
H,0,0.9439202468,-0.4635563729,-0.5233208149
S,0,-0.5920609706,3.2049226617,1.7387936166
H,0,0.3175058698,2.925706741,2.6987280339
S,0,-1.9628237118,-0.9953160801,-0.4151545631
Fe,0,-3.3571883261,-2.6560956175,0.368172872
O,0,-4.6456512842,-4.3438189438,0.9769761426
O,0,-5.134426577,-1.3954913513,0.4683340165
O,0,-1.7755208454,-4.1849174131,0.386494727
O,0,-3.1059739931,-2.3748749611,2.4556487538
O,0,-3.8743377001,-3.1403843542,-1.6640436622
H,0,-2.9923260839,-1.4528368729,2.7354901915
H,0,-2.3752326563,-2.8714306501,2.8545594939
H,0,-1.1417262819,-4.0447152272,-0.333686278
H,0,-1.2498504486,-4.1179698282,1.1990723153
H,0,-5.0549246404,-4.7378245428,0.1920578267
H,0,-5.3836276159,-3.9852466733,1.4924955292
H,0,-3.6705965932,-2.5008332841,-2.3628543018
H,0,-4.8235609957,-3.3250701993,-1.7414019408
H,0,-5.0913716458,-0.628569053,-0.123132778
H,0,-5.1940451876,-1.0221643101,1.3617287715
```

{16} Fe^{3+} – $DHLA^{2-}$ (CO, S2 *cis*)

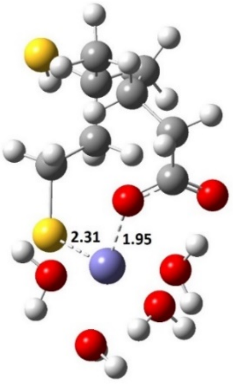


```
Charge =  1 Multiplicity = 6
 C,0,1.3912613752,-1.407933638,0.3160356773
 H,0,1.2529217533,-1.9887908488,1.2382627589
 H,0,2.0181944298,-1.9991699427,-0.3615469248
 C,0,2.0345342033,-0.0607178264,0.6342144956
 H,0,2.9486937853,-0.2230506416,1.2187904902
 H,0,1.3542393999,0.5073008906,1.2832837553
 C,0,2.3414415866,0.7419160201,-0.633447592
 H,0,1.6423730264,0.4341576506,-1.4258509563
 H,0,3.3323346641,0.4580419907,-1.0104082904
 C,0,2.2601307363,2.2669959792,-0.4648464442
 H,0,3.2539782935,2.7190941004,-0.579504837
 C,0,1.3175037149,2.9147496883,-1.478244451
 C,0,-0.1423436466,2.4628291482,-1.3959101733
 H,0,-0.1962843085,1.4054488684,-1.6968582648
 H,0,-0.724450777,3.0172323151,-2.1459063138
 H,0,1.6825439096,2.6796952191,-2.4872249178
 C,0,0.0275132073,-1.2337593684,-0.3089687195
```

O,0,-0.7737192025,-0.4672148451,0.3553040625
O,0,-0.2784379591,-1.7851744053,-1.383130084
C,0,-0.786717745,2.6241258237,-0.0298689512
H,0,-0.2935556101,2.0026288272,0.7254123603
H,0,-0.7365017138,3.6677601129,0.3031878661
H,0,1.9298121407,2.5204470707,0.5539325018
S,0,-2.5734242837,2.189459001,-0.0471240075
S,0,1.4844673386,4.7496743215,-1.2808627892
H,0,0.6229803562,5.0783734659,-2.2687012764
Fe,0,-2.6820526724,-0.106899331,0.1873044864
O,0,-4.8763078056,-0.0157050274,0.0487284888
O,0,-3.0516156555,-2.3659272365,0.495911494
O,0,-2.710563869,-0.6266945017,-1.9650135193
O,0,-2.8658300032,-0.0859826151,2.3435649459
H,0,-5.1773858689,0.4241704519,-0.7613924466
H,0,-5.1948674568,-0.9284527699,-0.0307661465
H,0,-3.6225279988,-2.4742080047,1.2706280005
H,0,-3.5661439178,-2.7179373457,-0.2447870925
H,0,-1.8705962953,-1.1493643994,-1.9700360446
H,0,-3.4202407401,-1.2715050075,-2.1015213546
H,0,-2.4490295012,0.6994299159,2.7298039575
H,0,-3.8056346004,-0.0076560261,2.571363366

{17} Fe^{3+} – $DHLA^{2-}$ (COO, S2)

Charge = 1 Multiplicity = 6

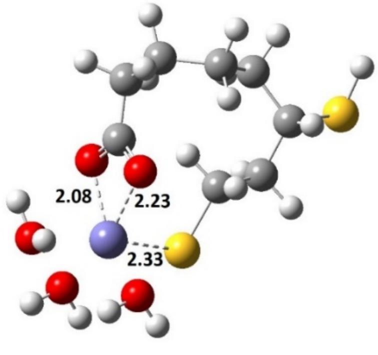

C,0,-0.2448196632,2.8344955526,0.8891786207
H,0,-0.4740698921,3.5507137735,0.0906109957
H,0,-0.4431312717,3.3191115664,1.851916983
C,0,1.2279079312,2.403078792,0.7858131187
H,0,1.8260841652,3.2775648192,0.5037721206
H,0,1.3269295688,1.6968922851,-0.0509585046
C,0,1.7896301463,1.7878804391,2.0756209594
H,0,0.9744461433,1.3409427683,2.6592847023
H,0,2.1685487063,2.5973661813,2.7128830016
C,0,2.9094221269,0.7485734889,1.8481796646
H,0,3.8579492895,1.1597118199,2.2147560065
C,0,2.6808917317,-0.6088411875,2.5226929221
C,0,1.7096073622,-1.5430393428,1.7916115342
H,0,1.6259125607,-2.4883572027,2.3455645512
H,0,2.1188643483,-1.7813982085,0.797429914
H,0,2.3250698825,-0.4544217944,3.5512815954
C,0,-1.1808464986,1.6759876187,0.7177546882
O,0,-1.2453646839,1.0748534735,-0.4127632225
O,0,-1.896329103,1.2221413304,1.6589521772
C,0,0.3445032956,-0.9063834856,1.6356781369
H,0,-0.0716705042,-0.6262935597,2.6104436332
H,0,0.4436320061,-0.009194333,1.0260327071
H,0,3.0571790314,0.5700216768,0.7709492081
S,0,-0.8858401567,-1.9567915935,0.7770844505
S,0,4.2636247026,-1.5631550538,2.6360071973
Fe,0,-2.5419663441,-0.4004425726,0.2697551737
O,0,-3.0271161117,-1.5330207271,-1.4466807157
O,0,-3.8685536288,-1.1380092437,1.7356380019
O,0,-4.3111448197,0.950749024,-0.3580649462
H,0,-3.8865171298,1.7599867596,-0.6794142456
H,0,-4.7536883378,1.2128481081,0.4627729203
H,0,-4.7965473092,-0.9368182083,1.5382573236
H,0,-3.8278510753,-2.0956040979,1.878346435
H,0,-2.9593254131,-2.4983762785,-1.39537089
H,0,-3.9347417367,-1.34739436,-1.7351768071
H,0,4.9562343611,-0.6258591578,3.3188436587

{18} Fe^{3+} – $DHLA^{2-}$ (S2)

```
Charge =  1 Multiplicity = 6
 C,0,3.1898891506,-1.8214839505,-0.0470184537
 H,0,3.8844521393,-2.4425235103,-0.6311660046
 H,0,3.257923436,-2.1652276049,0.9936886312
 C,0,1.7712369492,-1.9942740488,-0.5701139735
 H,0,1.5149384062,-3.0631661511,-0.5937374495
 H,0,1.718453522,-1.6397398871,-1.6102220373
 C,0,0.7495031026,-1.243472666,0.2719060235
 H,0,1.090208714,-0.2043245038,0.4035763149
 H,0,0.7083728718,-1.6809222029,1.2826577039
 C,0,-0.6383290431,-1.2557845934,-0.3530473438
 H,0,-0.9471329467,-2.2973784139,-0.5283842612
 C,0,-1.6958135008,-0.5695983825,0.5047293412
 C,0,-1.3517705908,0.8602270163,0.9136440087
 H,0,-0.4886612977,0.8173404488,1.5942270031
 H,0,-2.1780445575,1.2770288418,1.5081088586
 H,0,-1.8357738633,-1.1538362415,1.4239055956
 C,0,3.711175112,-0.3904508777,-0.1279153684
 O,0,3.4810530906,0.2657052018,-1.1855270144
 O,0,4.3731016753,0.0523829563,0.8567186215
 C,0,-1.0259205749,1.7851323294,-0.2468923451
 H,0,-0.1872903263,1.3988814474,-0.8387596222
 H,0,-1.8792595881,1.9061596438,-0.9242604277
 H,0,-0.592084409,-0.7791120483,-1.3455854775
 S,0,-0.4752116067,3.4292289647,0.3504474079
 S,0,-3.2962986598,-0.6591783968,-0.4256524974
 H,0,-4.0340404419,-0.0030002973,0.4979101319
 Fe,0,-2.315164424,4.7959169374,0.3461353078
 O,0,-4.001212307,6.4148274643,0.1532379688
 O,0,-3.8774268554,3.3853961767,0.2451141665
 O,0,-0.9621109394,6.4657333019,0.5540554675
 O,0,-2.5988860446,4.9926168698,2.4687173619
 O,0,-2.3043076014,5.0069925588,-1.7852958084
 H,0,-4.7231969855,6.0817561589,0.7066015002
 H,0,-3.6586359708,7.1831078374,0.6330992041
 H,0,-0.9470814023,6.9695200231,-0.274732439
 H,0,-1.3120629097,7.0746461172,1.2230345378
 H,0,-1.7730291727,5.0521387538,2.9731625244
 H,0,-3.0744580964,5.8188155637,2.6493284363
 H,0,-3.932214153,2.9304341136,-0.6099521338
 H,0,-3.8445925039,2.6892605702,0.9201413828
 H,0,-2.0899987075,4.1957390839,-2.2710206916
 H,0,-3.1886336395,5.2685155358,-2.087047241
```

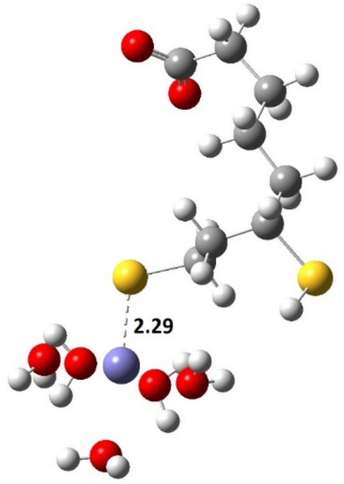

{19} Fe^{3+} – $DHLA^{2-}$ (CO)

```
Charge = 1 Multiplicity = 6
C,0,-0.3229578505,3.2282075485,0.1518814839
H,0,-0.8632741524,3.9337722651,-0.4940204041
H,0,-0.1447490478,3.731139327,1.1097518468
C,0,0.9964914766,2.8284338607,-0.4982672912
H,0,1.6126776309,3.7280449288,-0.6338242961
H,0,0.8092166783,2.4237644327,-1.5034632733
C,0,1.7618123389,1.8043768847,0.3257085573
H,0,1.1365472799,0.905957678,0.4590718851
H,0,1.9450677772,2.2041923071,1.3365198913
C,0,3.0842620045,1.4208457916,-0.3196458769
H,0,3.680930992,2.3318271161,-0.4772197907
C,0,3.8975524047,0.4400038751,0.5161281159
C,0,3.1911268848,-0.8779188544,0.8264737671
H,0,2.3538692495,-0.6716074772,1.5108518274
H,0,3.8802788673,-1.5306580695,1.3803186555
H,0,4.1533009591,0.9219769459,1.4689468615
C,0,-1.2354267662,2.0495935992,0.3933275452
```

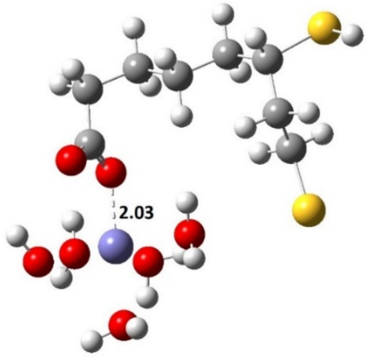

```
O,0,-1.4334711056,1.2612973696,-0.5975594208
O,0,-1.7577133609,1.8844487548,1.5218949113
C,0,2.668592066,-1.6151112386,-0.3933779795
H,0,1.8871989768,-1.0464697036,-0.9216788792
H,0,3.4643828211,-1.7742218611,-1.1388666269
H,0,2.8968107469,1.0008098462,-1.3210431069
S,0,1.9626952328,-3.2371891169,-0.0430186012
S,0,5.4971285328,0.1680496877,-0.3797180219
H,0,6.047141658,-0.6446999778,0.549099749
Fe,0,-2.620289341,-0.3555893167,-0.2750286174
O,0,-0.8327312299,-1.5867525889,0.3702173424
O,0,-4.4892289868,0.8027375813,-0.4820266768
H,0,-4.5900310055,1.0785330234,-1.4041847336
H,0,-4.3919617319,1.6266479957,0.0173616274
H,0,-0.2542231975,-0.9798424711,0.8568243395
H,0,-1.0897837985,-2.2572968872,1.0193840804
O,0,-2.5057528267,-0.8994908094,-2.4203254139
H,0,-3.1927947358,-1.5513861317,-2.6191010383
H,0,-1.6712138703,-1.3612906116,-2.5849683254
O,0,-3.2260981995,-0.2231346916,2.5874363136
H,0,-2.7407854126,0.5235091684,2.1719574263
H,0,-3.9972610246,-0.3637089141,2.0228007029
O,0,-3.8202465626,-2.1423059688,0.102246956
H,0,-4.7409010041,-1.8996000296,0.279949898
H,0,-3.5117503672,-2.5762942677,0.9117225904
```

{20} Fe^{3+} – $DHLA^{2-}$ (COO)

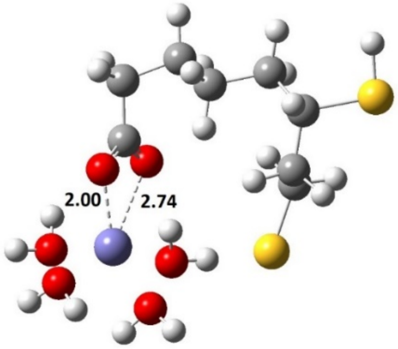


```
Charge = 1 Multiplicity = 6
C,0,0.5316678887,-3.1559872758,-0.3480563912
H,0,0.9339862257,-3.8470051061,-1.0991154379
H,0,0.8026703028,-3.533405806,0.6447872483
C,0,-0.9867927368,-3.0487711635,-0.4883246973
H,0,-1.439137898,-4.0379836113,-0.3390579012
H,0,-1.2284316971,-2.7431839901,-1.5173692103
C,0,-1.5772639061,-2.0336626172,0.4807997048
H,0,-0.9748437553,-1.114003687,0.4187176545
H,0,-1.4709939103,-2.3873616614,1.517762857
C,0,-3.0333828982,-1.7004054338,0.1748558208
H,0,-3.6849190135,-2.5248516324,0.4992291237
C,0,-3.5050569626,-0.4037968237,0.8236955716
C,0,-2.613121241,0.803917176,0.523171076
H,0,-1.6948665112,0.6972945004,1.1180197699
H,0,-3.0956915778,1.7209538063,0.8897982052
H,0,-3.5385654905,-0.5274202455,1.914079836
C,0,1.1676724837,-1.8050221836,-0.5589885412
O,0,1.0593598991,-1.2070639167,-1.6513695198
O,0,1.7893049265,-1.2780293053,0.4431770423
C,0,-2.2396994582,0.9768823515,-0.9420361624
H,0,-1.8333261669,0.04498085,-1.3658819781
H,0,-3.1129085228,1.2298504438,-1.5581574739
H,0,-3.1665821939,-1.6225443858,-0.9151123499
S,0,-0.9496450456,2.2228582647,-1.2274596752
S,0,-5.2301695218,0.010625096,0.2871375898
Fe,0,2.4511587641,0.5343669725,-0.0645957313
O,0,2.467128368,2.1883321969,-1.3872194475
O,0,3.7970021468,0.9445751372,1.5483912835
O,0,4.1161480788,-0.1937431642,-1.2475887022
O,0,1.0283468105,1.6413583387,1.2354308806
H,0,3.8288803027,-0.9837590917,-1.7295734291
H,0,4.8187779069,-0.4976588128,-0.6544867265
H,0,4.7151855373,1.0091446583,1.2472880045
H,0,3.6074026819,1.7780209112,2.0036367251
H,0,2.0895175984,2.9837969118,-0.9835116302
H,0,3.3726191688,2.4243100551,-1.6404411578
H,0,0.6251011469,0.9980429114,1.8375181612
```

```
H,0,0.3063151102,1.9036165442,0.6179515583
H,0,-5.7585114102,-1.2067416723,0.5430156697
```

{21} $[Fe(H_2O)_6]^{3+}$

```
Charge = 3 Multiplicity = 6
Fe,0,0.5156495331,-0.4652565018,-0.0067038937
O,0,0.5011135819,1.632934638,0.0409183841
H,0,1.2539744918,1.9758185732,-0.4686452308
O,0,-1.5966101553,-0.3794419926,0.1374980964
H,0,-1.9790310041,-1.1943569914,-0.2259929348
O,0,2.6311719894,-0.5101741485,-0.1913171651
H,0,3.0155663028,-1.0386232771,0.5264919037
O,0,0.6661521256,-0.5512030619,2.10762618
H,0,0.1213757493,-1.2795288459,2.4479035131
O,0,0.4644686026,-2.5685365699,-0.0643211395
H,0,0.841165508,-2.9022982007,-0.8947001915
O,0,0.3675868376,-0.3797628384,-2.1229154096
H,0,-0.5586327545,-0.1951785029,-2.3525237547
H,0,0.6202538578,1.9691199603,0.9443381601
H,0,0.5737311106,-1.236144216,-2.5310861414
H,0,3.0066123641,0.3805022295,-0.1040102
H,0,1.0105049793,-2.941396156,0.647320241
H,0,0.3216870749,0.2550153773,2.5235640196
H,0,-1.936103195,0.3448415248,-0.4134464368
```

{22} H_2O

```
Charge = 0 Multiplicity = 1
O,0,-0.2196912223,0.8679303539,0.
H,0,0.7444246332,0.9120185665,0.
H,0,-0.4999603273,1.79146291,0.
```

{23} $(H_2O)_2$

```
Charge = 0 Multiplicity = 1
O,0,1.4795281026,-0.0835851456,-0.048466566
H,0,0.5026234757,-0.0338567567,-0.0333344188
H,0,1.7732405663,0.6620366705,0.4880084983
O,0,-1.3467786441,-0.0462944951,-0.0911704095
H,0,-1.7067295094,-0.4895809123,0.6879158933
H,0,-1.7196809911,0.8441736392,-0.0581969972
```

{24} $(H_2O)_3$

```
Charge = 0 Multiplicity = 1
O,0,0.0000485316,1.1143949542,-0.0000250753
H,0,0.7772375014,0.5238992418,-0.0054637797
H,0,-0.7773589256,0.5241841823,0.0056499833
O,0,2.313155996,-0.5440610132,-0.0075966743
H,0,2.9346421251,-0.2804065359,-0.6984634379
H,0,2.8015332832,-0.4256745495,0.8174345769
O,0,-2.31315344,-0.5438404332,0.0076305017
H,0,-2.8011983313,-0.4258816923,-0.817658597
H,0,-2.9349657404,-0.2799631543,0.6981185022
```

{25} LA^-

```
Charge = -1 Multiplicity = 1
C,0,4.0542189454,0.9611987541,0.1499767545
H,0,4.2519961656,1.0260919833,1.2246944321
H,0,4.8452094922,1.4901401279,-0.3897237941
C,0,2.6775230343,1.4902286262,-0.1866490406
H,0,2.5806508062,1.6607283502,-1.2679475664
H,0,2.5220932246,2.4518945614,0.3220753138
C,0,1.6132902651,0.5059543442,0.2619296304
H,0,1.6720184523,0.3584959873,1.3509004231
```

S,0,4.1496239135,-0.8215778912,-0.3349023464
S,0,2.0467351609,-1.1059392376,-0.5379017607
C,0,0.2072377874,0.9138372566,-0.1363583449
H,0,0.01053806,1.8889720018,0.3366765577
H,0,0.1743902857,1.0806824167,-1.2250524543
C,0,-0.8784526717,-0.0693285954,0.2713649612
H,0,-0.7404756186,-1.0240341948,-0.2633569207
H,0,-0.7789666869,-0.3019527736,1.3442482246
C,0,-2.2754410129,0.4609758889,-0.0101764304
H,0,-2.3536068859,0.7312065175,-1.0747845157
H,0,-2.4296253276,1.3926704916,0.5537403931
C,0,-3.3559140142,-0.5444664734,0.3422025325
H,0,-3.1947431928,-1.4848715014,-0.2042544562
H,0,-3.2855734619,-0.8134286777,1.4081802784
C,0,-4.7937391692,-0.1087528975,0.1002324568
O,0,-5.6789382743,-1.0141394402,0.1739211524
O,0,-5.0375232774,1.1085423749,-0.1360514778

{26} DHLA^{-}

Charge = -1 Multiplicity = 1
C,0,3.3998023811,-1.2940619542,0.0037738874
H,0,4.1886417737,-1.8296499446,-0.5447049145
H,0,3.5426081498,-1.5312774009,1.0665308552
C,0,2.0326796285,-1.7677008887,-0.4646573753
H,0,1.9812344855,-2.8633755032,-0.3844018551
H,0,1.9029648153,-1.5309041462,-1.5310892405
C,0,0.8906625078,-1.1526748199,0.3313048283
H,0,0.983898461,-0.0553322164,0.3124541693
H,0,0.975276019,-1.4511237373,1.3891018273
C,0,-0.4698309633,-1.5672102433,-0.209796602
H,0,-0.5186064715,-2.6661161441,-0.2505018806
C,0,-1.6374966772,-1.0587672762,0.6273280946
C,0,-1.6821975198,0.4557915912,0.8139101849
H,0,-0.8285460214,0.7431661308,1.4474407483
H,0,-2.5848046947,0.7228929388,1.3815633296
H,0,-1.5798964457,-1.519230523,1.6225597456
C,0,3.664995291,0.1927098607,-0.2059861263
O,0,3.2602548106,0.7253893918,-1.2800308042
O,0,4.3102172169,0.8047042831,0.6962249427
C,0,-1.6383766164,1.2565184045,-0.4733143727
H,0,-0.7174524499,1.0731574829,-1.0381002971
H,0,-2.4804962392,1.0006330643,-1.1272339676
H,0,-0.5709452879,-1.2176253035,-1.2498295898
S,0,-1.7914219437,3.0609102642,-0.1906526085
H,0,-0.6856551015,3.1796485726,0.5776576192
S,0,-3.1853230937,-1.7063626744,-0.1605395894
H,0,-4.0421700143,-1.1634262089,0.7320289913

{27} S1-deprotonated DHLA^{2-}

Charge = -2 Multiplicity = 1
C,0,3.4355503927,-1.0388305305,0.042784872
H,0,4.2516918758,-1.4185038668,-0.587263994
H,0,3.5944734152,-1.42208894,1.0592763964
C,0,2.096496493,-1.5368277098,-0.4943726635
H,0,2.1035666553,-2.6367124732,-0.5066772845
H,0,1.9761686115,-1.2174628852,-1.5409361711
C,0,0.9085365113,-1.0491811029,0.3227277013
H,0,0.8929235273,0.0533639133,0.3152034985
H,0,1.0459337916,-1.3439797305,1.3776645647
C,0,-0.4186605007,-1.5925319656,-0.1928701895
H,0,-0.3422424808,-2.6871450443,-0.2854156197
C,0,-1.6299104826,-1.2783564463,0.6845929557
C,0,-1.7935660949,0.2217144526,0.956576238
H,0,-0.9248476519,0.5888762205,1.5297997403

H,0,-2.6738435526,0.3695063551,1.5979265558
H,0,-1.440637444,-1.7429457036,1.666567509
C,0,3.5251341798,0.4811033634,0.0570573945
O,0,3.4174904559,1.0798219431,-1.0540486524
O,0,3.6968264251,1.062175797,1.168907201
C,0,-1.9439825472,1.0688918752,-0.2911217048
H,0,-1.062693914,1.0034608214,-0.9393589807
H,0,-2.8097867728,0.7363865096,-0.876230375
H,0,-0.5958415293,-1.2200224561,-1.2156198234
S,0,-2.2591629248,2.8405702347,0.0677070728
H,0,-1.1189420049,3.0572394568,0.7610520559
S,0,-3.1763812141,-2.044977108,0.0118813328

{28} S2-deprotonated DHLA^{2-}

Charge = -2 Multiplicity = 1
C,0,3.3909614498,-1.0640271297,0.1182589176
H,0,4.2201348411,-1.5501862791,-0.4167214922
H,0,3.5001867487,-1.3356518772,1.1768041541
C,0,2.0621539043,-1.5696568609,-0.421967204
H,0,2.0533713407,-2.6690887397,-0.3889079182
H,0,1.9624932278,-1.2933209395,-1.4821842417
C,0,0.8661404251,-1.0373623691,0.3539312474
H,0,0.9023649317,0.0634965556,0.3734802003
H,0,0.9336953807,-1.3666017488,1.4040305688
C,0,-0.4542534853,-1.5028170967,-0.2429420885
H,0,-0.4343092882,-2.6001307331,-0.3352618977
C,0,-1.667649006,-1.0999909334,0.5854722542
C,0,-1.8222370141,0.4030895962,0.8116172019
H,0,-1.0119342934,0.7254147041,1.4857945833
H,0,-2.7567046109,0.5839520286,1.3650740655
H,0,-1.5945304961,-1.5912360116,1.5658085036
C,0,3.6090187005,0.4379073795,-0.0277688483
O,0,3.2390992789,0.9926555542,-1.1031438624
O,0,4.1848397451,1.0411103598,0.9259802884
C,0,-1.802541375,1.2764330753,-0.4314697955
H,0,-0.8376379076,1.1617155857,-0.946503774
H,0,-2.5734382207,0.9252844524,-1.1325496104
H,0,-0.5514364518,-1.1092459575,-1.2673098845
S,0,-2.0755712229,3.0627126998,-0.0607062832
S,0,-3.1535025636,-1.8404511832,-0.2465184892
H,0,-4.0660440389,-1.3481001317,0.619544405

{29} Fe^{2+} – LA^{-} (CO)

Charge = 1 Multiplicity = 5

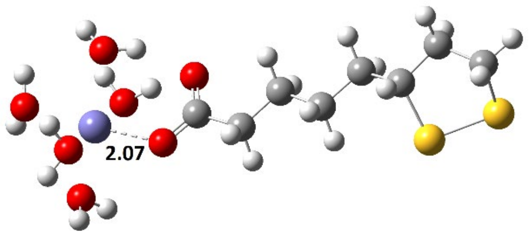

C,0,-7.3303529821,-0.9163388576,0.3974927806
H,0,-7.4885833616,-0.8698484749,1.4816575305
H,0,-8.154474688,-1.4730105272,-0.060694093
C,0,-5.9748555387,-1.4889138547,0.047076734
H,0,-5.8943513514,-1.5999645304,-1.0434473622
H,0,-5.851283053,-2.4846081961,0.4940058767
C,0,-4.8530627806,-0.5890012019,0.5468906368
H,0,-4.7090970275,-0.7404536902,1.6256069776
S,0,-7.3684133837,0.7713952003,-0.3031919219
S,0,-5.4135428905,1.195502134,0.4025010899
C,0,-3.5426083939,-0.815851345,-0.1816285476
H,0,-3.3124118402,-1.8921108203,-0.1109387402
H,0,-3.6813379492,-0.5932707598,-1.2516221868
C,0,-2.3761041716,-0.0193457869,0.3772898321
H,0,-2.5638951493,1.059518834,0.2468384484
H,0,-2.308429551,-0.190485986,1.4641259238
C,0,-1.0496052064,-0.3811965358,-0.2719714596
H,0,-1.1124587438,-0.2210600226,-1.3584723222
H,0,-0.8574575578,-1.4548084841,-0.1284967888
C,0,0.0964274074,0.4250255996,0.3082739221

```
H,0,-0.0502594816,1.4996208691,0.1229060153
H,0,0.1189507595,0.3236110617,1.4034029377
C,0,1.4777790243,0.0744925794,-0.1855737438
O,0,2.4425311836,0.6920186716,0.3854292731
O,0,1.6404570228,-0.7822955412,-1.0929995521
Fe,0,4.4287662379,0.1331883479,0.1787579283
O,0,6.4832229254,-0.5246338756,-0.0870536338
O,0,3.8568206119,-2.086891414,0.1321643497
H,0,4.4567038097,-2.5490108894,-0.4692577382
H,0,3.0523805577,-1.9096021086,-0.3979490706
H,0,6.9649398517,0.1249342063,-0.6199129791
H,0,6.5042816256,-1.3412512536,-0.6071517303
O,0,4.11589129,0.1278587066,-2.1263496288
O,0,4.6841486352,0.1409238014,2.401647926
H,0,4.6657583554,-0.5881290771,-2.4729730401
H,0,3.2243258384,-0.2670391009,-2.0149597777
H,0,5.0539356061,-0.701391555,2.7007558893
H,0,5.3469110057,0.8048139517,2.6386960595
O,0,5.1542412517,2.2187659278,0.1409666491
H,0,4.5982185027,2.7507956137,-0.4452339068
H,0,5.0555265993,2.6204783838,1.0157304432
```

{30} Fe^{2+} – LA^{-} (COO)

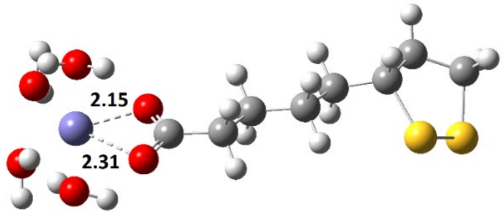


```
Charge = 1 Multiplicity = 5
C,0,-6.7951496457,1.0658237367,0.0247551268
H,0,-7.2948246715,0.8038533129,-0.9152399309
H,0,-7.4214129591,1.7738599559,0.5778183808
C,0,-5.3903123616,1.5895257728,-0.1927437144
H,0,-5.0138580008,1.9923721311,0.7576515494
H,0,-5.3992608086,2.4133393225,-0.9181904844
C,0,-4.4249875378,0.5018661815,-0.6795116764
H,0,-4.3924355394,0.4843068283,-1.7774475646
S,0,-6.6192496965,-0.4372690515,1.0494692884
S,0,-5.1334317386,-1.1767696674,-0.2462761313
C,0,-3.0258725676,0.6932873306,-0.1253642034
H,0,-2.7299963856,1.7350030655,-0.3358242163
H,0,-3.0622552547,0.5974866432,0.9723455591
C,0,-1.9810726731,-0.2462875168,-0.7008411641
H,0,-2.2415532205,-1.2885713213,-0.4545227832
H,0,-1.9924909455,-0.1766066298,-1.8008863565
C,0,-0.5835628006,0.065209503,-0.1897777362
H,0,-0.579835977,0.0401224456,0.9106548427
H,0,-0.3167935603,1.0928367492,-0.4748623391
C,0,0.4567772945,-0.9017363428,-0.725877632
H,0,0.2346082232,-1.9325345229,-0.4186053874
H,0,0.429544536,-0.9117980062,-1.8271849255
C,0,1.8815750288,-0.6017419347,-0.3478346974
O,0,2.7707455441,-1.4915725152,-0.5074801446
O,0,2.2004197426,0.5499055446,0.0934855097
Fe,0,4.3022567957,0.0996491413,0.1703130647
O,0,6.0724374063,-1.0905777119,-0.0829899009
O,0,5.0432256196,2.0087416938,0.8298988652
H,0,4.6268721097,2.3066245043,1.6512725604
H,0,4.8583239569,2.7012348351,0.1796353994
H,0,6.0639581741,-1.5326940939,-0.9447000141
H,0,6.0948086046,-1.8055759867,0.5699466225
O,0,4.1542038596,-0.5719257125,2.2771113754
O,0,4.4735171639,0.7958108713,-1.9546981983
H,0,4.3943256779,0.1370131479,2.8901191109
H,0,3.2278099483,-0.7719850841,2.4730590446
H,0,5.3583299995,0.6558290191,-2.3197691451
H,0,3.8854366592,0.2599243614,-2.5055789541
```

{31} Fe^{2+} – $DHLA^-$ (CO)

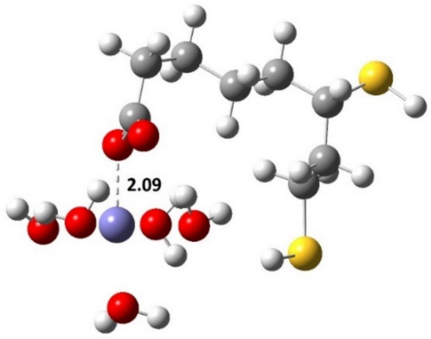


```
Charge = 1 Multiplicity = 5
C,0,-0.6649923437,-3.2316178147,0.5136366162
H,0,-1.1973517877,-3.7115637993,1.345066862
H,0,-0.5359511173,-3.9752269327,-0.2814044377
C,0,0.6957399835,-2.7265813418,0.990434842
H,0,1.3000533763,-3.5896978154,1.3024925718
H,0,0.5681144279,-2.0952049066,1.8821810776
C,0,1.4382882905,-1.941723347,-0.0815040339
H,0,0.8769314395,-1.0191769827,-0.3082646429
H,0,1.4633812516,-2.5233842814,-1.0178783871
C,0,2.8592800378,-1.5971237196,0.3377928873
H,0,3.3956191096,-2.5323725849,0.5577138887
C,0,3.633095993,-0.8295035193,-0.7285135904
C,0,3.0396380654,0.5252449562,-1.1126413262
H,0,2.0787343452,0.3463146426,-1.6217257582
H,0,3.6906280716,1.0031768048,-1.8582484275
H,0,3.6782208206,-1.4476394444,-1.6350805339
C,0,-1.5217650428,-2.1036885089,-0.0073148265
O,0,-1.7993537532,-1.1694639789,0.8192058633
O,0,-1.8994626951,-2.1082215258,-1.2102123366
C,0,2.8233302878,1.4751395561,0.0502818867
H,0,2.1207742333,1.0678212941,0.786854426
H,0,3.7643424825,1.671333651,0.5775657413
H,0,2.8414994234,-1.0265006411,1.2800731107
S,0,2.2238664129,3.1279632205,-0.4708696524
H,0,1.0521021342,2.6944591267,-0.9953183597
S,0,5.3761813768,-0.6702152802,-0.1182105573
H,0,5.8455401585,-0.0024619574,-1.1951016655
Fe,0,-2.5661854968,0.6679043709,0.1943235004
O,0,-4.1012517096,-0.3806966595,-1.0783398744
H,0,-3.5025522626,-1.1350451543,-1.2757310441
O,0,-3.8928367316,0.8231923888,1.9439265393
H,0,-3.3676826745,0.811522486,2.7565707202
O,0,-0.9424728815,1.6640823014,1.4490026164
H,0,-0.7419134117,2.5267461998,1.0583310416
O,0,-3.1768631726,2.6366663061,-0.5060141882
H,0,-2.4185948261,3.0822132716,-0.9119754101
O,0,-1.2113371098,0.6334921897,-1.6668573926
H,0,-1.3435732942,-0.322126938,-1.828231098
H,0,-3.4225919039,3.1897925818,0.2499286692
H,0,-4.7984651732,-0.7410786334,-0.5138909744
H,0,-0.2830195807,0.7122364501,-1.4031545886
H,0,-4.4709438999,0.0497123198,2.0046412741
H,0,-0.1412118535,1.1357316498,1.3176279713
```

{32} Fe^{2+} – $DHLA^-$ (COO)

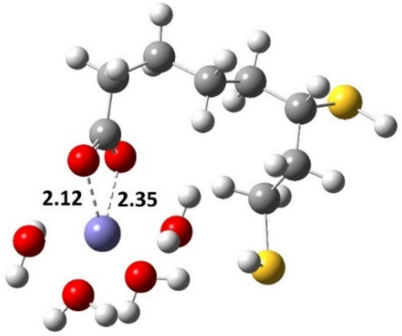


```
Charge = 1 Multiplicity = 5
C,0,-0.3420184576,3.2584210967,0.1813687352
H,0,-0.8183359367,4.0892796488,-0.3580704331
H,0,-0.4116900531,3.5035902595,1.2492183671
C,0,1.1045477189,3.0803801547,-0.2539594474
H,0,1.6440989079,4.0289577728,-0.1363184908
H,0,1.1247597107,2.8463489057,-1.3276849612
C,0,1.8138233476,1.9692654165,0.5199297887
H,0,1.0871670027,1.1818467606,0.7713629933
H,0,2.189222585,2.3520665257,1.4809868997
C,0,2.9533247423,1.3509980782,-0.2760206791
H,0,3.7035892922,2.1241717257,-0.5001943634
C,0,3.6327093689,0.1864416336,0.4350104173
C,0,2.706778147,-0.9757516631,0.7974590179
H,0,2.0507607761,-0.6528310762,1.6215363801
H,0,3.3122841973,-1.7990421777,1.2019009044
H,0,4.0925617138,0.554694291,1.3616355043
```

```
C,0,-1.1746657051,2.0274584351,-0.069586948
O,0,-0.870467098,1.2146026837,-0.982649596
C,0,1.851544106,-1.4906950536,-0.3466800822
H,0,1.1042729598,-0.7525201146,-0.6655315442
H,0,2.4644285435,-1.7421351346,-1.2205634991
H,0,2.5639245872,1.0114981664,-1.2498898787
S,0,0.9760797116,-3.0490108774,0.0806326969
H,0,0.3420068956,-2.5491639112,1.1679051126
S,0,5.0230860223,-0.3638611797,-0.6603872802
H,0,5.4853569906,-1.331809991,0.1613628953
Fe,0,-2.4838887666,-0.1826174562,-0.0019835866
O,0,-2.1913670524,1.8009673242,0.6771340591
O,0,-4.0840524804,-1.1420655337,1.0837649939
H,0,-4.8029553673,-1.4755485436,0.5288745913
O,0,-1.1228645876,-0.8831157887,1.6686803596
H,0,-1.6338586815,-0.9081135078,2.4902100247
O,0,-3.9210273383,0.3929641391,-1.5883264323
H,0,-4.6121179881,-0.2750279224,-1.6971646358
O,0,-1.8081515601,-1.8051647459,-1.2518655715
H,0,-2.4996298017,-2.4664626495,-1.3984566433
H,0,-3.4657861224,0.4284810141,-2.4414483267
H,0,-3.8093198676,-1.8878799696,1.6351998903
H,0,-0.428204341,-0.2268956686,1.8286589578
H,0,-1.0410321214,-2.2985070669,-0.8942761898
```

{33} Fe^{2+} – $DHLA^{-}$ (CO) (5-coord)

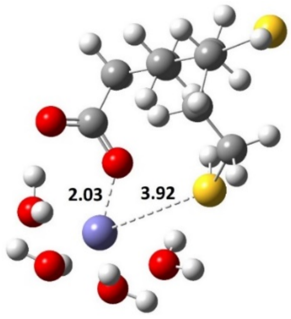


```
Charge = 1 Multiplicity = 5
C,0,-0.1618254436,3.0632544798,-0.5468871141
H,0,-0.6846636872,3.5204779737,-1.3966912744
H,0,0.0777924756,3.8568022039,0.1705266273
C,0,1.1151205364,2.3797581966,-1.0293455905
H,0,1.7702744739,3.1334562337,-1.4878718382
H,0,0.8701839556,1.6561562833,-1.8218337155
C,0,1.8624453868,1.6671634281,0.087856488
H,0,1.2041742011,0.9115910536,0.5463866442
H,0,2.1074102225,2.3818378224,0.8895380072
C,0,3.1380101885,1.0076317786,-0.4159380004
H,0,3.8161186326,1.7934127159,-0.7816910908
C,0,3.854337017,0.1841615757,0.6460713551
C,0,3.09737243,-1.0438325348,1.1513590738
H,0,2.224968415,-0.7046180559,1.7279750197
H,0,3.7432914225,-1.5581422686,1.876692252
H,0,4.0583174661,0.8313017301,1.509645473
C,0,-1.1011098234,2.082965317,0.1144836657
O,0,-1.5239043745,1.1199409,-0.6210289004
O,0,-1.4055309177,2.2363320106,1.3214478085
C,0,2.6637040015,-2.0519540867,0.0911815744
H,0,3.3282784048,-2.0293554549,-0.7805761774
H,0,2.714112327,-3.0687212449,0.4933941959
H,0,2.9146697907,0.3758634761,-1.2921722673
S,0,0.9222063314,-1.9412629268,-0.4957689324
H,0,0.9930044829,-0.7323282151,-1.0997204523
S,0,5.5039489744,-0.2766275974,-0.0637966309
H,0,5.9143048965,-1.0232933822,0.9851236498
Fe,0,-2.6716269006,-0.446714243,-0.0297156049
O,0,-2.665222371,-2.5024606316,0.5786675753
H,0,-2.4018190791,-3.0938490637,-0.1414103839
O,0,-2.681854683,0.0160124523,2.1713348297
H,0,-2.1942663066,0.8672180736,2.0395802536
O,0,-4.7363592155,0.1418436109,-0.0903048248
H,0,-5.0167985405,0.517056822,-0.9366292662
O,0,-2.8247629156,-1.1784030444,-2.1219941043
H,0,-2.180449902,-1.8739632649,-2.3147190889
H,0,-4.9444126035,0.8105835502,0.5772095897
H,0,-2.0206134494,-2.6502910933,1.2856972973
```

H,0,-2.6039921426,-0.4585099723,-2.7298273635
H,0,-2.0636906772,-0.5771866079,2.618926241

{34} Fe^{2+} – $DHLA^{-}$ (CO) (5-coord)

Charge = 1 Multiplicity = 5
C,0,-0.2564511328,2.8764250527,0.8595070078
H,0,-0.8519447882,3.710550581,1.2580131279
H,0,0.6503283923,2.8341117845,1.4738103316
C,0,0.0754346925,3.1518117372,-0.6069147381
H,0,0.7594545048,4.011839073,-0.6378991165
H,0,-0.8334867855,3.4636166312,-1.1381275127
C,0,0.6954085133,1.9750226234,-1.3532324488
H,0,0.9531835699,2.2889747363,-2.374946358
H,0,-0.0617486108,1.1865242442,-1.4669508803
C,0,1.9334748431,1.3851434466,-0.6925234081
H,0,1.7635413979,1.2321029574,0.3846027269
C,0,2.3759241947,0.0685197453,-1.3179947379
C,0,3.7991099781,-0.3391922038,-0.9535559475
H,0,4.4739178722,0.3898748269,-1.4296314289
H,0,4.0299010307,-1.3163440571,-1.4007605038
H,0,2.3087791349,0.1465492765,-2.4113654607
C,0,-1.0542506674,1.619575245,1.1240388237
O,0,-0.8352273524,0.9566093261,2.1672442191
O,0,-1.9515797817,1.3062005314,0.2621726667
C,0,4.0872226253,-0.3788968975,0.5355632552
H,0,3.9411375093,0.6007747335,1.0021175686
H,0,3.4287218919,-1.0889041878,1.0498299358
H,0,2.7715387195,2.0961383586,-0.7678094163
S,0,5.7867759787,-0.9519809532,0.9117316975
H,0,6.4221247442,0.0395668034,0.2486510769
S,0,1.1436722669,-1.2378771768,-0.8188346998
H,0,1.6912820571,-2.2404860405,-1.5406749655
Fe,0,-2.6415442128,-0.5973288226,0.0353827997
O,0,-4.7121170709,-0.4679189743,-0.5210501543
H,0,-5.2519253469,-0.015465596,0.1421714966
O,0,-1.9886072599,-0.6690811637,-2.1006989907
H,0,-1.0298363294,-0.8331963705,-2.0183237096
O,0,-2.4548547537,-2.7218701475,0.0506477799
H,0,-2.0631472767,-3.0650430193,0.8667293421
O,0,-3.071378885,-0.6982253239,2.2634227319
H,0,-2.2657161693,-0.1701148486,2.4743097353
H,0,-1.9094119186,-3.0679842515,-0.6704063766
H,0,-4.8457620486,0.0218231457,-1.3447009148
H,0,-2.889817407,-1.5895560731,2.591122419
H,0,-2.0607851192,0.2068882476,-2.5055019733

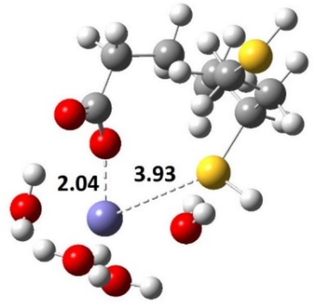

{35} Fe^{2+} – $DHLA^{2-}$ (CO, S1 *cis*)

Charge = 0 Multiplicity = 5
C,0,-1.4997762954,2.846657144,0.6555895912
H,0,-2.5203333929,3.1183452689,0.3502334606
H,0,-1.1615103118,3.6767014605,1.2953434809
C,0,-0.5939139007,2.7032053614,-0.554165571
H,0,-0.7335024947,3.5786425157,-1.2063440244
H,0,-0.8925953368,1.8310935317,-1.1482343042
C,0,0.8847882167,2.5799246982,-0.1989746718
H,0,0.9923421987,1.8972076315,0.6507511638
H,0,1.2365770207,3.5592500161,0.1611449166
C,0,1.7745470134,2.1390716779,-1.364878234
H,0,2.7913998547,2.536490592,-1.207882479
C,0,1.956464106,0.6443447207,-1.6682018931
C,0,2.9217173568,-0.0874832412,-0.7268171796
H,0,3.9286202424,0.3368130487,-0.8825189911
H,0,2.9768333492,-1.1376518264,-1.0473852671
H,0,2.4628787591,0.6150108722,-2.6434370073

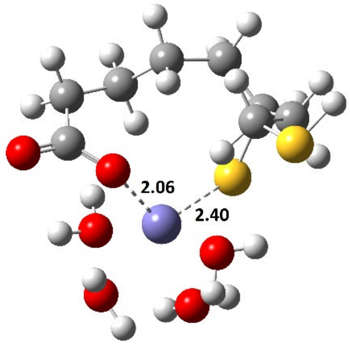

C,0,-1.6429128124,1.639877056,1.5697391353
O,0,-1.0040706756,0.562239732,1.31334951
O,0,-2.3902321872,1.7622368701,2.5701270463
C,0,2.6271319229,-0.0377266253,0.7616621736
H,0,2.7922121956,0.962961562,1.1732876257
H,0,1.5940291871,-0.3253319625,0.986600587
H,0,1.4037630079,2.6280852899,-2.2798478428
S,0,3.6532997643,-1.2173670978,1.7269540736
H,0,4.8485049792,-0.7428297449,1.3103049781
S,0,0.3950423845,-0.3078757042,-2.0302075686
Fe,0,-1.0629053074,-0.8303611156,-0.2022238263
O,0,-2.6728144532,-1.9021656335,1.2739284472
O,0,-1.7658039535,-2.6366471928,-1.5250986984
O,0,-2.9777078117,0.034400911,-1.0287262219
O,0,0.2848289711,-2.3358908602,0.7874840168
H,0,-2.8426863114,0.8816142176,-1.4767016867
H,0,-3.5404779038,0.2409124815,-0.2677310408
H,0,-2.3203166213,-1.7041562388,2.1527796919
H,0,-2.5642833004,-2.8577104353,1.173299791
H,0,0.9998035852,-2.5463559623,0.1679466149
H,0,-0.2051263536,-3.1637521165,0.8965132271
H,0,-1.1425061495,-2.7230294166,-2.261531885
H,0,-2.5813215423,-2.307350486,-1.9297351384

{36} Fe^{2+} – $DHLA^{2-}$ (CO)

Charge = 0 Multiplicity = 5

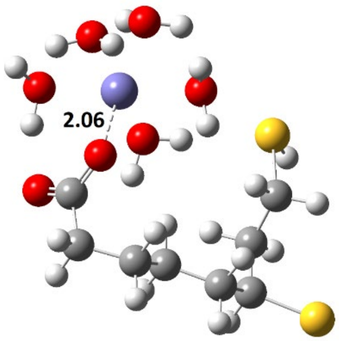

C,0,-0.3258154215,-3.4158111241,-0.3178473088
H,0,-0.8730999603,-4.1862203806,0.2434287029
H,0,0.0001791062,-3.8735931205,-1.2597613964
C,0,0.8781917802,-2.9344820039,0.4846820635
H,0,1.5562173613,-3.7851469618,0.6438281604
H,0,0.5561042686,-2.6022072619,1.483092856
C,0,1.6254208827,-1.8042896843,-0.2074910264
H,0,0.9477087811,-0.9392003999,-0.2943204042
H,0,1.8728187322,-2.1059745291,-1.2401783467
C,0,2.8956056704,-1.3884760959,0.5227457334
H,0,3.53581845,-2.2733906031,0.6602556487
C,0,3.7184241554,-0.3142798922,-0.1892518545
C,0,2.8927627578,0.9336753703,-0.5255429259
H,0,2.0885432039,0.6676984893,-1.2323690388
H,0,3.5399371574,1.6524945646,-1.049737933
H,0,4.0256167737,-0.7397414226,-1.158807387
C,0,-1.3115347905,-2.3158576209,-0.6412353647
O,0,-1.6476971155,-1.5425684408,0.3212998954
O,0,-1.7602058712,-2.2137180824,-1.8110206534
C,0,2.2832763784,1.6129084048,0.6842888722
H,0,1.6207673347,0.9353038011,1.2372494277
H,0,3.062621168,1.9519327241,1.3743700068
H,0,2.6402539786,-1.0466092078,1.5399176118
S,0,1.208585364,3.0390864821,0.2459532615
H,0,2.0477676265,3.5838158325,-0.6639142215
S,0,5.2658223253,0.0922134536,0.7419908884
Fe,0,-2.4135242783,0.3594184058,0.0902855995
O,0,-3.1740137207,2.3955252053,0.0138154361
O,0,-1.2416763638,1.0476613972,1.9002211128
O,0,-3.6188785301,-0.2138941361,-1.7164652501
O,0,-4.1545498126,-0.1708149969,1.4497713016
O,0,-0.8271067694,1.022035329,-1.3975046941
H,0,-3.7974471613,-0.4535074655,2.3037888445
H,0,-4.5792394221,-0.9604235801,1.0857947709
H,0,-3.0519721205,-1.0028845716,-1.9039425833
H,0,-4.473276744,-0.5671355875,-1.435168113
H,0,-3.3558583256,2.6354737719,-0.9066521887
H,0,-2.4618924269,2.9918231496,0.2900815795
H,0,-0.3038582182,0.273792681,-1.719324616

```
H,0,-0.1792704336,1.6417412297,-1.0032050401
H,0,-0.5103895291,0.4240834644,2.0200223346
H,0,-1.8019812411,0.9191674131,2.6787702388
```

{37} Fe^{2+} – $DHLA^{2-}$ (COO)

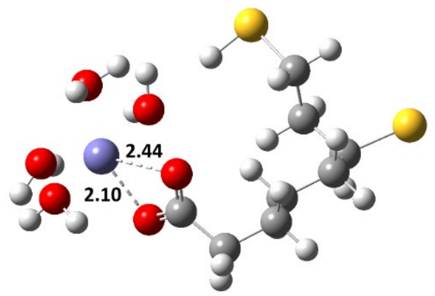


```
Charge = 0 Multiplicity = 5
C,0,-0.6145967425,-3.0529179891,-0.0975069248
H,0,-1.1162484892,-3.8524494336,0.4630555574
H,0,-0.5899947997,-3.3449072368,-1.1541740761
C,0,0.8040731755,-2.8526834974,0.4303509686
H,0,1.3542670107,-3.7986098134,0.3288358085
H,0,0.7673295504,-2.6274599981,1.5068116575
C,0,1.5525592951,-1.7463176325,-0.2983517226
H,0,0.9941462549,-0.7998050962,-0.1960484894
H,0,1.5735563506,-1.9717913278,-1.3785223939
C,0,2.9745280277,-1.5684665503,0.2183977018
H,0,3.47176833,-2.5508498102,0.2336581119
C,0,3.8420066358,-0.6148845489,-0.6008265918
C,0,3.2087298692,0.7710694253,-0.7730335178
H,0,2.2721925864,0.6833226683,-1.3519559194
H,0,3.8882851845,1.3913090757,-1.3741857354
H,0,3.911938352,-1.0406419942,-1.6154938637
C,0,-1.4376514857,-1.8017835642,0.0516210037
O,0,-1.6495626909,-1.3162252144,1.1946764305
O,0,-1.9044411979,-1.2342369899,-0.9980715382
C,0,2.9079246677,1.4876716579,0.5279297747
H,0,2.1379612773,0.9711894953,1.1139047936
H,0,3.8121551115,1.548879371,1.1460128267
H,0,2.945169269,-1.2336795532,1.2685874876
S,0,2.3735134422,3.2273362463,0.2866125153
H,0,1.2918837606,2.9414033785,-0.4741801202
S,0,5.5668118354,-0.5210020911,0.0673129976
Fe,0,-2.8895929296,0.3669772361,-0.058904248
O,0,-3.285929835,1.5310341533,1.7054559237
O,0,-3.849746928,1.4313127879,-1.6635613815
O,0,-4.7374553747,-0.8192907923,0.2630173209
O,0,-1.0191956857,1.6257706356,-0.280599723
H,0,-4.4833168191,-1.7402502787,0.4175195493
H,0,-5.2683442792,-0.8357031833,-0.5456884528
H,0,-4.5202462725,0.9010483175,-2.1167305911
H,0,-3.2395569273,1.7254303998,-2.354554612
H,0,-2.463670453,1.6270279416,2.2084701166
H,0,-3.8895688513,1.0527752542,2.2925765013
H,0,-0.9953712468,2.2978389854,0.4157928177
H,0,-1.0427439786,2.1300025661,-1.1063559633
```

{38} Fe^{2+} – $DHLA^{2-}$ (S1)

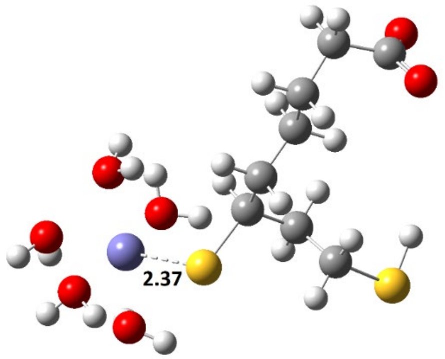


```
Charge = 0 Multiplicity = 5
C,0,4.6297536467,-2.3625025137,-0.0962049905
H,0,5.2587185401,-3.0326118325,-0.6982963133
H,0,4.2942514471,-2.9243414173,0.7852251075
C,0,3.4228909902,-1.9164714107,-0.915983534
H,0,2.8519769166,-2.8050368505,-1.2232924707
H,0,3.7623711743,-1.4307116595,-1.8432710189
C,0,2.5089051229,-0.9677694245,-0.1540130915
H,0,3.0703833148,-0.0561038993,0.1076846228
H,0,2.2170142728,-1.4318154861,0.8040011873
C,0,1.2588917006,-0.6011972391,-0.9431988056
H,0,0.7602306511,-1.5269776844,-1.2717036583
C,0,0.2460039814,0.2283474002,-0.1594754373
C,0,0.8186042871,1.5208638235,0.4243113538
H,0,1.5958578883,1.2681169677,1.1639636067
H,0,0.0227534131,2.0427566129,0.975489318
H,0,-0.0904546467,-0.3819458863,0.6933178427
```

C,0,5.492260384,-1.1914455823,0.3533375029
O,0,5.9834397871,-0.4500319588,-0.5480459132
O,0,5.6687214276,-1.0190416083,1.5955456762
C,0,1.4102823274,2.4586017914,-0.610305687
H,0,2.2373451617,1.9923473701,-1.1574210484
H,0,0.6527634463,2.7523146395,-1.3469014681
H,0,1.5447195818,-0.0714152748,-1.8665959608
S,0,2.0128988913,4.0348542414,0.1079941234
H,0,2.919347437,3.4747482854,0.9396085918
S,0,-1.2576234673,0.5693840363,-1.1989619866
Fe,0,-3.0261128944,-0.4032841514,0.0486385042
O,0,-4.7803804603,-1.4336232279,1.1390783487
O,0,-4.2001042246,1.4501071346,0.2974104583
O,0,-2.1415129563,-2.4408676262,0.0397415176
O,0,-2.327025013,-0.0114106281,2.2038044864
O,0,-4.1563883108,-0.8799303548,-1.8380853517
H,0,-1.5840062101,0.6096358828,2.214404666
H,0,-1.9603849772,-0.8392517305,2.5461631592
H,0,-1.4050782646,-2.4418489293,-0.5905552828
H,0,-1.7310807703,-2.5982525092,0.902963341
H,0,-5.4815549715,-1.6077703968,0.4956969363
H,0,-5.1579879329,-0.7742856987,1.7384271435
H,0,-3.8903177671,-0.3165019739,-2.5780744371
H,0,-5.0967382377,-0.6948993854,-1.7035570748
H,0,-3.6328290665,2.2071326389,0.0892353038
H,0,-4.44068362,1.5620625156,1.2290057325

{39} Fe^{2+} – $DHLA^{2-}$ (CO, S2 *cis*)

Charge = 0 Multiplicity = 5

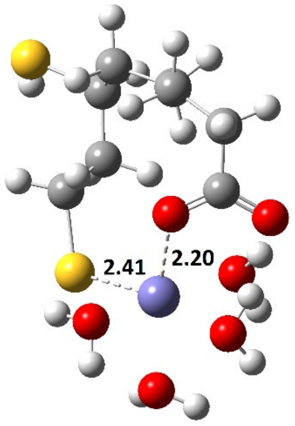

C,0,-0.2475332013,3.3387038404,0.4777789349
H,0,0.1507289061,3.960298128,1.2926222493
H,0,-0.3695526236,4.0033746888,-0.3872278319
C,0,-1.5750725624,2.7123184913,0.8793925249
H,0,-2.2795458107,3.5002017313,1.1787685665
H,0,-1.4202915876,2.0815879149,1.76627239
C,0,-2.1645871633,1.8636051367,-0.2426973483
H,0,-1.3456842491,1.2949513397,-0.7048853378
H,0,-2.5592335302,2.5124580135,-1.0384323773
C,0,-3.2446944206,0.8911310644,0.2272511556
H,0,-4.2199043184,1.39681639,0.2651556606
C,0,-3.3536011644,-0.3513199086,-0.6547601815
C,0,-2.0578681076,-1.1592383411,-0.7771998849
H,0,-1.3644951349,-0.5980725823,-1.422013473
H,0,-2.2667963078,-2.0939577677,-1.3183623572
H,0,-3.6686010442,-0.0480553139,-1.6625776757
C,0,0.8241162544,2.3195398135,0.1386308155
O,0,0.8193374122,1.215125528,0.7608567587
O,0,1.6796473532,2.6231055481,-0.7490251049
C,0,-1.3566010766,-1.4762995101,0.5330910996
H,0,-1.1875585852,-0.5622015946,1.114387302
H,0,-1.9818942658,-2.1370945402,1.147640126
H,0,-3.0270725118,0.5755080306,1.2593447393
S,0,0.2745398555,-2.3020377271,0.2896233197
S,0,-4.7413793722,-1.3670876105,0.039608279
H,0,-4.6998063126,-2.3387698191,-0.8987652884
Fe,0,1.9117212264,-0.5498153556,0.0367436065
O,0,3.4369724077,-2.0912450006,-0.5670005172
O,0,3.7276329241,0.9040426427,0.0820314412
O,0,1.6425509098,0.1389173761,-2.0373419157
O,0,2.436016016,-0.913093204,2.2190280697
H,0,3.0661988311,-2.8618443902,-1.0195987777
H,0,4.0619359309,-1.7050754207,-1.196975169
H,0,4.3076168691,0.6695723863,-0.6547860031
H,0,3.1695734251,1.6423812933,-0.2513746892
H,0,1.711663393,1.1002963282,-1.8449251855

H,0,2.4299672762,-0.080754224,-2.555066815
H,0,1.80237359,-1.6480409264,2.3067991912
H,0,3.3093607697,-1.329213449,2.2605187033

{40} Fe^{2+} – $DHLA^{2-}$ (CO, S2 *cis*) (5-coord)

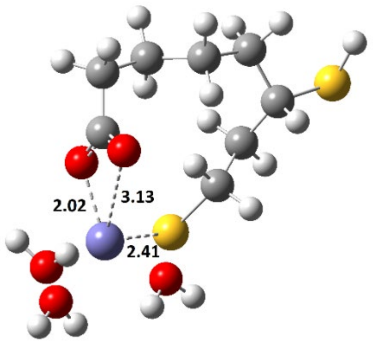

Charge = 0 Multiplicity = 5
C,0,0.1772209296,3.0708262606,-0.5197734503
H,0,-0.1777956826,3.5185601293,-1.455444979
H,0,0.348567114,3.8740918224,0.2078261106
C,0,1.4809643891,2.3068132879,-0.7767398741
H,0,2.1313251975,2.9162297789,-1.4192599807
H,0,1.2566238377,1.3953765127,-1.3514946037
C,0,2.2444057841,1.9676413252,0.4998447498
H,0,1.5758519668,1.5029277135,1.2401446913
H,0,2.5702075973,2.9136707852,0.9556288454
C,0,3.4691176459,1.0740951289,0.2683222919
H,0,4.2799260683,1.4018150102,0.9333702505
C,0,3.2606488987,-0.4213767825,0.5059209435
C,0,2.2212667307,-1.1103510157,-0.3888344713
H,0,2.5147026798,-2.1543505076,-0.5777896923
H,0,2.2012293755,-0.6181583403,-1.3731916034
H,0,2.9868204074,-0.582575646,1.5590574871
C,0,-0.8882077496,2.1320473502,-0.0039023403
O,0,-1.4329776742,1.3787349281,-0.895551832
O,0,-1.1651785978,2.0796598712,1.2135575494
C,0,0.8281815471,-1.1354435487,0.2235202057
H,0,0.8597902463,-1.7110066104,1.1594943878
H,0,0.5019372921,-0.1227697542,0.4882830347
H,0,3.8405397891,1.216544133,-0.7595242875
S,0,-0.4278551676,-1.8785475375,-0.9095195638
S,0,4.8603269735,-1.3174325037,0.229395112
Fe,0,-2.2475261615,-0.3964129692,-0.3624525396
O,0,-3.7142541929,-1.4383883516,-1.5881195354
O,0,-2.2490949015,-1.1416743731,1.6817719475
O,0,-4.2253600148,0.6978435884,0.3816504263
H,0,-4.2686505316,1.4579601553,-0.2156207316
H,0,-3.9350767639,1.0727999337,1.2250810095
H,0,-3.1212134624,-1.1855565473,2.0995627632
H,0,-1.8976148673,-2.0442763004,1.6997296641
H,0,-3.549155676,-2.3837131237,-1.7100211163
H,0,-4.5978017254,-1.3764625177,-1.1981982561
H,0,5.6442366988,-0.4781892851,0.9424593872

{41} Fe^{2+} – $DHLA^{2-}$ (S2)

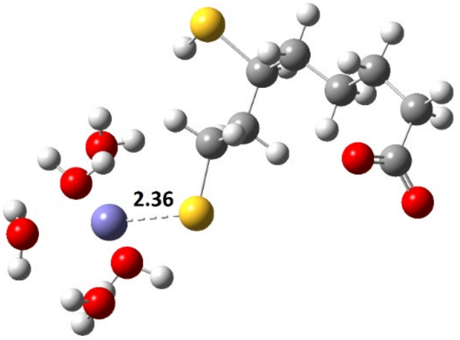

Charge = 0 Multiplicity = 5
C,0,5.6709475713,-0.8586002875,-0.1178786834
H,0,6.6207663453,-1.0028519387,0.4173842348
H,0,5.9224618179,-0.7439166007,-1.1807029905
C,0,4.966598451,0.3884760115,0.3962638399
H,0,5.6532596492,1.2458617326,0.3467475884
H,0,4.7131972123,0.2552820103,1.45853003
C,0,3.7021738447,0.7025570339,-0.3902547968
H,0,3.0807486561,-0.2051784543,-0.4464673817
H,0,3.9672441918,0.9552300572,-1.430004831
C,0,2.8988390467,1.8380560682,0.227573883
H,0,3.5489549525,2.7190953556,0.3423222333
C,0,1.6778525344,2.2303357832,-0.5971236092
C,0,0.7259864232,1.0796428949,-0.9120623838
H,0,1.253222844,0.3887011232,-1.5878915279
H,0,-0.1234375486,1.4654860928,-1.4971948438
H,0,2.0245946825,2.6550321661,-1.5491659351
C,0,4.8703353845,-2.1440149438,0.0627333114
O,0,4.2654530648,-2.3169999316,1.1609550986
O,0,4.8815009454,-2.9837799765,-0.8853377733

C,0,0.2124657116,0.296739945,0.2835195043
H,0,1.0533552538,-0.0791933805,0.8811448255
H,0,-0.3838034456,0.9428147602,0.9422284595
H,0,2.5886831523,1.5500857958,1.2448693098
S,0,-0.7892474688,-1.1645947273,-0.2257262042
S,0,0.8316499345,3.6109127855,0.3088177321
H,0,-0.2011321703,3.7368769626,-0.5541820654
Fe,0,-3.0591872491,-0.5547884632,0.0066340892
O,0,-5.3033033332,-0.0834037368,0.3327641682
O,0,-2.8585624022,1.7083816262,-0.1503115698
O,0,-3.7961958825,-2.7179157228,0.0801947047
O,0,-3.5616119935,-0.5804157542,-2.1426165623
O,0,-2.9553787264,-0.426462682,2.2091573802
H,0,-5.4729157302,0.8161628421,0.0203511278
H,0,-5.8062137937,-0.6527300402,-0.2661338667
H,0,-4.1823671437,-2.849080366,0.9581381809
H,0,-4.5536962,-2.7446185677,-0.5220766519
H,0,-2.8920670898,-1.1017480919,-2.6094583536
H,0,-4.3920559571,-1.0637570064,-2.2652917399
H,0,-2.4530959896,2.0156687041,0.6738498395
H,0,-2.2125578923,1.9332530043,-0.8356185938
H,0,-2.0313749779,-0.3265940193,2.4815723299
H,0,-3.4094506751,0.3652349361,2.532452493

{42} Fe^{2+} – $DHLA^{2-}$ (CO)

Charge = 0 Multiplicity = 5
C,0,0.1360003918,3.3264298229,0.0058121597
H,0,0.702179744,3.9595208457,0.7009799493
H,0,-0.1429976928,3.9377246059,-0.8605800064
C,0,-1.1150353259,2.7817619427,0.6944206955
H,0,-1.7722188071,3.6234724179,0.9538115152
H,0,-0.835405399,2.297737749,1.6419662806
C,0,-1.8688992693,1.7854305976,-0.1734976008
H,0,-1.2097977578,0.9263090533,-0.3880796
H,0,-2.1024347798,2.2430922827,-1.1493696281
C,0,-3.1493405175,1.293727913,0.484385862
H,0,-3.7818326608,2.160044267,0.7317135402
C,0,-3.9450140651,0.3353753761,-0.3964837799
C,0,-3.1536682006,-0.8696074313,-0.9009275075
H,0,-2.3867032598,-0.4913608096,-1.5950272713
H,0,-3.813171449,-1.5057035255,-1.5108641669
H,0,-4.3108564471,0.8941687021,-1.2690895286
C,0,1.0326478738,2.2023480179,-0.4586229262
O,0,1.4861766115,1.4278015494,0.4500769834
O,0,1.2614346621,2.0619340384,-1.6880043395
C,0,-2.4747644154,-1.7166257763,0.1622776753
H,0,-1.9038597304,-1.0712810981,0.8458091041
H,0,-3.2342709639,-2.2213182244,0.7743554426
H,0,-2.9019527924,0.8101239254,1.4427810264
S,0,-1.3263633921,-2.9731962336,-0.5501788678
S,0,-5.4504579763,-0.1576387878,0.5692564132
H,0,-5.9538064102,-0.9960072602,-0.3631832547
Fe,0,2.5025206261,-0.3736059544,0.2036294018
O,0,0.8816702519,-1.5422785635,1.0329173013
O,0,4.3551417289,0.7766753038,-0.514319167
H,0,4.7333957957,1.2328075133,0.2502306135
H,0,4.0262481756,1.4846266261,-1.0867292105
H,0,0.2397087641,-2.069745161,0.4629604358
H,0,1.1475140819,-2.1295561128,1.7528581467
O,0,3.3000489539,-0.2219631881,2.2956092298
H,0,4.2648905162,-0.2887752476,2.2749987535
H,0,3.0073340904,-0.9886010807,2.8080420507
O,0,1.8516749633,-0.5640716315,-1.9465435233
H,0,1.6533095249,0.4037984819,-2.0182799907
H,0,2.6526240372,-0.6992317928,-2.4707593472

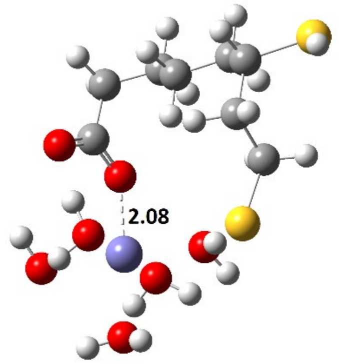

O,0,3.7086901387,-2.1911791609,-0.0629236473
H,0,4.3674291938,-2.0294394264,-0.7540352397
H,0,3.1343401866,-2.8829375657,-0.4236539774

{43} Fe^{2+} – $DHLA^{2-}$ (COO)

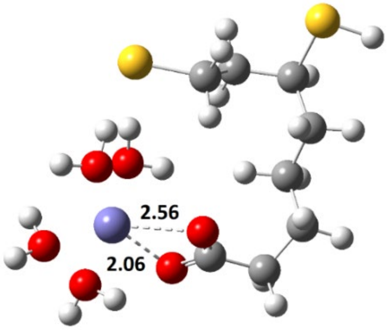

Charge = 0 Multiplicity = 5
C,0,-0.1272504368,3.2800550325,0.1329724782
H,0,-0.5087185105,4.1295764247,-0.4502271825
H,0,-0.2024295841,3.5562616565,1.1919609877
C,0,1.3119912693,2.9654679755,-0.2554759113
H,0,1.9397572605,3.8528283738,-0.101626273
H,0,1.3465223126,2.7437868931,-1.3317840076
C,0,1.8725610575,1.7764863733,0.5206049884
H,0,1.0735751228,1.0325190972,0.6655506494
H,0,2.1788117318,2.0893582323,1.5308148811
C,0,3.034411206,1.1110421961,-0.2030100414
H,0,3.855816881,1.8351253866,-0.3204613463
C,0,3.5583791058,-0.1314831857,0.5027629137
C,0,2.5129176811,-1.2247536871,0.7420720324
H,0,1.8370032777,-0.8766714698,1.5381630378
H,0,3.0153339076,-2.1134423374,1.1522097676
H,0,3.9716213121,0.1543122057,1.4797392045
C,0,-1.0346991325,2.10128072,-0.1254387982
O,0,-0.9068883147,1.413315809,-1.1666232265
O,0,-1.9178393621,1.8076269008,0.7614569127
C,0,1.6817287408,-1.6359804035,-0.4651333097
H,0,1.0992688802,-0.7792215067,-0.8368994104
H,0,2.3369045264,-1.9513531189,-1.2886982854
H,0,2.7149093012,0.8421964384,-1.2222975666
S,0,0.5185826888,-3.0145554716,-0.069894292
S,0,4.9637869936,-0.8815009209,-0.4489112357
Fe,0,-2.5169835911,-0.069267346,0.1603700002
O,0,-2.0299688642,-1.6110327815,-1.1861974221
O,0,-4.1008279568,-0.6231961724,1.5445530231
O,0,-4.1526630827,0.5989890214,-1.2175245036
O,0,-1.0651961251,-0.954869437,1.67636624
H,0,-3.8333527075,1.3154434074,-1.7841583666
H,0,-4.8723439572,0.9882960324,-0.7016171738
H,0,-4.9241112064,-0.8707393632,1.1010804452
H,0,-3.8784817699,-1.3702659812,2.1174654785
H,0,-1.2420999367,-2.1533568704,-0.8785751057
H,0,-2.7441323243,-2.2370318983,-1.3688988538
H,0,-0.4218333068,-0.2680105697,1.9028416896
H,0,-0.5355533824,-1.661888914,1.2096104077
H,0,5.709953295,0.2434942587,-0.5070768257

{44} $[Fe(H_2O)_6]^{3+}$ (low spin)

Charge = 3 Multiplicity = 2
Fe,0,0.527500449,-0.4686395438,-0.0008196237
O,0,0.4300379447,1.5083092018,-0.0773192342
H,0,1.147280739,1.8632040348,-0.6312514772
O,0,-1.3815818875,-0.4866394916,0.1598692291
H,0,-1.7619978437,-1.3197078775,-0.1712249228
O,0,2.4566816988,-0.5055248263,-0.1593612805
H,0,2.8383566769,-1.212937928,0.3905752922
O,0,0.6821650853,-0.5317688608,1.9247033728
H,0,0.1897815933,-1.281140855,2.3059205607
O,0,0.4391156997,-2.4505803258,0.0415842934
H,0,0.6592153379,-2.8490922895,-0.8176979101
O,0,0.4744731998,-0.3219792108,-1.9386724907
H,0,-0.2912473959,0.1990562321,-2.2408068356
H,0,0.53147543,1.9487061898,0.7832545505
H,0,0.4200745944,-1.1757523011,-2.401682614
H,0,2.8566006877,0.3263757526,0.1514412239

```
H,0,1.0888216257,-2.8229638269,0.663186291
H,0,0.3292996978,0.2618334578,2.3643512423
H,0,-1.7954153327,0.2355734681,-0.3460506671
```

{45} Fe^{3+} – $DHLA^{2-}$ (CO, S2 *cis*) (low spin)

```
Charge = 1 Multiplicity = 2
C,0,-0.3144476622,3.0909396107,0.3779425649
H,0,0.3283842518,3.5393620835,1.1468460075
H,0,-0.5449061089,3.8667794162,-0.3616583811
C,0,-1.5894729549,2.5407770117,1.0098011548
H,0,-2.0520311455,3.3203594688,1.6277193207
H,0,-1.3155123315,1.7290067795,1.697381408
C,0,-2.5834162865,2.028423039,-0.0384145169
H,0,-2.0316038267,1.7630507881,-0.9525369335
H,0,-3.2458696194,2.8491329771,-0.3413442583
C,0,-3.4162945108,0.8183588134,0.4119682974
H,0,-4.4797443511,1.0842015395,0.472990168
C,0,-3.2757481318,-0.3756328844,-0.5309470806
C,0,-1.8541855036,-0.9084172647,-0.709432101
H,0,-1.2575559906,-0.1416934762,-1.2262928529
H,0,-1.8865183131,-1.7692373086,-1.3929512351
H,0,-3.6480274202,-0.0797405111,-1.5210242215
C,0,0.4840831192,2.0036373005,-0.3031366034
O,0,0.7970569225,1.0093869158,0.4460108553
O,0,0.7911272622,2.1030439424,-1.5154317853
C,0,-1.1386148901,-1.3112112249,0.5671581879
H,0,-1.0171965832,-0.4691242303,1.2583022623
H,0,-1.6991972775,-2.091385539,1.1004917698
H,0,-3.1226370591,0.514804388,1.4282952902
S,0,0.5044316493,-2.0449012359,0.253962953
S,0,-4.4313852142,-1.6859619353,0.0892498191
H,0,-4.1581784825,-2.591776336,-0.8757009349
Fe,0,2.0106051126,-0.4375671908,0.0286172818
O,0,3.3463840852,-1.9193225947,-0.217621156
O,0,3.5829998246,0.9979026497,-0.0512269444
O,0,1.7950987462,-0.2575922702,-1.8894056299
O,0,2.413480451,-0.4173265365,1.9574510952
H,0,3.2804199175,-2.3517839684,-1.0841795929
H,0,4.2432238294,-1.54869283,-0.1879441574
H,0,4.2285384225,0.7662509639,0.6345852739
H,0,4.0644468756,0.9006774888,-0.8871398523
H,0,1.4712110501,0.6899489295,-1.9813068949
H,0,2.6495463733,-0.3302162984,-2.3438507974
H,0,1.7285392892,0.0726400515,2.4426019582
H,0,2.4495944811,-1.2998595223,2.3605412616
```

{46} $[Fe(H_2O)_6]^{2+}$ (low spin)

```
Charge = 2 Multiplicity = 1
Fe,0,0.5700268851,-0.486454094,0.0461846023
O,0,0.4704875154,1.5368144733,-0.0873310628
H,0,1.3380805554,1.9677640216,-0.0815230065
O,0,-1.4572210878,-0.4714921967,0.1448221651
H,0,-1.8112650419,-1.3701230076,0.0630179831
O,0,2.5993490726,-0.5037487819,0.0001991072
H,0,2.8994788946,-1.2159521027,-0.5849326313
O,0,0.6663460737,-0.3258710744,2.0700937137
H,0,0.6757405047,-1.1783104677,2.5298838217
O,0,0.6536731581,-2.5112510875,0.0928866152
H,0,0.0135630728,-2.8844025823,-0.5318651256
O,0,0.4708987968,-0.6397000542,-1.974987465
H,0,-0.0177707139,0.111583001,-2.3439813786
H,0,0.0015434642,1.9088146928,0.6748502916
H,0,1.3525857203,-0.5847936615,-2.3737112797
H,0,2.9627838656,0.3066426206,-0.3871877128
```

```
H,0,0.4082837263,-2.8810572785,0.9540854898
H,0,-0.126424833,0.1253490614,2.397432847
H,0,-1.8295196289,0.0125235183,-0.6079379742
```

{47} Fe^{2+} – $DHLA^{2-}$ (CO, S2 *cis*) (low spin)

```
Charge = 0 Multiplicity = 1
C,0,-0.124892959,3.2569567282,0.5525088756
H,0,0.3174328701,3.7447223771,1.4326478563
H,0,-0.2181747484,4.0264258337,-0.2240890156
C,0,-1.481910247,2.6604889479,0.8990450076
H,0,-2.1385090928,3.4444832573,1.2999588836
H,0,-1.3510262878,1.9245651007,1.7047491024
C,0,-2.1348079706,1.9823784816,-0.3028874881
H,0,-1.3561418138,1.4392927735,-0.8577748455
H,0,-2.5099222341,2.7433236651,-1.0016928827
C,0,-3.2564722134,1.0117408548,0.0804553053
H,0,-4.2341192431,1.5073186025,0.0051614639
C,0,-3.2783511339,-0.2590985858,-0.7681164668
C,0,-1.9680960677,-1.0506957045,-0.7711821094
H,0,-1.2442410337,-0.5084791269,-1.3967538703
H,0,-2.1300817871,-2.0122208173,-1.280889121
H,0,-3.522115794,0.0057461443,-1.8060448887
C,0,0.8674068872,2.2208535292,0.0671300842
O,0,0.8886489972,1.1018369311,0.6720358261
O,0,1.6242402886,2.5093352754,-0.9068978192
C,0,-1.3435319797,-1.2998283393,0.5912618483
H,0,-1.2118136403,-0.3607352953,1.1408689918
H,0,-1.9961779428,-1.9406916244,1.198950758
H,0,-3.1448272761,0.7243497014,1.1372448366
S,0,0.3006366447,-2.1145591196,0.4883340816
S,0,-4.7002224338,-1.2721165277,-0.1409859426
H,0,-4.5305452019,-2.3101295679,-0.9885576162
Fe,0,1.9616854589,-0.4945807982,0.0807938472
O,0,3.1510423016,-2.0657666809,-0.4996995835
O,0,3.6337740682,0.7781093034,-0.1273961237
O,0,1.4525125822,-0.0956151652,-1.8705227364
O,0,2.4840683119,-0.7822463017,2.0419418127
H,0,2.6046972285,-2.7684148492,-0.8841907685
H,0,3.748488505,-1.7979814343,-1.2140602119
H,0,4.2202824468,0.4699025182,-0.8324663515
H,0,3.1773466875,1.5670489541,-0.4951127864
H,0,1.5484152883,0.8801850307,-1.8952482663
H,0,2.142257707,-0.4378687801,-2.4577550892
H,0,1.7991810485,-1.4476657681,2.2580445277
H,0,3.330748779,-1.2481975239,2.1102828748
```

{48} ASC^-

```
Charge = -1 Multiplicity = 1
C,0,-2.0144392308,0.2404012737,0.5373567622
O,0,-2.661345839,1.2191639054,-0.1869460655
C,0,-1.6684421153,2.1072244744,-0.7318994424
C,0,-0.3268433147,1.5623113928,-0.2725617512
C,0,-0.6220352969,0.4499226441,0.4909584728
O,0,0.3052410347,-0.3613353293,1.1169215273
H,0,-0.1732808803,-1.0509214764,1.6008706384
O,0,0.7740356894,2.0976207008,-0.587289342
O,0,-2.6718843029,-0.6394725792,1.1065702715
H,0,-1.8341045417,3.1054606566,-0.3022452078
C,0,-1.7909223558,2.2027145587,-2.2356088689
H,0,-1.0031415789,2.9029765736,-2.5661785906
C,0,-3.1303138419,2.753983277,-2.6638442068
H,0,-3.9337188235,2.0684270234,-2.3608681184
H,0,-3.2957554496,3.7277969559,-2.1791246341
O,0,-3.0985456482,2.8861903734,-4.0794185571
```

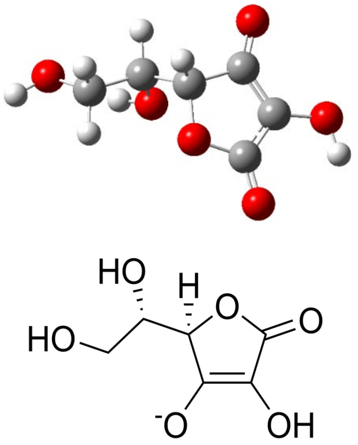

H,0,-4.0036188751,2.9812284963,-4.4021162419
O,0,-1.5611871214,0.9173738269,-2.7922574733
H,0,-1.733922508,0.9775552521,-3.7427871722

{49} Fe^{3+} – LA^{-} (CO) (4-coord)

Charge = 2 Multiplicity = 6
C,0,7.3535965315,0.6179969704,0.0376939207
H,0,7.7753257427,0.0439461304,0.8711381421
H,0,8.1144527763,1.3085019056,-0.3415266576
C,0,6.0683943248,1.3269684448,0.4146924697
H,0,5.798901114,2.0212849625,-0.3928949233
H,0,6.2160169998,1.9234566693,1.324211814
C,0,4.9006120116,0.3566056353,0.6339401103
H,0,4.8234487555,0.0846222781,1.6954116909
S,0,6.9179951344,-0.5292717808,-1.3172179895
S,0,5.3007810514,-1.2740218815,-0.1950150882
C,0,3.5815102549,0.9335971221,0.1547058192
H,0,3.4773796421,1.9354932015,0.6040016516
H,0,3.6351363976,1.0866029383,-0.9358173474
C,0,2.3671221742,0.0960829341,0.5113941483
H,0,2.4505680176,-0.8966123766,0.039579049
H,0,2.3515399315,-0.0797063412,1.5992301476
C,0,1.0600248947,0.7443462521,0.0844269123
H,0,1.0815808794,0.9447789506,-0.9969752406
H,0,0.9509587055,1.7199571203,0.5797760395
C,0,-0.1275296748,-0.1374112674,0.4164413269
H,0,-0.0376757466,-1.1208442069,-0.067143799
H,0,-0.1709112745,-0.3501027022,1.4953321076
C,0,-1.4637536282,0.4183592447,0.0349360035
O,0,-2.4642157679,-0.4078248704,0.2640333294
O,0,-1.6228582549,1.5451423894,-0.4435146562
Fe,0,-4.3244578377,-0.1823206646,-0.0342128781
O,0,-6.3097173459,-0.2230620827,-0.7225776476
O,0,-4.3193500104,1.7177095146,-0.8064208071
H,0,-4.5855478527,1.7471525423,-1.7400434935
H,0,-3.3492755218,1.9307887286,-0.7816891578
H,0,-6.6336694209,-1.1146442698,-0.9264773031
H,0,-6.4831036293,0.3130318452,-1.512092178
O,0,-4.894480245,-1.9346454725,0.9534094448
H,0,-4.2511687608,-2.1823393639,1.6368443122
H,0,-5.7530773681,-1.8575614997,1.3999367273

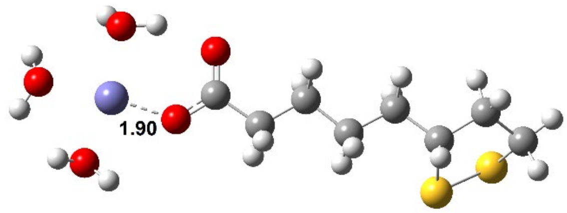

{50} Fe^{3+} – LA^{-} (CO) (5-coord)

Charge = 2 Multiplicity = 6
C,0,7.3822372127,0.6601452978,0.347891191
H,0,7.5842756076,0.4307492852,1.4009905319
H,0,8.2239795987,1.2274236417,-0.0626689985
C,0,6.0583291117,1.3694873185,0.1690849586
H,0,5.9352749345,1.6599083846,-0.8838753374
H,0,6.0302737979,2.2860531631,0.7737722533
C,0,4.8992027119,0.4749000308,0.5859292756
H,0,4.8238395569,0.4522790266,1.6818183057
S,0,7.2677155871,-0.8915329115,-0.610864957
S,0,5.313826317,-1.2927209359,0.1129760735
C,0,3.573524771,0.9061486435,-0.0108232606
H,0,3.4250740476,1.9659142803,0.2554675317
H,0,3.6422572141,0.8634963028,-1.1095222225
C,0,2.3802289174,0.0986679399,0.4710878182
H,0,2.4885263716,-0.9515237767,0.152777267
H,0,2.3655267289,0.0879080685,1.5729104989
C,0,1.0612833568,0.647501292,-0.0484170634
H,0,1.0925087978,0.7065650076,-1.1465944744
H,0,0.9259412266,1.6766735588,0.3135721444
C,0,-0.1143703432,-0.2075875896,0.3825151503

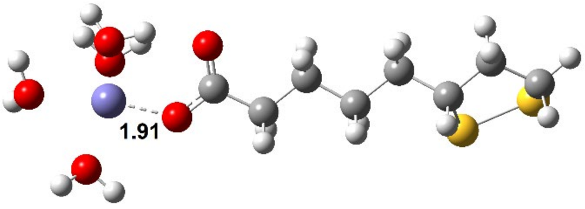

H,0,-0.0193348491,-1.2358732947,0.0068252335
H,0,-0.1469650791,-0.3028755415,1.4789107872
C,0,-1.4580047498,0.3088712915,-0.0345252541
O,0,-2.454376801,-0.502935064,0.2308331859
O,0,-1.6172266028,1.4163317104,-0.5631520466
Fe,0,-4.303551675,-0.2524605303,-0.1862867797
O,0,-6.4718895293,-0.0453824657,-0.2792135718
O,0,-4.304068244,1.7962152294,-0.383597222
H,0,-4.774464373,2.0572081378,-1.1922602238
H,0,-3.3403713955,1.9669480565,-0.5441963649
H,0,-6.857236337,-0.7910058178,-0.7653511268
H,0,-6.7459251125,0.7512124016,-0.7588729903
O,0,-4.1957107386,-0.7355998163,-2.1935500169
H,0,-4.8918082817,-0.2630248129,-2.6799787511
H,0,-3.3501678568,-0.4169479849,-2.5529770281
O,0,-4.9149384214,-2.1311006405,0.5349840674
H,0,-4.1724376691,-2.5863757464,0.9635291696
H,0,-5.6148638088,-2.0707631402,1.2050572462

{51} Fe^{3+} – LA^{-} (COO) (4-coord)

Charge = 2 Multiplicity = 6
C,0,-6.7693143888,1.0161481303,0.033774298
H,0,-7.0145489672,1.11072511,-1.0309385373
H,0,-7.5262754716,1.5436863296,0.6234457916
C,0,-5.3633769328,1.4869665794,0.3347507924
H,0,-5.1917646872,1.4583304555,1.4199921215
H,0,-5.2271517032,2.5247135979,0.0017863712
C,0,-4.3302593623,0.6122966324,-0.3633626517
H,0,-4.2528982078,0.9024325979,-1.4201908769
S,0,-6.8327974851,-0.7431690463,0.5241048848
S,0,-4.9783529177,-1.147872822,-0.4191486954
C,0,-2.9609271213,0.6780298667,0.2853281934
H,0,-2.6805784801,1.7424934322,0.3505955692
H,0,-3.031345212,0.3056125415,1.3195229689
C,0,-1.8857651832,-0.0748224921,-0.478324132
H,0,-2.1129398449,-1.1536138631,-0.4810790018
H,0,-1.8986350805,0.2461595051,-1.5323777338
C,0,-0.4934714955,0.1417343181,0.0919283099
H,0,-0.4613968369,-0.1803320735,1.1421860874
H,0,-0.2562302286,1.2152960334,0.0837409842
C,0,0.5431790527,-0.6164885995,-0.71769294
H,0,0.3782811202,-1.7035081622,-0.6580245317
H,0,0.4761058722,-0.3716108504,-1.7868773287
C,0,1.950562091,-0.3925521539,-0.3220740892
O,0,2.9151435003,-0.7721905582,-1.0843609526
O,0,2.3072130901,0.1759931905,0.7627735082
Fe,0,4.2528684786,-0.0191712093,0.1775937476
O,0,5.5779163177,-1.4086436711,0.7811971768
O,0,4.8644545334,1.8875561102,-0.062767987
H,0,4.1878195537,2.512364616,0.2488944654
H,0,5.0357334556,2.1119865407,-0.9927420184
H,0,5.1558477917,-2.2517657511,1.0162677233
H,0,6.1009987497,-1.1387713346,1.554435483

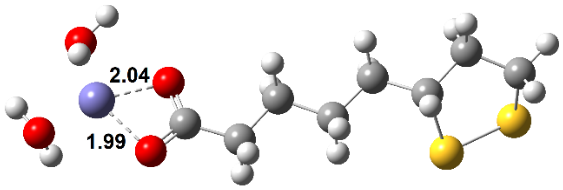

{52} Fe^{3+} – LA^{-} (COO) (5-coord)

Charge = 2 Multiplicity = 6
C,0,-6.8158208253,1.2223472444,-0.2481627173
H,0,-6.9532363175,1.5046793638,-1.2965657471
H,0,-7.5857413814,1.7106790158,0.355892718
C,0,-5.4184183542,1.5542823104,0.232210034
H,0,-5.3792306025,1.5690890139,1.330144893
H,0,-5.1376967436,2.5541717881,-0.1268489168
C,0,-4.4217595736,0.5325475783,-0.2847806796
H,0,-4.426009965,0.5218738491,-1.3854009232

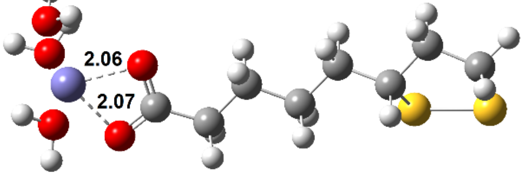

```
S,0,-7.081316073,-0.6097595837,-0.1391515953
S,0,-5.0581626051,-1.1056633857,0.2858676627
C,0,-3.0119646251,0.7533363695,0.2285052186
H,0,-2.7056759829,1.7568059663,-0.1067695226
H,0,-3.0243267737,0.7822200722,1.3297403216
C,0,-1.9944848045,-0.2663945645,-0.2581418073
H,0,-2.1990894989,-1.2528357796,0.1885323374
H,0,-2.0946052942,-0.3928731211,-1.3480866302
C,0,-0.5718938386,0.1511236871,0.074444712
H,0,-0.4740518438,0.3063542967,1.1586104014
H,0,-0.3593113453,1.1191739987,-0.400889706
C,0,0.4420112134,-0.8783704185,-0.3898791745
H,0,0.2945478605,-1.8478898136,0.1072592452
H,0,0.3345918701,-1.0856140016,-1.4653213148
C,0,1.8599999001,-0.4966008275,-0.1836805884
O,0,2.8199845962,-1.2697619796,-0.5224573238
O,0,2.2118260873,0.6258692384,0.3181003018
Fe,0,4.1989270038,0.159618774,0.044254482
O,0,4.7509495233,2.1394574783,0.5555031305
H,0,4.6784509377,2.2471102907,1.5176936796
H,0,4.1163486993,2.7671635886,0.172882539
O,0,4.7116386359,-0.6319677787,1.8468683618
O,0,4.5811950746,0.7324249682,-1.8935962889
H,0,5.647914526,-0.4963016921,2.0672231748
H,0,4.2018604256,-0.2101075319,2.5588581198
H,0,5.4124892889,1.2264032528,-1.9824180772
H,0,4.6385148056,-0.0161196669,-2.5096503201
```

{53} Fe^{3+} – $DHLA^{2-}$ (CO, S1) (5-coord)

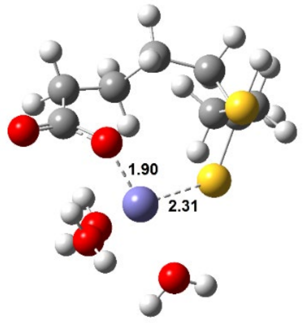


```
Charge = 1 Multiplicity = 6
C,0,-1.3887271446,2.8998337736,0.7553181151
H,0,-2.4199577211,3.2220506262,0.5573769682
H,0,-0.9963118775,3.6095689105,1.4993519683
C,0,-0.5467661262,2.9104209215,-0.5078424454
H,0,-0.7247838049,3.8499533333,-1.048512609
H,0,-0.8884780038,2.1110193703,-1.1795403384
C,0,0.9525935988,2.7604481793,-0.2474942595
H,0,1.126357505,2.1305363009,0.6329573364
H,0,1.3576698079,3.747868262,0.0156183017
C,0,1.7297905488,2.2247196633,-1.4474672344
H,0,2.7648525038,2.5969140296,-1.3995823153
C,0,1.8934830036,0.7173027256,-1.6748147465
C,0,2.7900143336,-0.0449625031,-0.6910197004
H,0,3.6873902474,0.5768448311,-0.5403998108
H,0,3.1300096264,-0.9617529299,-1.1924477203
H,0,2.4250654994,0.6501534266,-2.6336072682
C,0,-1.4879868042,1.5612123709,1.4470401741
O,0,-0.7700520702,0.5910327335,0.9576353489
O,0,-2.2054067801,1.414765293,2.4437343093
C,0,2.2292014737,-0.4356774159,0.663427678
H,0,1.9532613583,0.4270629889,1.2754844314
H,0,1.3348279144,-1.0676364549,0.5622493224
H,0,1.3029939319,2.6589199721,-2.3647031811
S,0,3.4033243273,-1.4728066428,1.6190302191
H,0,4.421073508,-0.5827063883,1.6115632462
S,0,0.3598082427,-0.2543313855,-2.1067071645
Fe,0,-1.1298397607,-0.6889853413,-0.4002903705
O,0,-2.2454690603,-1.7585612358,1.1821818871
O,0,-1.4175432589,-2.6406165224,-1.2500426593
O,0,-2.8804781378,0.1122894634,-1.2432684978
H,0,-2.8741597585,1.0829592398,-1.2139832591
H,0,-3.6287617773,-0.1590558588,-0.6867908172
H,0,-2.0343921687,-1.3379758205,2.0294109385
H,0,-2.0069299071,-2.69155563,1.2827229405
H,0,-1.0420776994,-2.8151044137,-2.1260967994
```

H,0,-2.3583485697,-2.8678798723,-1.3122319882

{54} Fe^{3+} – $DHLA^{2-}$ (CO, S2) (4-coord)

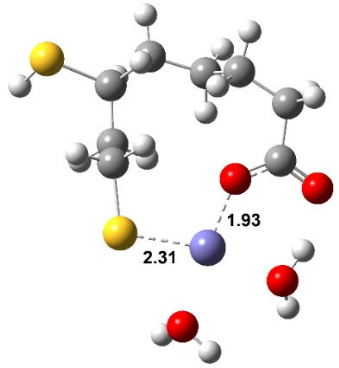


```
Charge = 1 Multiplicity = 6
C,0,-0.340670096,3.3020505229,0.574354263
H,0,0.0256964162,3.9537215706,1.379921914
H,0,-0.4991182874,3.9643252487,-0.2881791339
C,0,-1.6242655036,2.6003062355,0.9768158226
H,0,-2.3252183773,3.3405136643,1.3849210666
H,0,-1.4063990505,1.8994091954,1.7949751953
C,0,-2.2668909266,1.8459584929,-0.1832987829
H,0,-1.468565379,1.3668507229,-0.7640117394
H,0,-2.7422366319,2.5585151258,-0.8720801588
C,0,-3.2796704389,0.7916214322,0.2681823066
H,0,-4.2890753915,1.2232655638,0.3028482433
C,0,-3.3018068614,-0.4487717832,-0.6229192169
C,0,-1.9516005187,-1.1582221125,-0.765479599
H,0,-1.3039631853,-0.5420204368,-1.4058045471
H,0,-2.0954391777,-2.1023719172,-1.3088492215
H,0,-3.638429393,-0.1635096707,-1.6285531593
C,0,0.8204293231,2.4062715371,0.222139706
O,0,0.7157484233,1.1446632494,0.5094352847
O,0,1.834707495,2.9068464783,-0.2989149883
C,0,-1.2386170741,-1.426786716,0.5509469908
H,0,-1.112010247,-0.5122062196,1.1379691965
H,0,-1.7975896243,-2.1461776569,1.1612801608
H,0,-3.0513789124,0.4820192293,1.299933806
S,0,0.4281126809,-2.1579442572,0.2852132311
S,0,-4.6020066672,-1.5703778839,0.0713607647
H,0,-4.5230266653,-2.5072463715,-0.8988916269
Fe,0,1.8696754049,-0.3585987021,0.1285889266
O,0,3.2582709499,-1.9255045916,-0.4036379475
O,0,3.5730412072,0.9181244823,-0.0560403479
H,0,3.022718663,-2.4692399685,-1.1706244334
H,0,4.1682093229,-1.6305025967,-0.5592812719
H,0,4.1023206746,0.7203120337,-0.8421721421
H,0,3.1058608479,1.7781470992,-0.2324565617
```

{55} Fe^{3+} – $DHLA^{2-}$ (CO, S2) (5-coord)

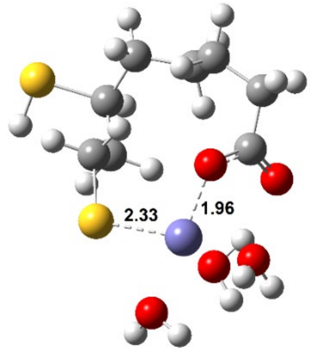


```
Charge = 1 Multiplicity = 6
C,0,-0.3811022733,3.2196913035,0.5146967316
H,0,0.1913599352,3.6506187467,1.3466695225
H,0,-0.6212093942,4.029969152,-0.1836293201
C,0,-1.6492555619,2.5500912288,1.0393353018
H,0,-2.2190330536,3.2717617938,1.6381395315
H,0,-1.3598826096,1.742432348,1.7255798241
C,0,-2.5143966012,1.9866913597,-0.0895848957
H,0,-1.8571261944,1.6646495852,-0.9106528703
H,0,-3.1252180446,2.7913458369,-0.5194323203
C,0,-3.4058300194,0.8089262391,0.3292563096
H,0,-4.458383791,1.117829079,0.3756508042
C,0,-3.2907121729,-0.3862684097,-0.6165788399
C,0,-1.8811206966,-0.9631443499,-0.7581728448
H,0,-1.2595533071,-0.2433022005,-1.3126972945
H,0,-1.9252885294,-1.8626034507,-1.3886383268
H,0,-3.630007762,-0.0822732264,-1.6158373683
C,0,0.510358381,2.2297007669,-0.1933861624
O,0,0.8268305218,1.1748802311,0.4949439125
O,0,0.8862550334,2.4197380929,-1.3663276946
C,0,-1.1956476674,-1.2937167511,0.5551111596
H,0,-1.0586333523,-0.4016817608,1.1749945434
H,0,-1.7797533955,-2.0203588837,1.1327227223
H,0,-3.1431903999,0.4836465889,1.3474633308
S,0,0.4512767446,-2.0555527922,0.2929490504
```

```
S,0,-4.4965484111,-1.6572482157,-0.0124020158
H,0,-4.17232582,-2.6122743912,-0.9119012199
Fe,0,1.9479679333,-0.3199305942,-0.1061236525
O,0,3.3835097587,-1.943177065,-0.2715483114
O,0,3.7295648425,0.9649259689,0.0237647879
O,0,1.7151831385,-0.051149386,-2.1645974773
H,0,3.1745479426,-2.5663428875,-0.9843354181
H,0,4.2644338961,-1.5979067358,-0.4842266056
H,0,4.4250425512,0.5561369031,0.5607552722
H,0,4.1004324268,1.0203447536,-0.8706893143
H,0,1.4553162328,0.9069524134,-2.1483690871
H,0,2.598715719,-0.0825842911,-2.5653277648
```

{56} Fe^{3+} – $DHLA^{2-}$ (COO, S2) (4-coord)

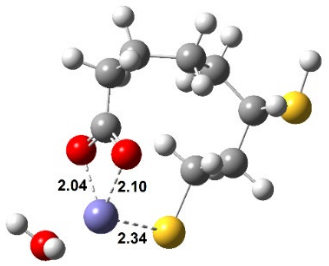


```
Charge = 1 Multiplicity = 6
C,0,0.1769138412,3.0725956005,0.0842238618
H,0,-0.3039319821,3.8752215879,-0.4896446998
H,0,0.3032458856,3.4194229595,1.115507587
C,0,1.5289697182,2.7197189811,-0.5590553935
H,0,1.9980644482,3.6493806842,-0.9004403568
H,0,1.3380113783,2.1340410536,-1.4690353078
C,0,2.4829103266,1.9616919578,0.377097881
H,0,1.9036442065,1.4791945876,1.1745957017
H,0,3.1152174023,2.6877850149,0.9039057239
C,0,3.3821459983,0.9202996152,-0.3240675965
H,0,4.4028220885,1.3153587056,-0.3938382767
C,0,3.4246772893,-0.4433647636,0.374784931
C,0,2.243668272,-1.3640656481,0.0420552878
H,0,2.3273444927,-2.2936782833,0.6214180017
H,0,2.2903634436,-1.6357721307,-1.024006476
H,0,3.4743036289,-0.2979572426,1.4630883132
C,0,-0.7740260459,1.920482608,0.0661603416
O,0,-1.133094276,1.3972720355,-1.0474250495
O,0,-1.2438457884,1.3803797013,1.1203340148
C,0,0.9195784198,-0.6855175979,0.324065811
H,0,0.8434123846,-0.3877092507,1.3762016494
H,0,0.8395442838,0.2016111431,-0.3046592484
H,0,3.0524614363,0.7583403927,-1.3629025662
S,0,-0.5376100357,-1.7289791864,-0.0592467248
S,0,4.9265357914,-1.4091145844,-0.1108517434
Fe,0,-2.1816750557,-0.0579960783,-0.0815465442
O,0,-4.184291963,0.3783640932,-0.067922297
H,0,-4.3542922765,1.1984808538,-0.5587123392
H,0,-4.5228392409,0.530382397,0.8286257933
H,0,5.8323249278,-0.4829102068,0.2717507204
```

{57} Fe^{3+} – $DHLA^{2-}$ (COO, S2) (5-coord)

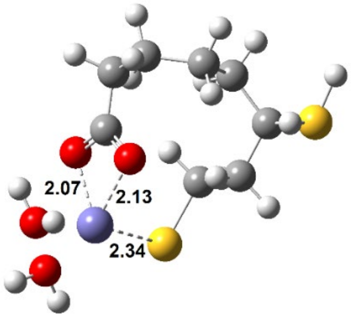


```
Charge = 1 Multiplicity = 6
C,0,0.0171469095,2.9312660317,-0.0989369799
H,0,-0.4711844952,3.663485196,-0.752686152
H,0,0.0923895561,3.3595811991,0.9068680182
C,0,1.4122233277,2.5929970072,-0.6547368532
H,0,1.8598724165,3.5177664851,-1.0368899586
H,0,1.2896911126,1.9404482993,-1.5310746325
C,0,2.3531732163,1.9449667269,0.3715546676
H,0,1.7629706739,1.4379137254,1.1447127332
H,0,2.8836574051,2.7394498022,0.9120619054
C,0,3.381319117,0.9652768562,-0.2362999499
H,0,4.3798459124,1.4154898387,-0.1787537668
C,0,3.4332208631,-0.4139917894,0.4315998216
C,0,2.3226373808,-1.3838279057,0.0075453857
H,0,2.439957059,-2.3325103732,0.5491385845
H,0,2.4243598072,-1.6034533306,-1.0667391926
H,0,3.4053685487,-0.2945649664,1.5240573581
```

C,0,-0.8670948653,1.7259170678,-0.053735177
O,0,-1.3159666217,1.2074404655,-1.1373042554
O,0,-1.176317286,1.1387109964,1.0305224819
C,0,0.9564736644,-0.7888945404,0.2746705798
H,0,0.8523343483,-0.502842582,1.3269260425
H,0,0.8388786047,0.0995414118,-0.345363944
H,0,3.1827609978,0.8141364091,-1.3095351207
S,0,-0.4533667026,-1.8826187805,-0.1394971519
S,0,5.0124029675,-1.2890813855,0.0231229036
Fe,0,-2.2143898386,-0.3447019633,-0.0951043323
O,0,-3.1585623827,-1.3112328463,-1.6920056429
O,0,-4.0876365718,0.7945387117,0.300628107
H,0,-3.9316618247,1.6773529478,-0.0694278722
H,0,-4.15545532,0.9338420862,1.2570577876
H,0,-3.0807907872,-2.273485576,-1.7780313181
H,0,-4.1095334528,-1.1188612249,-1.712835098
H,0,5.8403582602,-0.3252119997,0.4817050216

{58} Fe^{2+} – LA^{-} (CO) (4-coord)
Charge = 1 Multiplicity = 5
C,0,-6.873885174,0.4640637955,-0.4026241327
H,0,-7.1946586562,-0.094430962,-1.2900703826
H,0,-7.6790204281,1.1384222376,-0.0923991004
C,0,-5.5645739047,1.1916710552,-0.6237125784
H,0,-5.3621156454,1.8327644605,0.2454886684
H,0,-5.6323725344,1.8424516017,-1.5053779155
C,0,-4.3935981918,0.2260205553,-0.809664602
H,0,-4.3065227384,-0.0624277594,-1.8661490026
S,0,-6.5724432376,-0.7035264819,0.9702559535
S,0,-4.8020984576,-1.3906506117,0.0501779929
C,0,-3.0793248179,0.8086074303,-0.325518403
H,0,-2.9577571684,1.7930974111,-0.8078968932
H,0,-3.1510033876,0.9985474637,0.7578679161
C,0,-1.8662958662,-0.0522012414,-0.6297623906
H,0,-1.9669175034,-1.0249863526,-0.1205753971
H,0,-1.8365390667,-0.2721304492,-1.7094351021
C,0,-0.5587486451,0.6009846067,-0.2108533897
H,0,-0.6009286789,0.8591037915,0.8583006549
H,0,-0.4329556136,1.5492231773,-0.7529037971
C,0,0.6284130447,-0.3064239117,-0.4705160934
H,0,0.5192410765,-1.2563335838,0.0707474103
H,0,0.6697512995,-0.5835166036,-1.5357245387
C,0,1.9824314085,0.2637552854,-0.1337923936
O,0,2.9499485594,-0.5860355874,-0.14587175
O,0,2.1235100677,1.4822627169,0.1099037068
Fe,0,4.8845351626,-0.2317025136,0.1450253821
O,0,6.8401095098,0.0506751225,0.9399583086
O,0,4.7835229783,1.9157638699,-0.1157219284
H,0,5.1952651801,2.3395824012,0.6506856089
H,0,3.8047903162,1.9938973011,0.015772976
H,0,7.1908397327,-0.7269509029,1.3976096788
H,0,6.880411455,0.7731658716,1.5835483638
O,0,5.50542101,-2.2102013708,-0.4590878425
H,0,4.8883603631,-2.5562584228,-1.1203009118
H,0,6.3739695521,-2.2170314001,-0.8864920757

{59} Fe^{2+} – LA^{-} (CO) (5-coord)
Charge = 1 Multiplicity = 5
C,0,-7.0786809778,0.6802421703,0.0139854451
H,0,-7.4049653913,0.3653720845,-0.9845138426
H,0,-7.8683861005,1.2830610949,0.4743758353
C,0,-5.7466875778,1.396015188,-0.0249390104
H,0,-5.5012184792,1.7683538689,0.9797576853
H,0,-5.7955722163,2.261516636,-0.6992969743

```
C,0,-4.6385694115,0.4672299403,-0.5033322146
H,0,-4.6842155055,0.3670925318,-1.5965052535
S,0,-6.8379424934,-0.7890130364,1.0738350579
S,0,-4.996422365,-1.2612711806,0.1331427106
C,0,-3.2569227964,0.9321461748,-0.08650401
H,0,-3.1431672594,1.9728523211,-0.4333307398
H,0,-3.2046987356,0.9605425346,1.0137128683
C,0,-2.1182809231,0.0939074971,-0.6425640247
H,0,-2.1879778638,-0.9349442996,-0.2514916495
H,0,-2.2218589865,0.0154336377,-1.7371171823
C,0,-0.7541761823,0.6709962912,-0.3002751774
H,0,-0.6698732741,0.7847764819,0.7911666877
H,0,-0.6731704906,1.683557136,-0.7216738826
C,0,0.3812381275,-0.1942637734,-0.813521197
H,0,0.3111392268,-1.2112089311,-0.4017939962
H,0,0.3044315462,-0.3179730549,-1.9047968913
C,0,1.7758995708,0.3148614333,-0.5307612488
O,0,2.7335164759,-0.473407728,-0.830154922
O,0,1.9367227731,1.4648072363,-0.041498831
Fe,0,4.7062526862,-0.1986718393,-0.1389051377
O,0,6.8239095697,0.0061164405,0.5840807455
O,0,4.5600087938,1.9241625766,-0.2631145921
H,0,4.9766341243,2.3369105159,0.5063487279
H,0,3.5761671282,1.9673091942,-0.1165772129
H,0,6.8250830774,-0.026236272,1.5510655634
H,0,7.1646243696,0.8842988379,0.3640446678
O,0,4.0925241249,-0.6494315512,1.8896323974
H,0,4.618185187,-0.2140148599,2.5752580719
H,0,3.176531435,-0.3814021718,2.0508906137
O,0,5.2978855233,-2.1491581269,-0.7964564781
H,0,4.6848772624,-2.5043883243,-1.4567378694
H,0,6.1664370279,-2.1421736745,-1.2240327396
```

{60} Fe^{2+} – LA^- (COO) (4-coord)

```
Charge = 1 Multiplicity = 5
C,0,-6.1930682885,-0.4955623207,-0.7957756421
H,0,-6.7279589225,-0.9919879215,0.0224139293
H,0,-6.8328901193,-0.4816828883,-1.6846491915
C,0,-4.8416343914,-1.1234160163,-1.0690788077
H,0,-4.4412495941,-0.703299377,-2.0019977537
H,0,-4.9482197198,-2.2060066859,-1.215877236
C,0,-3.8317178957,-0.8646120504,0.0561321992
H,0,-3.8469452724,-1.6898541927,0.7809848634
S,0,-5.8574754325,1.2380783158,-0.3241365452
S,0,-4.4107098541,0.602297227,1.0669555756
C,0,-2.4243284423,-0.6700703135,-0.4760819705
H,0,-2.2082598593,-1.5119203883,-1.1552717853
H,0,-2.4032772505,0.2412135309,-1.0965617727
C,0,-1.3490834166,-0.602735808,0.5934648902
H,0,-1.5592027605,0.2347292119,1.2789776
H,0,-1.3834834579,-1.5188216729,1.2050925521
C,0,0.0416475689,-0.4313906551,0.004423115
H,0,0.0671138244,0.4808222502,-0.6096790343
H,0,0.2575537813,-1.2676445807,-0.6767153762
C,0,1.1117685501,-0.3533436158,1.0769832294
H,0,0.8985709013,0.4642271379,1.7813750005
H,0,1.1219449225,-1.2665947754,1.6899971958
C,0,2.510065837,-0.1407063106,0.5791031267
O,0,3.45919972,-0.0236349068,1.425856554
O,0,2.7688371041,-0.0673728668,-0.660875991
Fe,0,4.8662180545,0.2219496574,-0.1669344765
O,0,5.4733594148,2.1293500762,-0.7614921462
O,0,5.5633749404,-1.7372442633,-0.4271824827
H,0,4.8514941805,-2.371522995,-0.2537438245
H,0,6.2695544262,-1.9669260598,0.1946648896
```

H,0,4.8351909631,2.8106492372,-0.5023368465
H,0,5.558592488,2.2080770205,-1.723315838

{61} Fe^{2+} – LA^{-} (COO) (5-coord)

```
Charge = 1 Multiplicity = 5
C,0,6.4825523454,1.3006918187,0.2056017527
H,0,6.6252900734,1.5674676393,1.2573323314
H,0,7.2334930519,1.8183427583,-0.3978755561
C,0,5.0717677011,1.6080149732,-0.2528693189
H,0,5.0212940699,1.6516740658,-1.349527586
H,0,4.7688302016,2.5901968535,0.135768627
C,0,4.1058185135,0.5476340187,0.2443451968
H,0,4.1192902126,0.5077195107,1.3442564055
S,0,6.7903789128,-0.5230389903,0.0634050086
S,0,4.7807781103,-1.0575291154,-0.3743281319
C,0,2.6865726621,0.7437362901,-0.2530936097
H,0,2.355194414,1.7290802949,0.1110393003
H,0,2.6906442084,0.8030328236,-1.3532816068
C,0,1.7008920908,-0.3169548023,0.2110949432
H,0,1.9364548686,-1.2856215423,-0.2594911901
H,0,1.8103345261,-0.4674038392,1.2973602781
C,0,0.2627873817,0.0585079043,-0.1068219815
H,0,0.1588059705,0.2370500641,-1.1873521179
H,0,0.0210715821,1.0101987114,0.3885152307
C,0,-0.716322958,-1.0124634244,0.3359206381
H,0,-0.5127295995,-1.96480556,-0.1752429773
H,0,-0.5936867692,-1.2291262173,1.4075330016
C,0,-2.1708160463,-0.7042398217,0.1250161456
O,0,-3.0458158473,-1.540425257,0.4879661236
O,0,-2.5277869121,0.4058881438,-0.4040939797
Fe,0,-4.5624970216,0.0851171005,-0.1967253231
O,0,-5.3324649637,1.9842949235,-0.8580674002
H,0,-4.7226303657,2.4409645205,-1.4542448944
H,0,-5.4765617253,2.5959022943,-0.1219960246
O,0,-5.7110951097,-1.1868674497,-1.3914062349
O,0,-4.9214433845,0.6562832831,1.8304902512
H,0,-6.304582494,-0.7145085132,-1.9936638365
H,0,-5.1542000087,-1.7447932947,-1.9540570811
H,0,-5.644844135,1.2889921846,1.9420975204
H,0,-5.1170115564,-0.0755713494,2.4317150959
```

{62} Fe^{2+} – $DHLA^{2-}$ (CO, S1) (5-coord)

```
Charge = 0 Multiplicity = 5
C,0,-1.6809490833,2.4148255484,0.8694385768
H,0,-2.743095977,2.440072939,0.5805906285
H,0,-1.5387727453,3.2670268248,1.5476439689
C,0,-0.8009518855,2.5594361028,-0.3587743169
H,0,-1.0633656551,3.4974208606,-0.8693235153
H,0,-1.0287713697,1.770915823,-1.0894896006
C,0,0.6907704786,2.5674607362,-0.0356561613
H,0,0.9126646884,1.797353776,0.7141702634
H,0,0.9263449982,3.5240947886,0.454086744
C,0,1.6039208426,2.4053384192,-1.250949969
H,0,2.570112742,2.8866497096,-1.030311483
C,0,1.9626591673,1.0078269318,-1.7718594686
C,0,2.9024473769,0.1866079079,-0.8769615924
H,0,3.7190830104,0.858063355,-0.562723955
H,0,3.3655966883,-0.5914121271,-1.500919792
H,0,2.5624750268,1.2032402051,-2.6719469077
C,0,-1.5254973941,1.1616702175,1.7114034116
O,0,-1.0443503624,0.0805223228,1.1975125916
O,0,-1.9123815386,1.2017451005,2.8969800881
C,0,2.3406246799,-0.4946484478,0.3585213051
H,0,1.9036394464,0.2048496226,1.0759706036
```

```
H,0,1.5674354082,-1.2293252607,0.0944239023
H,0,1.171253937,2.9747816577,-2.0887474253
S,0,3.6089465425,-1.4880242995,1.2404103151
H,0,4.4349835151,-0.453959731,1.5140121053
S,0,0.5495962048,0.0175731391,-2.4810538706
Fe,0,-0.8089387083,-0.6185872126,-0.6858044315
O,0,-3.2476358994,-1.6728188743,1.4954653161
O,0,-0.6390635765,-2.7408149064,-0.3811500168
O,0,-2.9316231427,-0.7355811349,-1.1008406926
H,0,-3.3140674662,0.1493228933,-1.1818550761
H,0,-3.2652227844,-1.0949170799,-0.2469744942
H,0,-2.3912717665,-1.221095367,1.6344046786
H,0,-3.0428736105,-2.6162773991,1.5236758383
H,0,0.2328804915,-3.1084754837,-0.584199805
H,0,-1.2634702796,-3.2406615575,-0.9263737633
```

{63} Fe^{2+} – $DHLA^{2-}$ (CO, S2) (4-coord)

```
Charge = 0 Multiplicity = 5
C,0,-0.3459715564,3.2630155808,-0.2369135609
H,0,-0.774292557,3.9349832923,-0.9944127998
H,0,-0.3092438555,3.8504255213,0.6910001413
C,0,1.0402708666,2.7965909877,-0.6462692431
H,0,1.6506160725,3.6674419717,-0.9219301946
H,0,0.9571203092,2.1804227476,-1.5529675587
C,0,1.7286212892,1.9902869001,0.4509437947
H,0,0.9781605247,1.3334768439,0.9092126868
H,0,2.0629624507,2.6625216497,1.2543180217
C,0,2.9010247287,1.1481179081,-0.0545753684
H,0,3.8344646163,1.7262042935,-0.0059652403
C,0,3.0785233074,-0.1571900845,0.7188120026
C,0,1.8500213006,-1.0713838312,0.7157933996
H,0,1.0919893007,-0.6283037493,1.37927902
H,0,2.1156549074,-2.0329518109,1.1789116219
H,0,3.3275912338,0.0766831001,1.7629475485
C,0,-1.3705854661,2.1657316411,-0.0311688732
O,0,-1.1195114885,1.0072740996,-0.5128820925
O,0,-2.4314447358,2.4583181081,0.5770321498
C,0,1.2215606073,-1.3194314794,-0.6450539481
H,0,0.9684452986,-0.3756473593,-1.139558224
H,0,1.9227148178,-1.8522479304,-1.299610612
H,0,2.7491833101,0.9103713143,-1.1186431317
S,0,-0.3105995744,-2.3419341136,-0.5407896173
S,0,4.5689047471,-0.9945379336,0.0003279385
H,0,4.5278940292,-2.0827196805,0.7994148599
Fe,0,-2.0373019774,-0.7835168316,-0.1826078398
O,0,-3.2561545928,-2.5049837869,0.4288922907
O,0,-3.9176790265,0.302747157,0.0524325566
H,0,-2.7946042536,-3.1381799721,0.9972326004
H,0,-4.0902699134,-2.3116687462,0.8799973222
H,0,-4.4318238168,-0.0087347909,0.8093901089
H,0,-3.5363219037,1.1806579834,0.3189132404
```

{64} Fe^{2+} – $DHLA^{2-}$ (CO, S2) (5-coord)

```
Charge = 0 Multiplicity = 5
C,0,0.0040855299,3.3141681188,-0.4199736464
H,0,-0.3340886987,3.9860838042,-1.2219299546
H,0,0.0175063777,3.9144079657,0.4988976909
C,0,1.3837046878,2.7549068134,-0.733070964
H,0,2.0886956828,3.5837292836,-0.8838069003
H,0,1.3413084195,2.2073312586,-1.6853071997
C,0,1.8865301113,1.8181486067,0.360988386
H,0,1.0490009259,1.1786927903,0.6733965558
H,0,2.1681946076,2.395813292,1.2537186839
C,0,3.0523589497,0.939802293,-0.0887193853
```

H,0,3.9988398806,1.491434385,0.0033099126
C,0,3.15724707,-0.371602689,0.6874314958
C,0,1.8940042572,-1.2356652375,0.6514001076
H,0,1.1371308885,-0.7568082744,1.291022477
H,0,2.1056306265,-2.204263017,1.1276687159
H,0,3.3895271091,-0.1491717852,1.7378112069
C,0,-1.0641326057,2.2531087765,-0.2605986838
O,0,-0.9544931752,1.1825773896,-0.9474589811
O,0,-2.0178946422,2.4718784206,0.5382075704
C,0,1.2955395237,-1.4688610279,-0.7260262073
H,0,1.1333044656,-0.520619075,-1.2514981016
H,0,1.977369684,-2.0659239422,-1.3445537256
H,0,2.9394548013,0.7098630792,-1.1594713595
S,0,-0.3183275468,-2.3649551472,-0.6522995502
S,0,4.6305568299,-1.267541105,0.0045081376
H,0,4.5280362008,-2.3509987546,0.8044491813
Fe,0,-1.8422593551,-0.5955205149,-0.304989894
O,0,-3.4567612558,-2.2252117708,0.1205399058
O,0,-3.8148162433,0.6285940682,-0.538445603
O,0,-1.8755436732,-0.0500009568,1.8707614218
H,0,-3.0459198143,-3.0660524931,0.3668127471
H,0,-3.9363362659,-1.9450676293,0.9132222825
H,0,-4.4549907577,0.2617797443,0.0860041345
H,0,-3.3986046479,1.3917655446,-0.0759463958
H,0,-1.9966400908,0.9076987534,1.7083980792
H,0,-2.7100668564,-0.3513959677,2.2568008598

{65} Fe^{2+} – $DHLA^{2-}$ (COO, S2) (4-coord)

Charge = 0 Multiplicity = 5
C,0,-0.6548279719,2.8436953989,0.0868780458
H,0,-1.210616963,3.5827184722,-0.5047968148
H,0,-0.5960912404,3.2137742739,1.1173714414
C,0,0.7473149515,2.6624617528,-0.5091413278
H,0,1.1447462483,3.6477103,-0.7820576735
H,0,0.6498952509,2.1070161968,-1.4526240954
C,0,1.7293068757,1.9412918444,0.4255683858
H,0,1.1653145266,1.3310242362,1.1410519129
H,0,2.2503353109,2.6819964086,1.0472406043
C,0,2.7692367909,1.0697685076,-0.3066433857
H,0,3.7079393874,1.6309352859,-0.3971049078
C,0,3.0482696597,-0.2795197952,0.3632742177
C,0,2.0313406545,-1.373332447,0.0162216407
H,0,2.302454697,-2.3050724291,0.5339394736
H,0,2.0853276252,-1.5776263892,-1.0654744761
H,0,3.0892448337,-0.1524504073,1.4545764748
C,0,-1.4570810778,1.5657916734,0.0612510589
O,0,-1.7020637905,1.0035337246,-1.0555042799
O,0,-1.8622794878,1.0243282742,1.1366470982
C,0,0.6131515108,-0.9798509638,0.375807675
H,0,0.545009653,-0.7483571204,1.4462481391
H,0,0.347679402,-0.0819607812,-0.1849327642
H,0,2.4407960717,0.8671297318,-1.3389932194
S,0,-0.6440399044,-2.262167803,-0.0313220841
S,0,4.6858241549,-0.9685759597,-0.1602314522
Fe,0,-2.482318156,-0.7743086491,-0.0164386884
O,0,-4.5539499813,-0.6078284621,-0.0480339005
H,0,-4.8149493536,0.1979713517,-0.5197492689
H,0,-4.9327037711,-0.5199087783,0.839436532
H,0,5.4308800934,0.0929595525,0.2175026385

{66} Fe^{2+} – $DHLA^{2-}$ (COO, S2) (4-coord)

Charge = 0 Multiplicity = 5
C,0,0.1351996946,3.0481193067,0.6318623331
H,0,0.6180311065,3.4541098716,1.5284910831

```
H,0,-0.0779067292,3.8769531495,-0.0544030927
C,0,-1.1637758595,2.3329447651,1.0212830992
H,0,-1.7056147649,2.9431118983,1.7567377763
H,0,-0.9143170336,1.3922194722,1.5345729217
C,0,-2.0861077141,2.0630914815,-0.1641687119
H,0,-1.5122783212,1.6541807912,-1.0085835523
H,0,-2.4745313691,3.0274160471,-0.5202749736
C,0,-3.2557091903,1.1283523456,0.1775129091
H,0,-4.1922349258,1.5534337132,-0.2078380281
C,0,-3.1279233628,-0.2903199519,-0.3722562877
C,0,-1.9004966912,-1.0836125786,0.1053242794
H,0,-2.1708638266,-2.136990044,0.272707176
H,0,-1.5632933583,-0.7005222663,1.0801568212
H,0,-3.1183407643,-0.2491123042,-1.4711998824
C,0,1.0824351817,2.0729845953,-0.0224287391
O,0,1.6587451898,1.237686217,0.7773292353
O,0,1.2436813344,2.0528485878,-1.2605673746
C,0,-0.7535298259,-1.0695215021,-0.8947882886
H,0,-1.1092650413,-1.4482887617,-1.8616726941
H,0,-0.3812295001,-0.0529180409,-1.0676311421
H,0,-3.3758596891,1.0685157905,1.271027996
S,0,0.6501323026,-2.1439707583,-0.3533117123
S,0,-4.6173267489,-1.2848371498,0.1044207741
Fe,0,2.2640812193,-0.5161517002,0.0025351902
O,0,3.5822079256,-1.3186335412,1.5197297217
O,0,3.9922297016,-0.1843304452,-1.2463100194
H,0,4.7521863788,0.0840861963,-0.7101339249
H,0,3.8304474045,0.5547584655,-1.8500810708
H,0,3.2048963775,-1.9780361941,2.118057232
H,0,4.3837630292,-1.7220598806,1.1574655358
H,0,-5.5462211297,-0.3847815748,-0.2882565897
```

{67} $[Fe(H_2O)_5]^{3+}$

```
Charge = 3 Multiplicity = 6
Fe,0,0.0576847011,0.017741104,-0.0790915164
O,0,-0.6736269821,1.919786101,-0.4622943124
H,0,-1.1426980674,2.2405212802,0.3266206755
O,0,0.6228431835,-1.9228812463,-0.4223189453
H,0,1.5841564426,-2.0055609918,-0.5384716695
O,0,2.0300559069,0.6963658642,-0.2973712207
H,0,2.1839252145,1.382767201,0.3738336357
O,0,0.059112638,0.0362849274,1.9358810525
H,0,-0.4431178443,-0.7113288825,2.303180266
O,0,-1.8467991102,-0.6696109814,-0.4066373572
H,0,-2.5303064006,-0.0046923575,-0.2234336363
H,0,-2.0669647435,-1.4527319325,0.1250819427
H,0,0.2127903363,-2.3157616484,-1.2101666532
H,0,0.9501630755,-0.0055024596,2.322970774
H,0,2.143099363,1.1404081844,-1.1540417384
H,0,-1.3333347132,1.9153508379,-1.176869297
```

{68} $[Fe(H_2O)_5]^{3+}$ (low spin)

```
Charge = 3 Multiplicity = 2
Fe,0,-0.0434668854,0.0087397783,-0.1970556292
O,0,-1.2651692014,1.5402436356,-0.2400881631
H,0,-1.785004943,1.610231698,0.5802007454
O,0,1.1812071404,-1.4909905411,-0.2668521218
H,0,2.0141441528,-1.2992886757,0.2015019601
O,0,1.3490407743,1.2665177393,-0.446102075
H,0,2.1828197768,1.071476262,0.0178328105
O,0,-0.1529905576,-0.1321053633,1.7149127861
H,0,0.571868483,-0.6563727403,2.1034637116
O,0,-1.4319770165,-1.2729420221,-0.43832516
H,0,-2.2722661797,-1.0085434768,-0.0209244873
```

H,0,-1.2014785745,-2.1507492903,-0.0827384296
H,0,1.4292297703,-1.6761131872,-1.1898533742
H,0,-0.1235631683,0.7465460662,2.1378298643
H,0,1.0999300411,2.1838406269,-0.2327708431
H,0,-1.9212136122,1.4167844903,-0.9501745945

{69} $[Fe(H_2O)_4]^{3+}$

Charge = 3 Multiplicity = 6
Fe,0,0.0377956735,-0.0892735868,-0.1204326784
O,0,-1.6207005828,-0.8713848304,-0.9180730084
H,0,-2.4267283388,-0.4387717798,-0.5867820294
O,0,1.8339237542,-0.92756099,0.0409134135
H,0,2.5433308338,-0.2771301911,-0.1036009274
O,0,0.3304200349,1.8141721089,-0.7097214218
H,0,-0.2455905833,2.4348496474,-0.2308547662
O,0,-0.5653784564,0.1783079734,1.7846919289
H,0,-1.5198906684,0.3521710407,1.852608175
H,0,-0.1087545574,0.9257134446,2.207777491
H,0,1.9835040938,-1.6457307802,-0.5987734643
H,0,0.1659236585,1.9534837467,-1.6581970381
H,0,-1.7256878615,-1.8239268034,-0.7517396744

{70} $[Fe(H_2O)_4]^{3+}$ (low spin)

Charge = 3 Multiplicity = 2
Fe,0,0.0080810159,-0.0171032379,-0.0165889463
O,0,-1.3945301456,1.2619680149,-0.183949101
H,0,-1.2906590871,2.0051202977,0.4405627784
O,0,1.414483324,-1.2892158315,0.1711866429
H,0,2.302049522,-0.9019185103,0.052930862
O,0,1.2718883359,1.4060638905,0.0968161732
H,0,1.252393091,1.844606757,0.9674042507
O,0,-1.1914338544,-1.4938365983,-0.1174931122
H,0,-1.8834640289,-1.3695546513,-0.7942417054
H,0,-1.6484063757,-1.6399623535,0.7317792091
H,0,1.3320481194,-2.0243124013,-0.4662185158
H,0,1.1478804326,2.0968011728,-0.5798274336
H,0,-2.294438349,0.9070394512,-0.0577971018

{1B} Cu^{2+} – $DHLA^{2-}$ (CO, S2 *cis*)

Charge = 0 Multiplicity = 2
C,0,0.1881871266,3.201390954,0.464876759
H,0,0.652579455,3.7183233984,1.3172790111
H,0,0.1150768648,3.9477723991,-0.3373648852
C,0,-1.1797008435,2.6599390066,0.8421752659
H,0,-1.7733345608,3.4588921772,1.3065690473
H,0,-1.0564366303,1.8851807578,1.6112311259
C,0,-1.9289784309,2.0779205871,-0.3558910498
H,0,-1.2022733037,1.5783958249,-1.0115433115
H,0,-2.345618096,2.8968801396,-0.9579193416
C,0,-3.0352923314,1.0862017622,0.0305395173
H,0,-4.0251305033,1.5438375285,-0.1001659934
C,0,-2.9897922569,-0.2127746565,-0.7742650681
C,0,-1.6760994413,-0.9913768693,-0.6800000647
H,0,-0.9077756781,-0.4321292967,-1.2345105247
H,0,-1.7874584326,-1.946055248,-1.2150178951
H,0,-3.1681838025,0.0214737798,-1.8326532194
C,0,1.1908114928,2.1532231958,0.0219946032
O,0,2.1606611389,2.5214102732,-0.6885932338
O,0,0.9971760982,0.9590234138,0.4330576721
C,0,-1.1649523751,-1.2578243429,0.7248124848
H,0,-1.0500093636,-0.3267522482,1.2903427305
H,0,-1.8708144868,-1.8885640047,1.2817833888
H,0,-2.9571128616,0.8428199612,1.1011392302

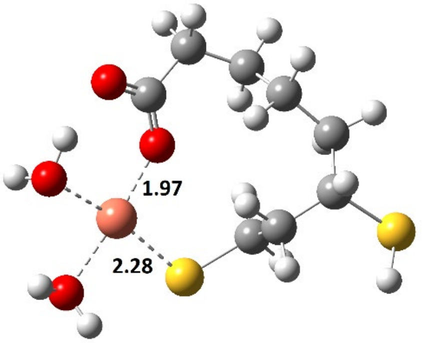

```
S,0,0.4395317159,-2.139661452,0.7546914834
S,0,-4.4362975509,-1.2274088624,-0.2115242354
H,0,-4.1677757172,-2.3069618516,-0.9783836233
Cu,0,2.0304885352,-0.6763195047,0.0360134605
O,0,3.2826452441,-2.3449661821,-0.1377588408
H,0,2.7888602476,-3.1111761775,-0.4664264414
H,0,3.9357643059,-2.1494691305,-0.8266162629
O,0,3.6408911072,0.3426563538,-0.818412753
H,0,4.3588361879,0.3673533858,-0.1703854505
H,0,3.2388021465,1.2540529285,-0.8233195856
```

{2B} Cu^{2+} – $DHLA^{2-}$ (CO, S2 *trans*)

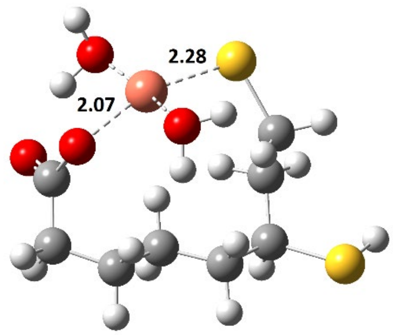


```
Charge = 0 Multiplicity = 2
C,0,1.7345503182,2.9459338615,0.0602233079
H,0,2.3371855133,3.4780628715,0.8080290989
H,0,1.7515455383,3.5374777247,-0.863127968
C,0,0.3034176017,2.7690587141,0.5681670466
H,0,-0.1638502459,3.7563707466,0.6857340264
H,0,0.3258284474,2.3118093711,1.5692618028
C,0,-0.5333589847,1.8977245478,-0.3573499338
H,0,0.0037720504,0.9489574341,-0.5226840617
H,0,-0.6235738543,2.3742696882,-1.3470058786
C,0,-1.9134048378,1.588564644,0.2028986436
H,0,-2.4647685002,2.5275348591,0.3616286249
C,0,-2.7395368893,0.6784197774,-0.7038682471
C,0,-2.0542715325,-0.63111871,-1.0877161622
H,0,-1.1516530757,-0.3712935243,-1.6586906026
H,0,-2.695971037,-1.1824331162,-1.7894100726
H,0,-2.9643506361,1.2261331273,-1.6289306994
C,0,2.3812615896,1.6054644478,-0.2108201938
O,0,2.8952396447,1.3740270241,-1.335595189
O,0,2.3272618016,0.7420065683,0.7333087885
C,0,-1.6747691296,-1.5356403654,0.0812356209
H,0,-1.4926006158,-0.9488125332,0.9893876218
H,0,-2.4950848249,-2.2210374036,0.3261115709
H,0,-1.7998569459,1.1310944228,1.1994393407
S,0,-0.1940567637,-2.5844682242,-0.236846356
S,0,-4.3598602216,0.3962975824,0.1506623391
H,0,-4.8725868529,-0.4380957355,-0.7804851406
Cu,0,1.4121503666,-1.0662263827,0.323433753
O,0,0.7814422962,-0.8914473511,2.2644886796
H,0,0.3568164137,-0.023730226,2.3591908193
H,0,0.1034273786,-1.545681777,2.492416883
O,0,2.5140611947,-1.308794184,-1.3961078109
H,0,3.2872185118,-1.843536677,-1.1638954321
H,0,2.8481442813,-0.3793462029,-1.4974712193
```

{3B} Cu^{2+} – $DHLA^{2-}$ (CO)

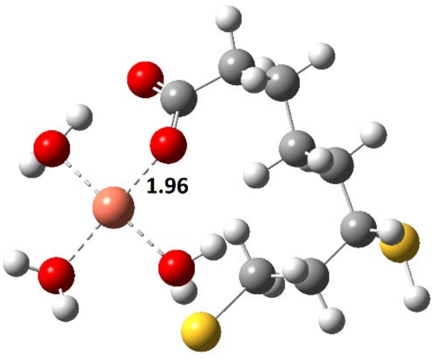


```
Charge = 0 Multiplicity = 2
C,0,0.6281698929,3.2967941019,-0.4891239342
H,0,0.9868739562,4.2672622631,-0.1280891892
H,0,0.6336913318,3.3039159132,-1.5854437964
C,0,-0.790447185,3.0374873535,0.0292965527
H,0,-1.4336062436,3.8797423799,-0.2595067814
H,0,-0.7728809539,3.0209735873,1.1298578574
C,0,-1.3823917419,1.7359333414,-0.4915883366
H,0,-0.673985305,0.9126339241,-0.3104484358
H,0,-1.5031568657,1.7990052752,-1.5856976597
C,0,-2.7130370527,1.3990429909,0.1648629067
H,0,-3.3835326458,2.2698270953,0.1045652386
C,0,-3.4072129772,0.1987829301,-0.4706453573
C,0,-2.5382171682,-1.0501516157,-0.6073921792
H,0,-1.7738747648,-0.8329735878,-1.3709234366
H,0,-3.1466935127,-1.8656365315,-1.0269774727
```

H,0,-3.7438223683,0.4882769717,-1.4756378826
C,0,1.5513882937,2.2203551665,0.0231442726
O,0,1.9182425811,2.2511756705,1.2213355632
O,0,1.862705771,1.2889120253,-0.8064394555
C,0,-1.8501502901,-1.538433013,0.6563589797
H,0,-1.2492794046,-0.7299946162,1.0975724476
H,0,-2.6032053301,-1.8128991211,1.407227883
H,0,-2.545107855,1.2125757037,1.2379456988
S,0,-0.7533720013,-2.9907024586,0.3524865525
S,0,-4.9448128932,-0.115008534,0.5171962202
H,0,-5.3914644594,-1.1451401586,-0.2344342769
Cu,0,2.4654871706,-0.4791291293,-0.2193135758
O,0,1.0831976768,-1.316852107,-1.383208724
H,0,0.4770655979,-1.9175679508,-0.8087252492
H,0,0.5113853696,-0.6090330118,-1.7202268169
O,0,3.187322501,-2.3403441823,0.1395500054
H,0,3.1166777578,-2.8719798924,-0.6678918569
H,0,4.1353147917,-2.2887568642,0.3354605589
O,0,3.6863821177,0.2791675024,1.1902771864
H,0,3.6870858791,-0.3267080918,1.9459307641
H,0,3.1243673297,1.05937667,1.4454547292

{4B} Cu^{+} – $DHLA^{2-}$ (S2)

Charge = -1 Multiplicity = 1
C,0,0.3908925864,3.3815800309,0.4355555057
H,0,0.791355931,4.0280050141,1.2277501445
H,0,0.2064922714,4.0066630051,-0.4470797558
C,0,-0.9092704377,2.7316298889,0.9025196297
H,0,-1.6489488212,3.5126687967,1.1283887675
H,0,-0.7287300197,2.1896258473,1.8434326194
C,0,-1.4696477945,1.7613913009,-0.1261232998
H,0,-0.6895774887,1.0234456004,-0.3719800208
H,0,-1.6921886388,2.2930345222,-1.0657205072
C,0,-2.7119173428,1.0363303424,0.3699781325
H,0,-3.5203299644,1.7639853868,0.5364305038
C,0,-3.2005570384,-0.0440904895,-0.5893498848
C,0,-2.1344969457,-1.0631362801,-0.9874306875
H,0,-1.4049620713,-0.5464820192,-1.6307921472
H,0,-2.5905620742,-1.8366458463,-1.622988363
H,0,-3.5684103103,0.4377247917,-1.5054495062
C,0,1.4398799607,2.3343946187,0.1004012462
O,0,1.8827788697,2.2994535591,-1.0933856783
O,0,1.8034996531,1.5420009786,1.0109341445
C,0,-1.3922188063,-1.7249567786,0.1602760986
H,0,-0.9858393454,-0.976989303,0.8523307584
H,0,-2.074086959,-2.3605280311,0.7398182502
H,0,-2.495953046,0.5880100979,1.3530090966
S,0,-0.0076873146,-2.8077684868,-0.4128709566
S,0,-4.6726643277,-0.8394001449,0.2094444717
H,0,-4.9147700593,-1.7283004843,-0.7789555125
Cu,0,1.4544416654,-1.3439740399,-1.0192571131
O,0,4.1142353401,-1.1233251197,0.7547693419
H,0,3.2599684797,-1.5825763425,0.7938371807
H,0,4.7630097249,-1.8268180006,0.6212759184
O,0,2.8901406676,-0.1217055097,-1.5581170912
H,0,3.548333043,-0.3066345112,-0.8555996552
H,0,2.565065613,0.8086956056,-1.3792976309

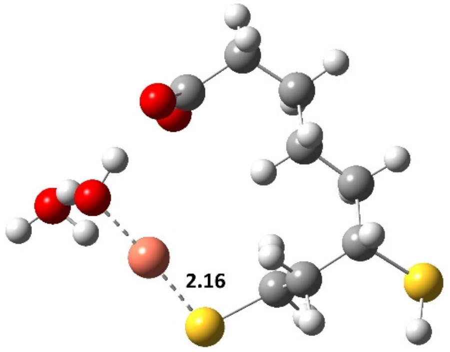

{5B} Cu^{+} – $DHLA^{2-}$ (CO, S2)

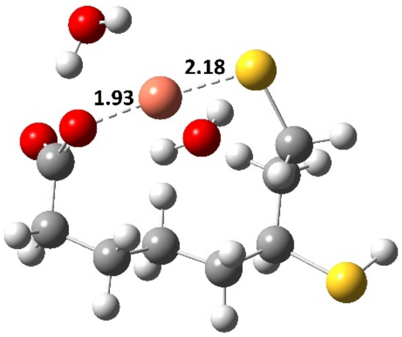


```
Charge = -1 Multiplicity = 1
C,0,1.7523780558,2.8704482118,0.1478215633
H,0,2.3574413483,3.2880302641,0.9636394898
H,0,1.7731793145,3.5835581475,-0.6850435679
C,0,0.3192846961,2.643556205,0.6282588891
H,0,-0.1230881125,3.6090706667,0.9102257033
H,0,0.3354810874,2.0285577366,1.5411450277
C,0,-0.542803147,1.9530437047,-0.4187740499
H,0,-0.0349797876,1.0236043349,-0.7245288291
H,0,-0.6127336198,2.5764819024,-1.3242115969
C,0,-1.935979203,1.6098932732,0.0918294301
H,0,-2.5202682157,2.5344438298,0.212991339
C,0,-2.6939898446,0.6522192194,-0.8253151666
C,0,-2.025639491,-0.7087985923,-1.0170748629
H,0,-1.0573949265,-0.5295861594,-1.5048116577
H,0,-2.6122308014,-1.2949321265,-1.738825611
H,0,-2.7975167169,1.1242083556,-1.8120674059
C,0,2.3979391555,1.5776312955,-0.3012939849
O,0,2.8561667112,1.4810900888,-1.4629895693
O,0,2.4353738656,0.621276402,0.5595803416
C,0,-1.8117679399,-1.5186372026,0.261167236
H,0,-1.8414855006,-0.8717035657,1.1452007725
H,0,-2.6164379458,-2.2518690492,0.3912208912
H,0,-1.8521077282,1.1711816791,1.0988599853
S,0,-0.2296232525,-2.4843593605,0.3088075479
S,0,-4.40797652,0.486115471,-0.1376668654
H,0,-4.8289610692,-0.4435146712,-1.0238534904
Cu,0,1.234765455,-0.8711639335,0.313765498
O,0,0.2287558811,-0.8273263572,3.220725248
H,0,0.741061143,-0.1049233216,2.828996577
H,0,0.1172230956,-1.4461794461,2.4693997813
O,0,3.2448129194,-1.2008482155,-2.1625954691
H,0,2.7814502513,-1.6996990512,-1.4741014593
H,0,3.1394388423,-0.2633247356,-1.8848687349
```

{6B} Cu^{+} – $DHLA^{2-}$ (CO)

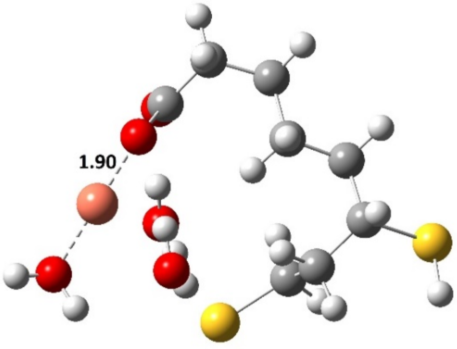


```
Charge = -1 Multiplicity = 1
C,0,0.7114340644,3.3204637342,-0.6852039284
H,0,1.1145195357,4.2791588631,-0.3369442029
H,0,0.6764934446,3.3370484004,-1.7809332702
C,0,-0.690446532,3.1062981973,-0.1108413017
H,0,-1.3447282849,3.9263188282,-0.436702076
H,0,-0.6402488781,3.1617939055,0.9872899755
C,0,-1.2866792288,1.76786706,-0.5190953912
H,0,-0.5612848417,0.9682036503,-0.2953880093
H,0,-1.4371894805,1.7426659674,-1.6112649099
C,0,-2.5921792778,1.4664344805,0.2021730573
H,0,-3.3008290194,2.2939524932,0.0465820951
C,0,-3.2429662136,0.1624999414,-0.2525592808
C,0,-2.2937422604,-1.0317545976,-0.3024408575
H,0,-1.5801008865,-0.8339265755,-1.1163751203
H,0,-2.8514927401,-1.927895053,-0.6141349436
H,0,-3.6510150069,0.3119563978,-1.2616044003
C,0,1.6263813544,2.2123645904,-0.2177235286
O,0,1.9127540998,2.1314328177,0.9990265145
O,0,2.0222899341,1.3744323184,-1.1064937593
C,0,-1.5200523124,-1.3327198163,0.971402931
H,0,-1.1018107316,-0.4060404448,1.3907119184
H,0,-2.1981659132,-1.7374808403,1.7340991813
H,0,-2.3953367844,1.4300546872,1.2857865702
S,0,-0.1371554279,-2.5273405679,0.7029100153
S,0,-4.6987449631,-0.1267782096,0.8585583426
H,0,-5.1017910316,-1.2763954634,0.2741692075
```

Cu,0,2.8393074357,-0.2372012942,-0.5136382663
O,0,0.7338818489,-1.5839414327,-2.2526670071
H,0,0.4922671803,-1.8474119355,-1.3271509886
H,0,1.0108418781,-0.6597819517,-2.1692611956
O,0,3.6850469776,-1.934502904,-0.0593666423
H,0,3.0593053963,-2.6471301717,-0.26249681
H,0,4.4531900805,-2.0978999567,-0.6261742349
O,0,1.7050996083,-0.4121178594,2.2937123335
H,0,1.2259337011,-1.0677531163,1.7315295444
H,0,1.8123202752,0.3850558575,1.7373174384

{7B} $[Cu(H_2O)_4]^{2+}$

Charge = 2 Multiplicity = 2
Cu,0,-0.0008745318,-0.0004430572,-0.008511486
O,0,1.9296586833,-0.6095539622,0.1145416237
H,0,1.9892280176,-1.5599530382,0.2961471786
H,0,2.363878655,-0.4854416942,-0.7435851977
O,0,-0.7823294525,-1.8308140431,0.1508391097
H,0,-1.4814914775,-1.9526161795,-0.5103075312
H,0,-0.1308174833,-2.5260041538,-0.0259879667
O,0,0.781061072,1.8300158208,-0.1677257287
H,0,0.1288718159,2.5249828241,0.0078623632
H,0,1.478773675,1.9523789754,0.4948780051
O,0,-1.9316598119,0.6101937726,-0.0994012734
H,0,-2.348977078,0.4876953008,0.7672845392
H,0,-1.9938510839,1.5602834346,-0.2816956359

{8B} $[Cu(H_2O)_2]^{+} \cdot 2H_2O$

Charge = 1 Multiplicity = 1
Cu,0,-0.0150246489,0.004827353,0.2021316387
O,0,-1.5671853045,1.1347602591,0.3395659935
H,0,-2.3558777295,0.6126132621,0.0478643311
H,0,-1.4960282071,1.8799637495,-0.2731379023
O,0,1.5286621347,-1.1340432191,0.0637883831
H,0,1.6785435142,-1.6033252741,0.8963760312
H,0,2.3291708361,-0.5712824441,-0.0849367109
O,0,3.5793585102,0.5642673309,-0.4642124812
H,0,4.4392630389,0.1318685899,-0.5516384029
O,0,-3.6051425655,-0.5201357484,-0.3578636381
H,0,-4.4634959937,-0.2200607718,-0.0302706959
H,0,-3.7128722623,-0.5680327938,-1.3171832106
H,0,3.6962356773,1.2024287069,0.2519456646

{9B} Cu^{2+} – LA^{-} (CO)

Charge = 1 Multiplicity = 2
C,0,7.3253199299,0.522373743,0.171006237
H,0,7.651499656,0.1453817931,1.1476686196
H,0,8.1388004809,1.0998350707,-0.2804914664
C,0,6.036011762,1.3116182106,0.2606845766
H,0,5.8321374177,1.7730908782,-0.7154648912
H,0,6.13373133,2.1221418417,0.9948013529
C,0,4.8471321659,0.4313020516,0.6493216306
H,0,4.7667245953,0.3666473979,1.7430431922
S,0,6.9726453098,-0.8897465488,-0.9341867764
S,0,5.2107324028,-1.3362341553,0.1367140562
C,0,3.5387990065,0.9316319242,0.06630819
H,0,3.4436490234,1.9971404073,0.3350415398
H,0,3.5992402081,0.8904143292,-1.0334898677
C,0,2.3146811425,0.1784242862,0.5548564517
H,0,2.392897805,-0.8811092904,0.2598447778
H,0,2.2949899076,0.1899332447,1.6567785969
C,0,1.0113196283,0.7505684891,0.0196650521
H,0,1.0483098795,0.7891744285,-1.0795278732

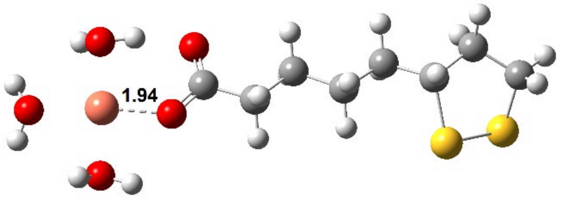

H,0,0.8958518201,1.7887316995,0.3627297172
C,0,-0.1775828464,-0.0798660464,0.4629084556
H,0,-0.0730845308,-1.1196499897,0.1235831962
H,0,-0.215897049,-0.1367167238,1.5617164858
C,0,-1.5310878688,0.4113542843,0.0177456773
O,0,-2.492998736,-0.4112218946,0.2484343718
O,0,-1.6693712175,1.5354781019,-0.5123300197
O,0,-6.4023529195,-0.1663788221,-0.3461708283
O,0,-4.2840839261,1.7401233873,-0.612642123
H,0,-4.6769224649,1.8477815659,-1.4913675117
H,0,-3.2967050303,1.8459434759,-0.7142380283
H,0,-6.6646407486,-0.9340782619,-0.8769637786
H,0,-6.6832113519,0.6092325633,-0.8550611626
O,0,-4.5887511461,-2.1223289324,0.3937590832
H,0,-3.778897102,-2.4212698046,0.8352545894
H,0,-5.2994081525,-2.2416278569,1.0423565829
Cu,0,-4.3900739211,-0.1743440468,-0.0874583856

{10B} Cu^{+} – LA^{-} (CO)

Charge = 0 Multiplicity = 1

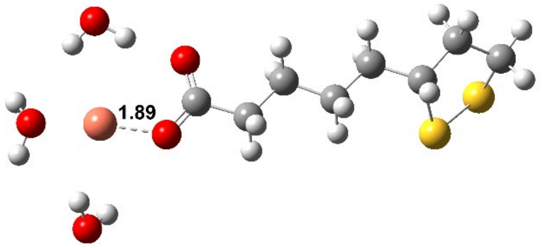

C,0,-6.8708756026,-0.3431092252,0.4174955447
H,0,-7.2909777832,0.437098112,1.0630793686
H,0,-7.630013064,-1.1140370584,0.2474613748
C,0,-5.5803715668,-0.9164498659,0.9673309439
H,0,-5.3108152994,-1.8047676235,0.3797645277
H,0,-5.7202073578,-1.2411073181,2.0065093158
C,0,-4.4164730294,0.081097825,0.904883695
H,0,-4.3357770658,0.6341121038,1.8505637024
S,0,-6.4466881441,0.3881992895,-1.2031871399
S,0,-4.8288357404,1.4197045437,-0.3380107521
C,0,-3.0970928867,-0.600255466,0.5934042034
H,0,-2.9908415503,-1.4479816286,1.2910580419
H,0,-3.1527747673,-1.0365986652,-0.4176933164
C,0,-1.8812081978,0.3004303005,0.7135303837
H,0,-1.9753962239,1.1464842783,0.012926342
H,0,-1.8518883936,0.7395252616,1.7240942341
C,0,-0.57931264,-0.4357826742,0.4401901417
H,0,-0.6132973669,-0.8803685884,-0.5658861621
H,0,-0.4793331288,-1.2749228069,1.1441217764
C,0,0.6249162239,0.4791324208,0.5534730133
H,0,0.5243711759,1.3374520368,-0.1260136137
H,0,0.6818680325,0.914821197,1.5626952189
C,0,1.9638890293,-0.163056278,0.2798159434
O,0,2.9542111691,0.6546464665,0.2290153969
O,0,2.0618232093,-1.4004195439,0.1321685165
O,0,6.6278361775,-0.2210400456,-0.3468856141
O,0,4.3917497555,-2.8692971779,-0.1998973745
H,0,4.9740299781,-2.3848121124,-0.7987947629
H,0,3.6191811853,-2.2711462329,-0.0852319842
H,0,7.0166448238,0.55889748,-0.7707512336
H,0,6.8237896227,-0.9604853888,-0.940798868
O,0,5.536175557,2.9766496339,-0.2047122157
H,0,4.7900346958,2.5578806064,-0.6574936817
H,0,5.8049504508,2.3105919221,0.4443479289
Cu,0,4.7244807223,0.0755222218,-0.0881608952
